# Comprehensive Cell Biological Investigation of Cytochalasin B Derivatives with Distinct Activities on the Actin Network

**DOI:** 10.1021/acs.jnatprod.4c00676

**Published:** 2024-10-11

**Authors:** Mervic D. Kagho, Katharina Schmidt, Christopher Lambert, Thomas Kaufmann, Lili Jia, Jan Faix, Klemens Rottner, Marc Stadler, Theresia Stradal, Philipp Klahn

**Affiliations:** † Department of Chemistry and Molecular Biology, Division of Organic and Medicinal Chemistry, University of Gothenburg, Medicinaregatan 7B, SE-413 90 Göteborg, Sweden; ^¥^Department of Cell Biology and ^∥^Department of Microbial Drugs, Helmholtz Centre for Infection Research, Inhoffenstrasse 7, D-38124 Braunschweig, Germany; § Institute for Biophysical Chemistry, Hannover Medical School, Carl-Neuberg Strasse 1, D-30625 Hannover, Germany; & Division of Molecular Cell Biology, Zoological Institute, Technische Universität Braunschweig, Spielmannstrasse 7, D-38106 Braunschweig, Germany; △ Institute of Microbiology, Technische Universität Braunschweig, Spielmannstraße 7, D-38106 Braunschweig, Germany

## Abstract

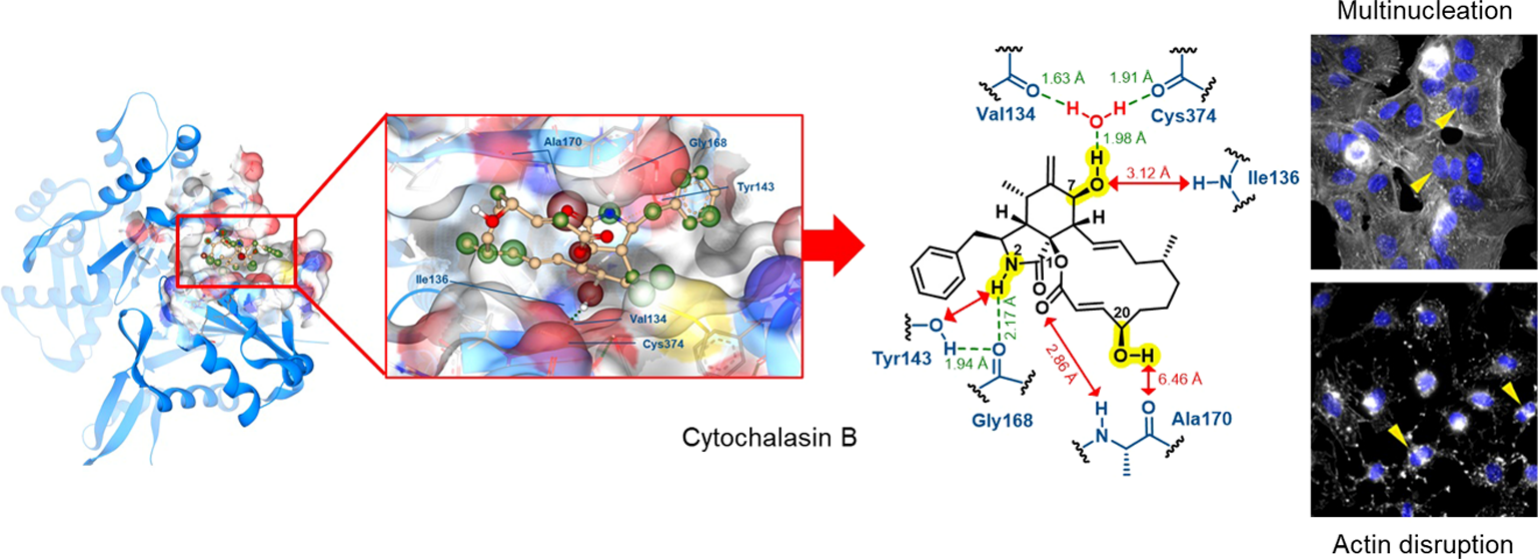

In search of a more comprehensive structure–activity relationship (SAR) regarding the inhibitory effect of cytochalasin B (**2**) on actin polymerization, a virtual docking of **2** onto monomeric actin was conducted. This led to the identification of potentially important functional groups of **2** (i.e., the NH group of the isoindolone core (N-2) and the hydroxy groups at C-7 and C-20) involved in interactions with the residual amino acids of the binding pocket of actin. Chemical modifications of **2** at positions C-7, N-2, and C-20 led to derivatives **3**–**6**, which were analyzed for their bioactivities. Compounds **3**–**5** exhibited reduced or no cytotoxicity in murine L929 fibroblasts compared to that of **2**. Moreover, short- and long-term treatments of human osteosarcoma cells (U-2OS) with **3**–**6** affected the actin network to a variable extent, partially accompanied by the induction of multinucleation. Derivatives displaying acetylation at C-20 and N-2 were subjected to slow intracellular conversion to highly cytotoxic **2**. Together, this study highlights the importance of the hydroxy group at C-7 and the NH function at N-2 for the potency of **2** on the inhibition of actin polymerization.

Cytochalasans are fungal secondary metabolites found in many genera across the Ascomycota with the largest proportion of compounds described for *Diaporthe* and *Chaetomium*. Cytochalasans are biosynthesized by fungal polyketide–nonribosomal peptide synthetases (PKS-NRPS) through fusion of a polyketide chain with an amino acid-derived building block.^1^ The resulting acyclic precursor undergoes a crucial late-stage Diels–Alder (DA) cyclization, forming the tricyclic core structure of the cytochalasan natural product family, which is further modified during the biosynthesis by oxidative rearrangements and cationic cyclizations, contributing to the huge structural diversity of these compounds.^2^ Cytochalasans display a broad spectrum of bioactivities and were ascribed antimicrobial, antiparasitic, or antiviral activities.^3^ The most prominent activity of cytochalasans, however, is the disruption of the actin cytoskeleton, resulting in impairment of cell shape and behavior.^4^

Actin plays a crucial role in most if not all motile processes of eukaryotic cells, including changes of cell shape, cell migration, vesicular trafficking, and cytokinesis.^5^ Actin undergoes dynamic cycles of polymerization and depolymerization. Biochemically, above the so-called critical concentration, monomeric globular actin (G-actin) spontaneously polymerizes into filamentous actin (F-actin) polymers, with preferred addition to one end termed the fast growing (also plus or barbed) end. The other end, termed the minus (or pointed) end, displays a higher critical concentration, causing a reduced polymerization rate as compared with the barbed end. At monomer concentrations in between the critical concentration at the two ends, the barbed and pointed ends thus display polymerization and depolymerization, respectively.^6^ In cells, actin filaments organize into various structures mediated by multiple actin filament binding proteins. This brings about a variety of actin architectures, such as branched filament networks (e.g., found in lamellipodia or vesicular structures), parallel filament bundles (as found in filopodia), or antiparallel bundles (as in stress fibers). Actin filament binding factors display multiple activities, not only including parallel or antiparallel bundling, but also capping of filament ends, filament severing, or even their cross-linking and branching. Actin monomers are tightly regulated as well, for instance by profilin, which aids monomer addition onto filament barbed ends, but also blocks their spontaneous nucleation, whereas the latter is spatially and temporally controlled by distinct classes of nucleators or nucleation promoting factors.^7^

According to the most widely accepted model of actin–cytochalasin interaction, members of this compound family bind actin filament barbed ends, thought to inhibit the addition of new monomers.^8^
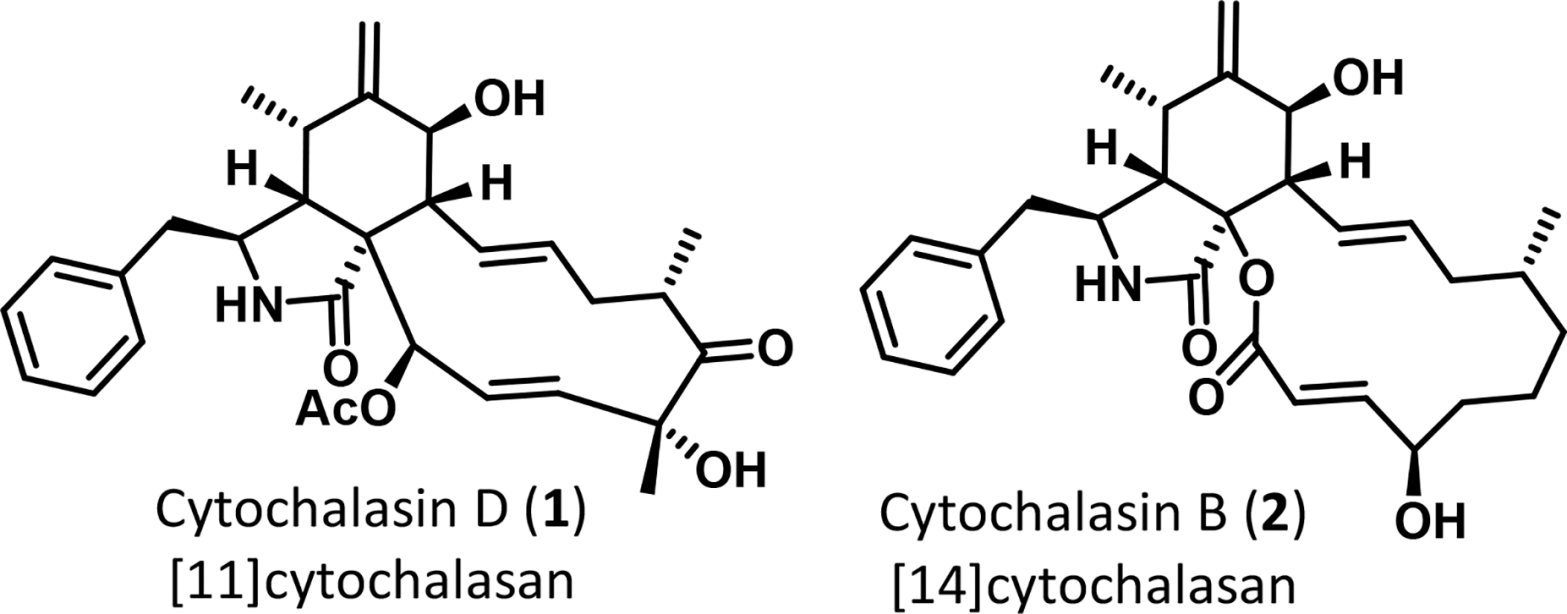


Due to the interference with actin polymerization, cytochalasin-treated cells display compromised, actin assembly dependent structures, coinciding with a high cytotoxicity in cultured cell lines of these compounds ranging from sub-micromolar to nanomolar concentrations.^9^ Hence, successful clinical application would require balancing the projected toxicity and therapeutic benefit. This goal will require an in-depth understanding of how distinct chemical moieties affect the activity of a given cytochalasan. A variety of structure–activity relationship (SAR) studies have been published over the past decades,^4^ attempting to shed light on the complexity of cytochalasan activity and diversity. Conclusions drawn from previous SAR studies include a pivotal role of the C-7 hydroxy group, while the amino acid incorporated in the isoindolone core will likely not affect the activity.

Notably, *bona fide*, detailed structural information on the interaction of cytochalasans with the actin filament barbed end is currently missing. The best approximation of this interaction is derived from a cocrystal of a nonpolymerizable actin variant in complex with cytochalasin D (**1**), published in 2008.^10^ The orientation of **1** in the binding pocket is strongly guided by polar contact and hydrophobic interactions at the back half of the hydrophobic cleft (compare with Figure 1A). However, a comprehensive SAR of other cytochalasans on actin remains a challenging task. For instance, the precise mode of binding of cytochalasin B (**2**), the second among the most frequently employed cytochalasans, to actin remains unknown. For this reason, we conducted a molecular docking of **2** onto the previously described, nonpolymerizable actin variant,^11^ to predict functional groups of this molecule potentially mediating its interaction with actin. We then tested derived hypotheses by semisynthetically modifying these functional groups of **2**, e.g., by acetylation and methylation, with the aim to generate derivatives with potentially altered activities on F-actin organization in cells or cytotoxicity. We thus obtained a precise determination of those positions in the backbone of **2** relevant for its bioactivities, using both cell biological and *in vitro* actin polymerization assays.

**Figure 1:**
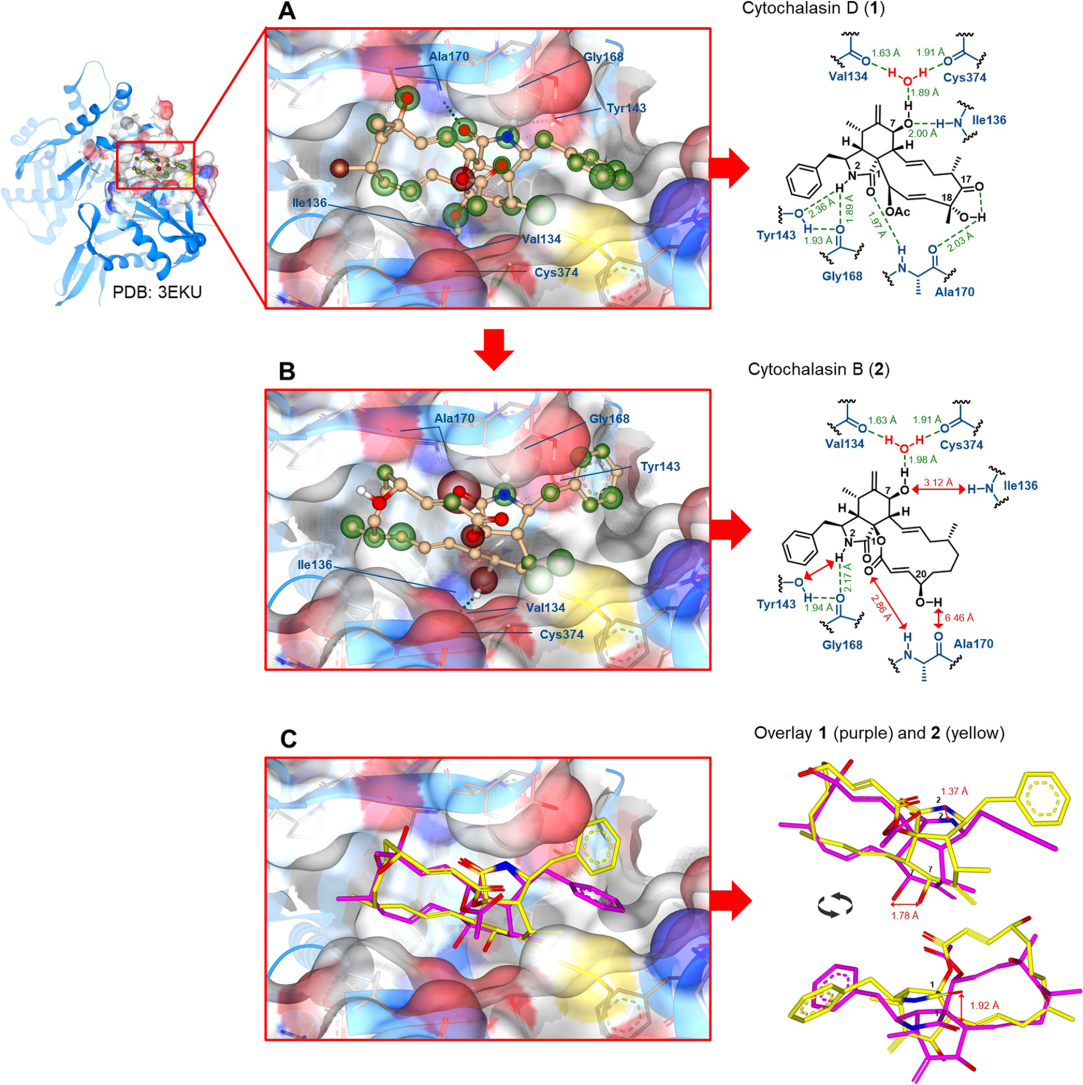
Docking of cytochalasin (**2**) onto monomeric actin: (A) 3D illustration of the cocrystal structure (PDB: 3EKU)^10^ of nonpolymerizable monomeric actin and cytochalasin D (**1**) with the main hydrogen bridge network; (B) 3D illustration of the docking of **2** into the binding pocket of **1** on monomeric actin (PDB: 3EKU);^10^ (C) 3D illustration of the overlay of **1** (purple) and **2** (yellow) within the binding pocket on monomeric actin. Docking was performed and 3D illustrations were generated with SeeSAR version 13.0.5; BioSolveIT GmbH, 2023, www.biosolveit.de/SeeSAR.^18^ Green spheres around atoms indicate overall favorable contributions to Δ*G*_(Hyde)_; red spheres around atoms indicate overall unfavorable contributions to Δ*G*_(Hyde)_.^19^ Hydrogen bridges are indicated by dotted green lines with distances between hydrogen atoms and donor heteroatom given in Å in green. General distances between atoms are given in Å in red. Light gray illustration represents the surface of the binding pocket with elements surrounding the bound cytochalasans in red (oxygen), blue (nitrogen), and yellow (sulfur). Gray shadows represent unoccupied space in the binding pocket. Numbering of atoms in cytochalasans follows the nomenclature applied by Binder et al.^16^

## Results and Discussion

### Virtual Docking of Cytochalasin B (**2**) onto Monomeric Actin

In order to understand the mode of binding of **2** to actin, we generated 724 conformers of an energy-minimized structure of **2** using the conformer ensemble generator Conformator.^12^ A virtual docking of these conformers into the binding pocket of **1** on monomeric G-actin (PDB: 3EKU)^10^ obtained in 2008 by Nair et al. was performed and compared to **1**. The utilized cocrystal structure of **1**(10) is based on a nonpolymerizable actin mutant from *Drosophila melanogaster* bearing two point mutations (A204E/P243 K), referred to as AP-actin,^11^ which is thought to retain all biochemical and structural characteristics compared to tissue-purified G-actin.^13^ It needs to be mentioned that cytochalasans such as **2** and **1** are known to bind the barbed end of F-actin and not to usually interact with G-actin except for the nonpolymerizable AP-actin mutant. Very recently, two cryo-EM structures of the barbed end of F-actin have been reported.^14,15^ However, because our approaches to dock **1** or **2** onto these structures have so far not yielded reasonable results, we decided to proceed with AP-actin for our own docking studies. As described earlier by Nair et al.,^10^**1** binds this actin variant displaying a distinct network of six intramolecular hydrogen bonding interactions with the actin backbone as outlined in Figure 1A. Five of these are mediated by the functional groups in the isoindolone core of **1**. Two hydrogen bonds are formed between the amide NH at N-2^16^ oriented deep into the binding pocket with tyrosine-143 (Tyr143) and glycine-168 (Gly168). Furthermore, the oxo group of **1** at the C-1 position of the isoindolone core serves as a hydrogen bond acceptor for the NH at alanine-170 (Ala170). In addition, the hydroxy group at the C-7 position of the isoindolone core, which points deep into the binding pocket as well, is involved in two hydrogen bonds, one as a hydrogen bond acceptor for the NH at isoleucine-136 (Ile136) and the other as a hydrogen bond donor toward a molecule of water, which is positioned within a local network of hydrogen bonds between valine-134 (Val134) and cysteine-374 (Cys374) of the actin backbone. Finally, one hydrogen bond occurs between the hydroxy group at C-8 of the 11-membered macrocycle of **1** and the oxo moiety of Ala170. An additional intramolecular hydrogen bond with the C-17 oxo group of compound **1** further stabilizes the overall complex.

Our docking of **2** into the same binding pocket resulted in an overall similar mode of binding compared to **1** as outlined in Figures 1B and 1C. However, the more sterically demanding 14-membered macrocycle seemed to not allow **2** to penetrate as deeply into the pocket compared to **1** and furthermore shifted the overall position of **2** in the binding pocket by roughly 1.51–1.78 Å with a huge impact on the hydrogen bond network. In comparison to **1**, the NH group at the N-2 position and the oxygen atom of the oxo group at C-1 of the isoindolone core of **2** are lifted out of the pocket by 1.37 and 1.92 Å, respectively (Figure 1C). The larger distance to Ala170 and Tyr143 prevents the formation of two hydrogen bonds, as seen for **1** (compare Figure 1B and A). Furthermore, the hydroxy group at C-7 of the isoindolone core of **2** is shifted by 1.78 Å away from the NH of the Ile136 (Figure 1C), preventing the formation of a hydrogen bond as seen for **1** (compare Figure 1B and A). Overall, the docking suggested a significantly reduced estimated binding affinity of **2** for actin compared to **1**, in line with the observed diminished bioactivity if comparing **1** and **2**.^17^

Based on this docking, we estimated that the amide NH group at N-2 and the hydroxy function at C-7 will be vital for the activity of **2** on actin, while the hydroxy function at C-20 appeared to be less involved in binding.

### Semisynthetic Derivatization of Cytochalasin B

**Scheme 1:**
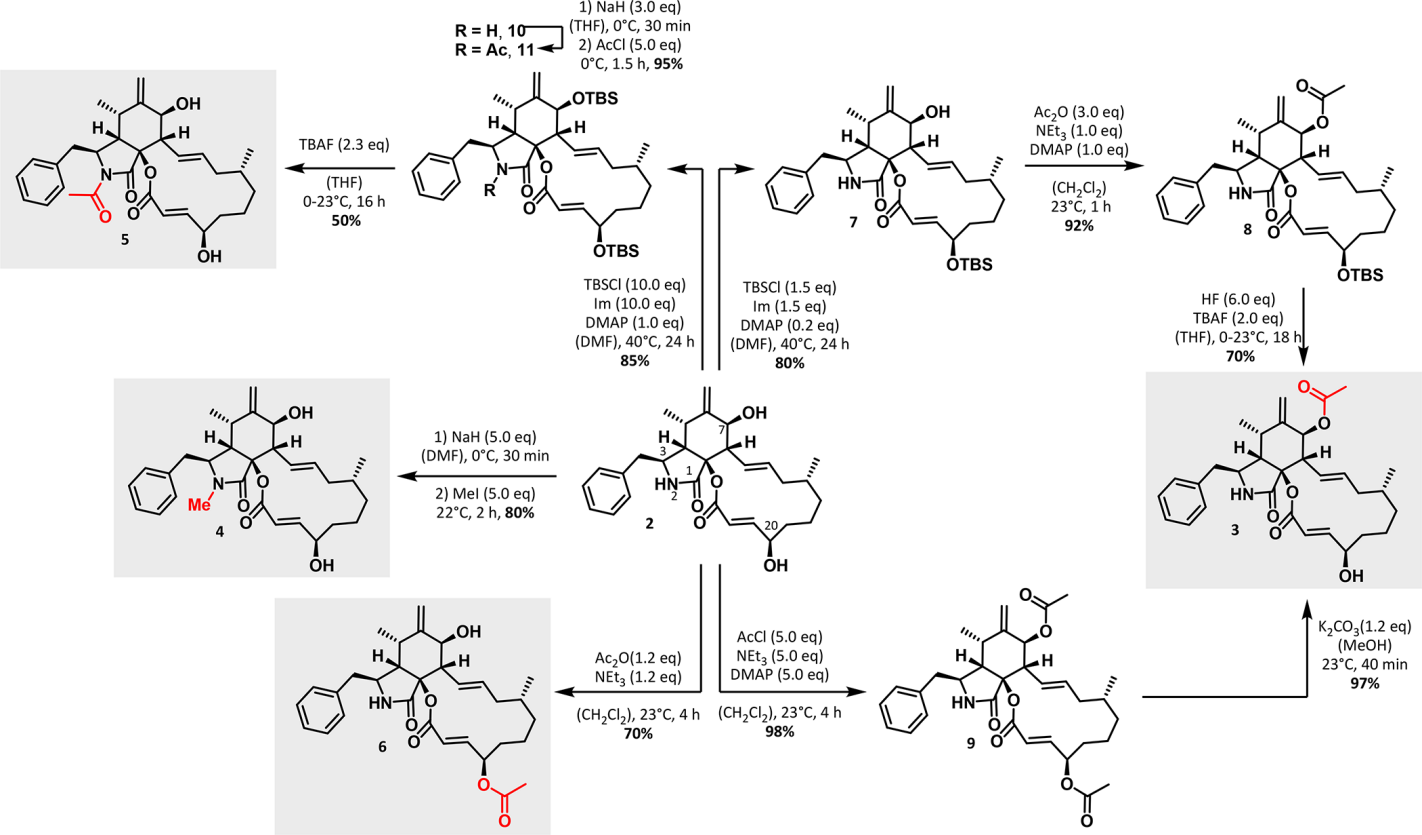
Semisynthetic Modification of **2** toward 7-*O*-Acetyl Cytochalasin B (**3**), *N*-Methyl Cytochalasin B (**4**), *N*-Acetyl Cytochalasin B (**5**), and 20-*O*-Acetyl Cytochalasin B (**6**)

To biologically validate the importance of these moieties for the overall actin binding and cytotoxic activity of **2**, we semisynthetically modified **2** at the N-2 NH as well as the C-7 and C-20 hydroxy functions by methylation and acetylation as outlined in Scheme 1, obtaining the derivatives 7-*O*-acetyl cytochalasin B (**3**), *N*-methyl cytochalasin B (**4**), *N*-acetyl cytochalasin B (**5**), and 20-*O*-acetyl cytochalasin B (**6**).

7-*O*-Acetyl cytochalasin B (**3**) was obtained via two different routes. In the first route, selective *O*-silylation of the C-20 hydroxy function occurred, when **2** was treated with TBSCl in the presence of imidazole and substoichiometric amounts of DMAP at 40 °C, yielding compound **7** in 80% yield. Subsequent reaction with a 3-fold excess of acetic anhydride in the presence of triethylamine and stoichiometric amounts of DMAP led to the formation of 7-acetyl derivative **8** in 92% yield. Finally, treatment with a mixture of HF:TBAF 3:1^20^ afforded **3** in 70% yield and 81% overall yield from **2**. In a second route, double acetylation toward 7,20-*O*,*O*′-diacetyl cytochalasin B (**9**) was achieved in the presence of a 5-fold excess of acetyl chloride with triethylamine and stoichiometric amounts of DMAP in 98% yield. Afterward, treatment of **9** with 1.2 equiv of K_2_CO_3_ in anhydrous MeOH led to selective acetyl cleavage at the C-20 position, yielding **3** in 97% (Scheme 1).

The deshielding of the proton at C-7 from 3.80 ppm (d, 1H) in parental **2** to 5.28 ppm (d, 1H) in **3** in the ^1^H NMR spectra clearly indicated the *O*-acetylation of the C-7 hydroxy group, while the signal for the proton at the C-20 position remained at around 4.46–4.51 ppm (m, 1H). Additionally, HMBC long-range ^4^*J*-coupling between the proton at C-7 and the carbonyl carbon of the introduced acetyl group (5.28 ppm/170.09 ppm, see Figure S17 in the Supporting Information) provided further analytical evidence for *O*-acetylation of the hydroxy function at C-7.

Interestingly, reaction of **2** in the presence of five equivalents of sodium hydride in dry DMF and subsequent treatment with an excess of methyl iodide did not lead to formation of *O*-methylated cytochalasin B derivatives, but instead gave rather selective access to *N*-methyl cytochalasin B (**4**) in 80% yield.

The successful *N*-methylation at position N-2 was proven by the presence of a singlet with an integral corresponding to three hydrogen atoms at 2.83 ppm in the ^1^H NMR spectrum of **4** (Figure S20 in the Supporting Information) and the respective *N*-methyl carbon in the ^13^C NMR spectrum of **4** at 28.52 ppm, exhibiting an HMBC long-range ^4^*J*-coupling with the methylene protons at C-10 (2.74 ppm) (Figure S21). The *N*-acetylation of **2** toward *N*-acetyl cytochalasin B (**5**, Scheme 1) was achieved in three steps. First, conversion of **2** in the presence of a 10-fold excess of TBSCl, imidazole, and stoichiometric amounts of DMAP at 40 °C gave access to the double *O*-silylated intermediate **10** in 85% yield. Reaction of **10** with sodium hydride in anhydrous DMF and subsequent treatment with acetyl chloride furnished *N*-acetylation toward compound **11**. Final TBS deprotection with TBAF gave *N*-acetyl cytochalasin B (**5**) in 50% yield along with 6% of **2**, indicating the lability of the *N*-acetyl function toward basic conditions.

Finally, the reaction of **2** with acetic acid anhydride in the presence of triethylamine led to the selective formation of 20-*O*-acetyl cytochalasin B (**6**) in 70% yield (Scheme 1). **6** was distinguishable from **3** by TLC (hexane:EtOAc 1:1), *R*_f_(**3**): 0.50, and *R*_f_(**6**): 0.47 [UV^254^, CAM].

Furthermore, the deshielding of the C-20 proton (5.49–5.52 ppm, m, 1H)) compared to the C-7 OH proton (3.88 ppm, d, 1H) was taken as proof for *O*-acetylation of the hydroxy function at C-20. Additionally, further analytical evidence for *O*-acetylation of the hydroxy function at C-20 was provided by an HMBC long-range ^4^*J*-coupling between the proton at C-20 and the methyl carbon of the introduced acetyl group (5.51 (5.49–5.52) ppm/20.97 ppm, Figure S34). With derivatives **3**–**6** in hand, we next conducted a comprehensive biological evaluation of the compounds to elucidate the impact of the modifications on their efficacy against actin and their overall cytotoxicity.

### Biological Evaluation of Cytochalasin B Derivatives

First, the antimicrobial effects of compounds **3**, **4**, **5**, and **6** against a variety of bacteria and fungi were examined. These included *Staphylococcus aureus*, *Bacillus subtilis*, *Mycobacterium smegmatis*, *Escherichia coli*, *Pseudomonas aeruginosa*, *Chromobacterium violaceum*, *Acinetobacter baumannii*, *Schizosaccharomyces pombe*, *Pichia anomala*, *Mucor hiemalis*, *Candida albicans*, and *Rhodotorula glutinis*. Compound **3** exhibited weak antibacterial activity against *M. smegmatis* with a minimum inhibitory concentration (MIC) of 66.6 μg/mL. In addition, **6** showed a moderate MIC with 33.3 μg/mL against *S. pombe*. All of the other substances had no antibacterial or antifungal activities against any of the tested microorganisms. Second, **2** and its four derivatives **3**, **4**, **5**, and **6** were evaluated for their cytotoxic effects in two tumor cell lines, namely, mouse connective tissue fibroblasts L929 and human cervix carcinoma cells KB3.1 (Table 1). Compared to their derivatives, **2** showed cytotoxic effects in L929 with an IC_50_ value of 1.3 μM, whereas **5** and **6** exhibited clearly reduced but still moderate cytotoxicities ranging from 4.8 μM to 9.4 μM in both cell lines.

**Table 1:** Cytotoxicity of **2**, **3**, **4**, **5**, and **6** Tested against L929 and KB3.1 Cell Lines^21,22^

	IC_50_ [μM]	
	L929	KB3.1
**2**	1.3	n.t.^b^
**3**	16	27
**4**	NC^a^	43
**5**	9.4	7.9
**6**	4.8	n.t.
Epothilone B	(5.8 ± 1.0) × 10^–4^	(9.5 ± 5.6) × 10^–5^

a NC: no cytotoxic effect, or only weak inhibition of proliferation.

b n.t.: not tested.

In contrast, **3** and **4** were not cytotoxic (IC_50_ > 10 μM) in KB3.1 and L929 cells (Table 1). From this, we concluded that the almost complete loss of cytotoxicity is caused by the incorporation of a methyl group at the N-2 position of the isoindolone core in **4**, whereas the *O*-acetylation at hydroxy groups at C-7 and C-20 in derivatives **3** and **6**, respectively, as well as the *N*-acetylation at N-2 position in **5** seemed to affect cytotoxicity less dramatically (Table 1).

**Figure 2:**
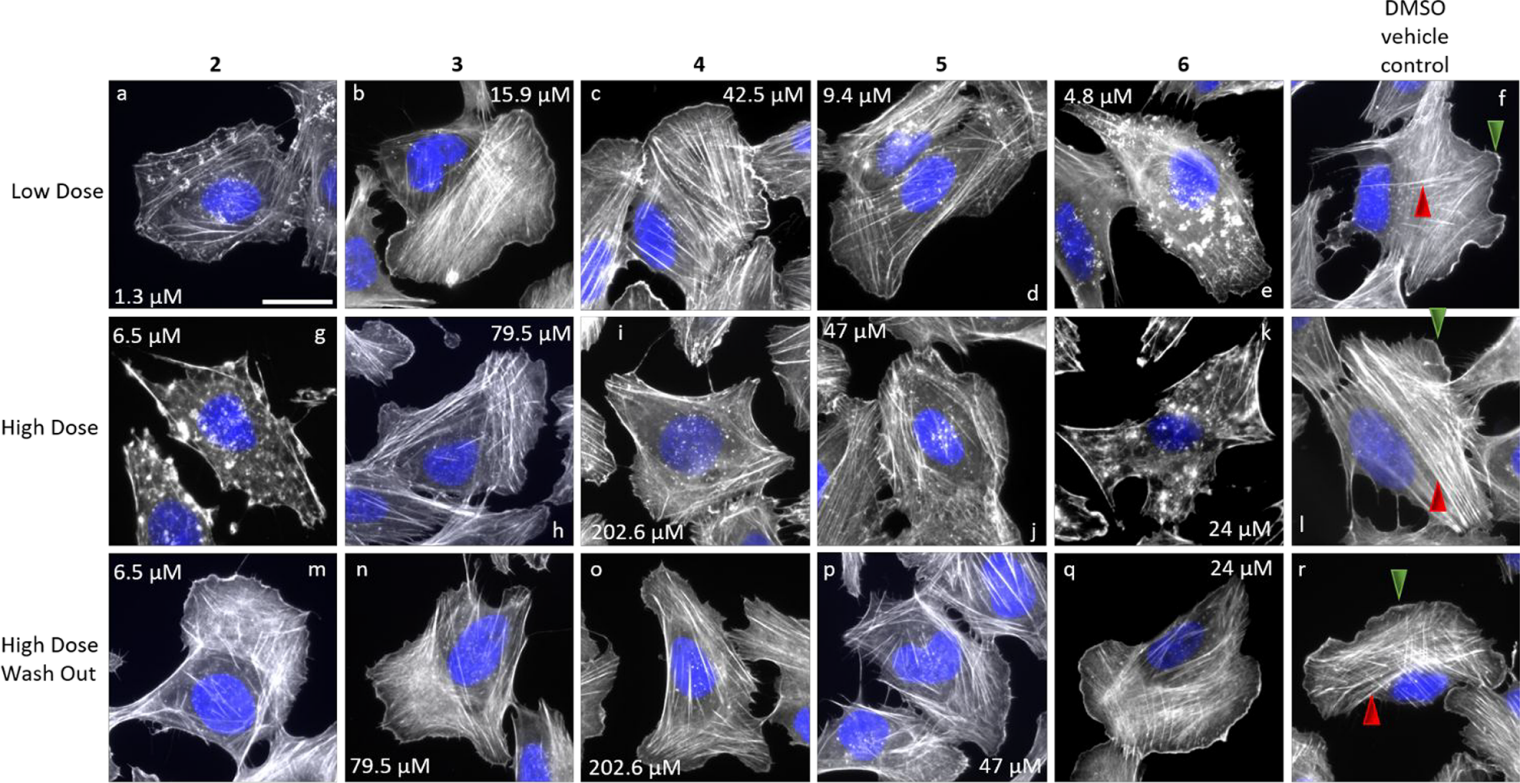
Overlay images of U-2OS cells treated with low and high dose concentrations of compounds **3** (b, h), **4** (c, i), **5** (d, j), and **6** (e, k) as indicated. Compound **2** (a, g) served as a positive control. Compound concentrations were based on previously determined IC_50_ values in L929 mouse fibroblast cells (low dose: 1 × IC_50_, a–f; high dose: 5 × IC_50_, g–l). DMSO (f, l, and r) was used as vehicle control, and a recovery experiment corresponded to high dose treatment (as above) followed by 1 h recovery in full growth medium (high dose washout, m–r). Cells were fixed with paraformaldehyde and stained for their F-actin network using fluorescently labeled phalloidin (white) and their nuclear DNA using DAPI (pseudocolored in blue). Described F-actin rich structures such as lamellipodia (green arrowheads) and stress fibers (red arrowheads) are highlighted in (f, l, and r). At least two independent experiments with two replicates each were performed. The representative scale bar in (a) corresponds to 25 μm.

A well-established *in cellulo* actin disruption assay was employed to study the effects of the derivatives **3**, **4**, **5**, and **6** as opposed to the known effect of **2** on the F-actin network.^17^ For this, the human osteosarcoma cell line U-2OS was treated using concentrations estimated based on previously determined IC_50_ values in murine L929 fibroblasts (Table 1), referred to as low dose (1 × IC_50_), and a 5-fold concentration, referred to as high dose (5 × IC_50_). Reorganization of the F-actin network was also investigated upon high dose treatment, followed by washout and a 1 h recovery phase in fresh medium. The impact on F-actin network organization was visualized using fluorescently labeled phalloidin (Figure 2). Cells treated with DMSO as vehicle control (Figure 2f, l, and r) displayed distinct F-actin structures like lamellipodia, F-actin-rich meshworks at the cell periphery (green arrowheads), and stress fibers, antiparallel, contractile F-actin bundles (red arrowheads). A low dose treatment of cells with **2** and **6** partially led to compromised lamellipodia and a reduction of intermittent, cytoplasmic F-actin (Figure 2a and e), whereas effects of the other derivatives were mostly indiscernible from the DMSO control (Figure 2b–d). Higher concentrations of **2** and **6** caused a complete collapse of the F-actin network, manifesting in the formation of knot-like, F-actin-rich accumulations (Figure 2g and k), whereas **4** and **5** induced only slight and **3** no such structures (Figure 2h–j). In addition, the effects on F-actin structures observed upon high dose treatment of all compounds were fully reversible after a 1 h recovery time (Figure 2m–q).

In conclusion, a correlation between chemically modified positions in the backbone of **2** and actin disruption activity was uncovered, as C-7 and N-2 modified derivatives showed reduced or no actin inhibition activity in this experimental setup. Surprisingly, acetylation of the C-20 position as in **6** reduces cytotoxicity but preserved the activity on actin as observed for **2**.

**Figure 3:**
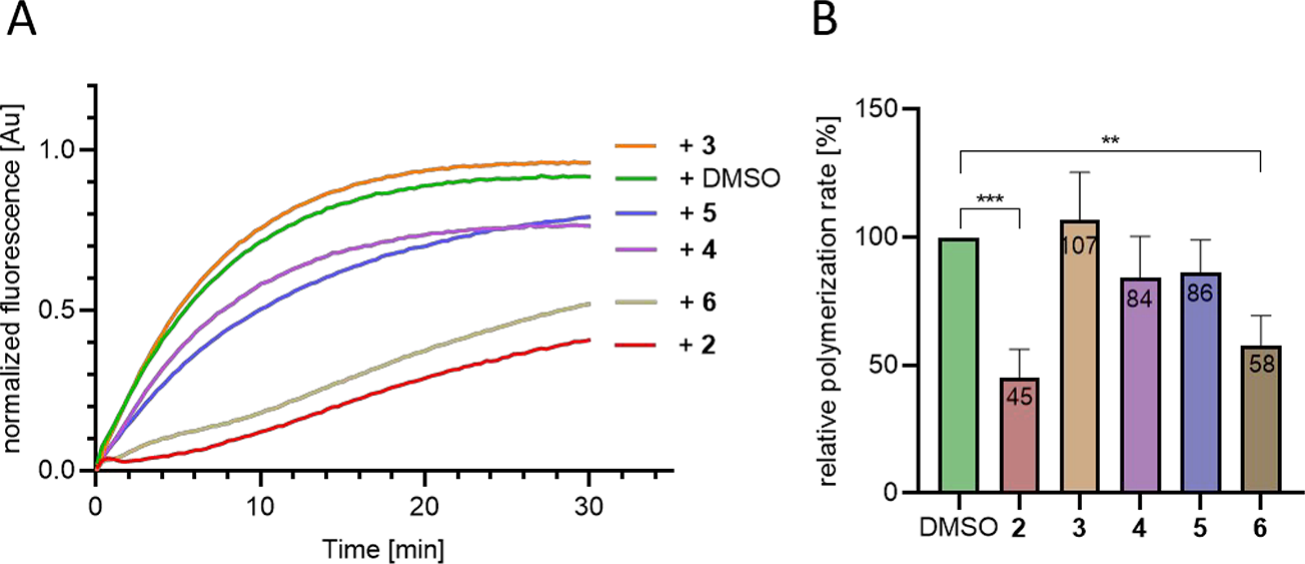
Effects of **2** and derivatives on *in vitro* actin polymerization. (A) Pyrene assays were performed using 2 μM actin supplemented with 5% pyrene-actin and 2 μM **2**–**6**, respectively. Polymerization was initiated by injecting G-actin into a solution containing actin polymerization buffer and 550 nM actin seeds. Normalized fluorescence intensities [Au] of actin polymerization curves were plotted over time. The graph shows the mean normalized fluorescence intensity from at least two independent experiments with two replicates each. (B) Relative actin polymerization rate [%] after 30 min. Data show means ± SD; *n* = 2. ***p < 0.0002, **p < 0.0015, ordinary one-way ANOVA.

To rule out that the reduced *in cellulo* efficacies of **3**–**5** could instead be explained by an altered membrane permeability of modified compounds or their differential effects on other intracellular factors, pyrene actin polymerization assays^23^ were used to analyze potential effects on actin assembly *in vitro* under defined conditions. Globular actin (G-actin) was purified from rabbit skeletal muscle^24^ and fluorescently labeled with pyrene (see description in the Supporting Information). G-actin (2 μM) supplemented with 5% pyrene-labeled actin was added to actin seeds and the respective compound (2 μM) in polymerization buffer to initiate the reaction. Actin polymerization was then assessed by reading out the increasing pyrene fluorescence signal over time (Figure 3). Equimolar amounts of **2** (red) and **6** (brown) significantly reduced the polymerization rate of actin to 45% and 58%, respectively, compared to the DMSO control (green). In contrast, the derivatives **4** and **5** (purple, blue) by trend, but not in a statistically significant fashion, inhibited polymerization (84–86%), while **3** (orange) had no effect or even slightly increased it (107%). These results allowed us to exclude that the reduced membrane permeability of the compounds is causative of the reduced activity *in cellulo*.

Dynamic actin assembly in cells is crucial for many processes such as protrusion (e.g., of lamellipodia) or adhesion.^5^ Thus, we next asked whether these compounds interfered with cell attachment. However, no differences were observed for the five compounds compared to DMSO (data not shown). Of note, however, the experiments revealed a significant reduction of cell size after 24 h treatment (data not shown).

**Figure 4:**
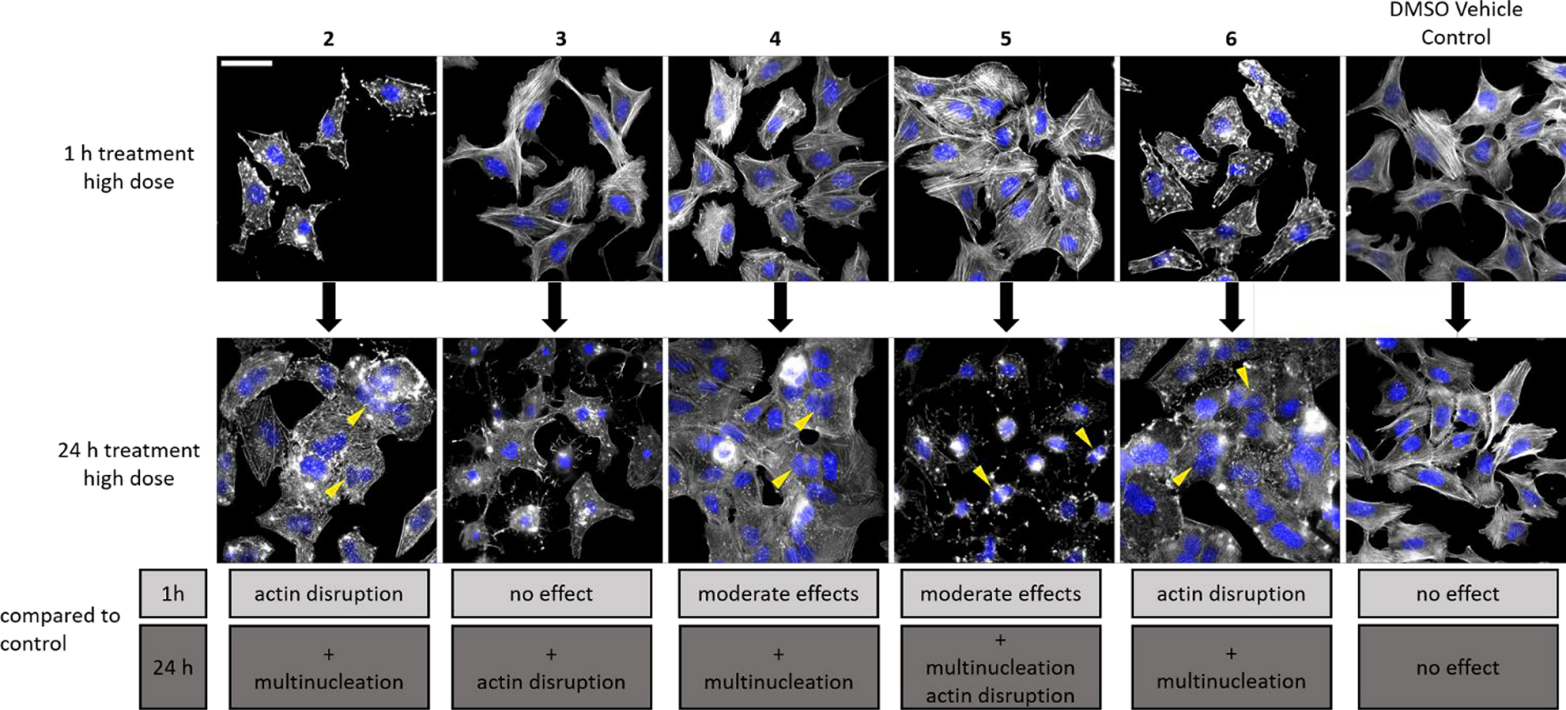
Long-term treatment of U-2OS cells with **3**–**6** influences the degree of actin disruption and induces multinucleation. Compound **2** served as a positive control and DMSO as vehicle control. Cells were treated with high dose concentrations of indicated compounds for 1 h (upper row) and 24 h (lower row), fixed, and stained for F-actin as described before. Multinucleated cells are marked with yellow arrowheads. Differences on actin and nuclei number of the cells between 1 and 24 h treatment are summarized in gray boxes. At least two independent experiments with two replicates each were performed. Scale bar corresponds to 50 μm.

To better understand these long-term effects, we extended the 1 h end point assay to a 24 h high-dose treatment and visualized the actin network as described above (Figure 4).

We noticed massive changes in the amount of multinucleated cells or significant disruption of the actin network for the individual compounds. In case of **2**, the most striking cellular alteration is the formation of multinucleated cells, which was already observed by Carter in 1967,^25^ reminiscent of the outcome of **5** and **6** treatment (for clarification of the effect of **5** on U-2OS cells after 24 h treatment, see Figure S1). Strikingly, the 24 h treatment with **3** evoked altered actin structures, however without inducing multinucleation, whereas **4** caused multinucleation, but left the actin network largely unchanged.

**Figure 5:**
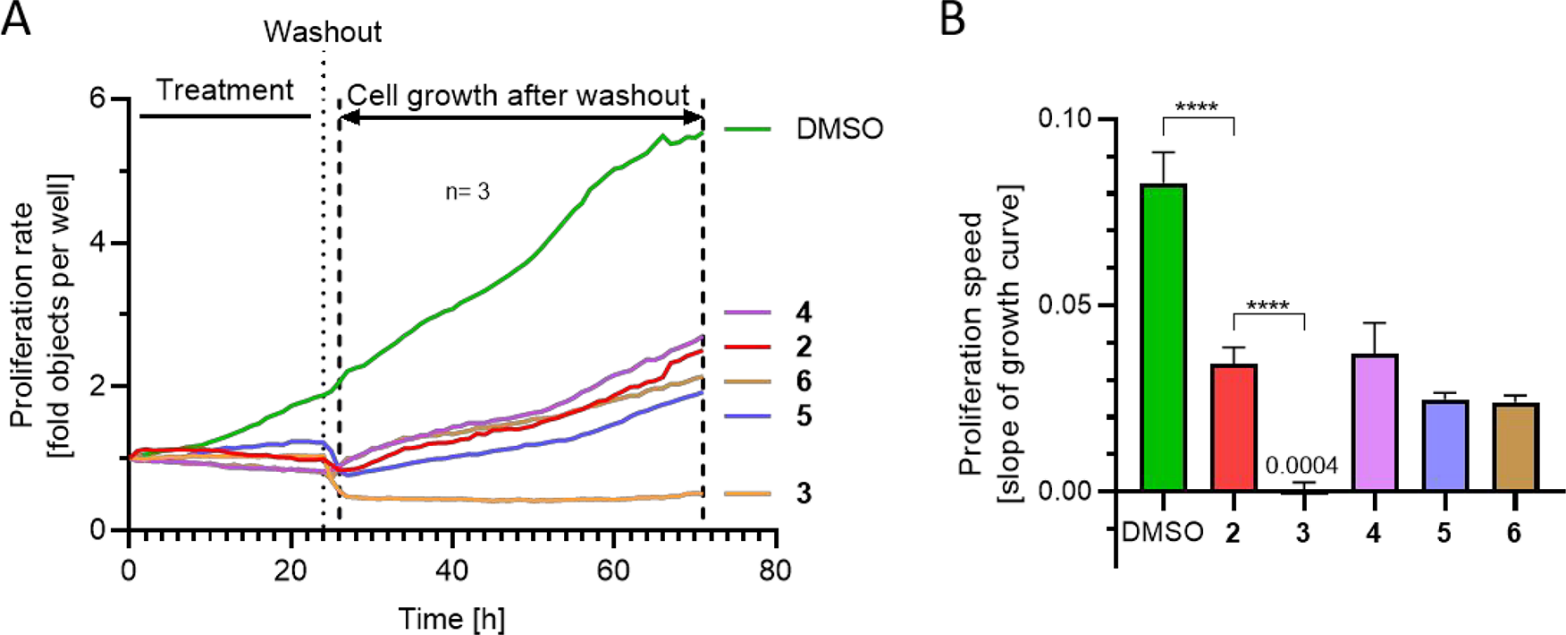
Analysis of cell proliferation during a 24 h high dose treatment followed by a 47 h regeneration phase. (A) Averaged growth curves of U-2OS cells during treatments, as indicated. Proliferation rate was assessed by phase-contrast imaging and automated object counting for 71 h. The graph shows the means from at least three independent experiments with three replicates. (B) Proliferation speed during the recovery phase as determined by calculating the slopes from the growth curves between 26 and 71 h (arrow). Data are means ± SD; *n* = 3. ****p < 0.0001, ordinary one-way ANOVA.

Next, we combined the 24 h treatment of U-2OS cells with a subsequent washout step and an additional recovery period of 47 h to evaluate the ability of the affected actin network to regenerate (Figure 5). The number of cells stagnated during the treatment period with all five compounds and dropped after the washout step, likely due to the loss of detached and damaged cells. After washing, cells treated with **2** and **4**–**6** slowly started to regenerate and proliferate, while recovery of **3**-treated cells was significantly diminished (Figure 5).

Staining procedures revealed a fully recovered actin network for **2** and **4**–**6**, underlining the reversibility of this effect, whereas multinucleation was not fully overcome after 2 days of recovery (Figure S3). In contrast, cell proliferation was completely abolished upon treatment with **3**, although cells harbored a seemingly intact actin network. From this, we concluded that all compounds caused severe long-term effects on cell proliferation, even the “nontoxic” **4**. Due to the strong effects upon long-term treatment with **3**, **5**, and **6**, we aimed to exclude the possibility that these compounds undergo conversion to **2** by intracellular deacetylating enzymes, explaining increased activities. Hence, we attempted to reextract the mentioned compounds from the medium after 24 h cell exposure. Indeed, we could detect an additional peak in the mass and UV spectra corresponding to **2** when analyzing extracts derived from **5** and **6** treatment (Figures S6 and S7), but not for **3**. This indicates that the acetyl groups in **5** and **6** can in fact be cleaved by esterases.^26^ While little conversion of **5** was already observed after 1 h (data not shown), the cleavage of **6** was not detected at that time point. Notwithstanding this, no decomposition of **3** was observed (Figure S5), so we assume that the cellular effects of **3** are inherent to the compound. The reduced actin disruption activity of C-7 *O*-acetylated cytochalasans has been described before,^4,27,28^ whereas the antiproliferative, long-lasting cytostatic activity of **3** observed here deserves further investigations. For instance, **3** might interfere with actin-independent targets as summarized by Lambert et al.^4^ As known for decades, **2** inhibits glucose transport in human erythrocytes with three existing binding sites (I–III) in the membrane.^29,30^ Furthermore, it was shown that **3** (referred to as CB-7 monoacetate in ref (31)) is able to bind site I of the glucose carrier and inhibits glucose transport simultaneously.^30^ Next to the glucose transport system, **2** was described to display inhibitory activity on the human potassium channel hKv1.5,^31^ and it was hypothesized that C-7 acetylation in **3** lowered the cytotoxic activity by reduction of the ion channel activity rather than interfering with the actin cytoskeleton.^32^ In addition, effects herein reported for long-term treatment with **4** raise the pressing question whether pronounced multinucleation accompanied by a nearly unaffected actin network can be associated with hitherto unknown nonactin targets. Finally, we want to draw attention to the intracellular conversion of **5** and **6** to **2** that should be considered in the future regarding the pharmacological application of cytochalasans in general and the interpretation of previous SAR studies on this natural product class.

The current study uncovers new evidence on the SAR of **2** combining an *in silico* docking of **2** onto actin, semisynthesis of selected, delineated derivatives, and cell biological assays. In conclusion, the virtual docking of **2** onto nonpolymerizable actin revealed vital functional groups (i.e., NH group of the isoindolone core (N-2) and the hydroxy group at C-7) involved in stabilizing interactions with the amino acids of the active pocket of actin via hydrogen bonding. Furthermore, methylation at position N-2 and acetylation at the NH in positions N-2 and *O*-acetylation of the hydroxy function at C-7 and C-20 were carried out and afforded derivatives **3**, **4**, **5**, and **6**, respectively. All four compounds displayed a significant reduction of cytotoxicity in murine connective tissue fibroblasts L929, whereas compounds **3** and **4** showed a complete loss of cytotoxicity (IC_50_ > 10 μM in KB3.1 cells.). In line with this, short-term treatments revealed only mild effects on actin arrangements in the U-2OS cell model in the case of **3**–**5**. *In vitro* actin polymerization assays supported that the respective affinity of these derivatives for actin itself is reduced. They also suggested that diminished cell permeability and thus availability to the actin cytoskeleton are not causative of the rather weak activity of modified compounds. In addition, the biological effects of **6**, bearing an acetylation at C-20, were virtually identical to those of **2**, suggesting that the C-20 OH group plays no crucial role in **2**–actin interaction, which is perfectly in line with our initial docking results. Increased actin disruption by **5** upon prolonged treatments are likely caused by enzymatic deacetylation of the derivatives in the cytoplasm, converting them into the highly cytotoxic **2**. Thus, we conclude that the *N*-acetylation of N-2 is a poor candidate for pharmacological exploitation. In contrast, methylation of the same position gave the stable derivative **4**, displaying moderate cytostatic activity in combination with a strongly reduced actin disruption activity, which deserves further scrutiny. Interestingly, acetylation of the hydroxy function at C-7 (**3**) did not induce multinucleation. Instead, it completely blocked proliferation, despite its moderate effects on actin rearrangements. Whereas in principal effects on mitosis and/or cytokinesis can be explained by inhibition of actin dynamics, the specific features of the four derivatives call for searching potential non-actin targets of these processes in the future. This particularly applies to **3**, the effects of which on actin are reversible, while proliferation remains blocked upon washout. Yet, our results confirm the importance of all but the C-20 hydroxy function of the initially identified and chemically modified groups for **2**’s potency in actin polymerization inhibition or F-actin disruption.

## Experimental Section

General experimental procedures and protocols for the syntheses of **3**–**6** are listed in the Supporting Information.

### Docking of Cytochalasin B (**2**) into the Binding Pocket on Monomeric Actin

Structures of cytochalasin B (**2**) and cytochalasin D (**1**) were drawn with the software ChemDraw Professional (PerkinElmer, version 22.0.0.22) and subsequently energy minimized in Chem3D (PerkinElmer, version 22.0.0.22) utilizing the MM2 dynamics calculation (step interval: 2.0 fs, frame interval: 10 fs, heating/cooling rate: 1.000 kcal/atom/ps, target temperature: 300 K).

The energy minimized structures of cytochalasin B (**2**) and cytochalasin D (**1**) were converted into sdf-files and processed with the conformer ensemble generator Conformator^12^ (Conformator is part of the NAOMI ChemBio Suite, which is available free-of-charge at the Center for Bioinformatics at the University of Hamburg (UHH), https://software.zbh.uni-hamburg.de), generating 3772 conformers of cytochalasin D (**1**) and 724 conformers of cytochalasin B (**2**) in an sdf-output (Command: -v 2 -q 2 -n 5000 -o).

The docking of cytochalasin B (**2**) into the binding pocket of cytochalasin D (**1**) onto monomeric actin (PDB: 3EKU)^10^ and its 3D illustration were performed with SeeSAR (version 13.0.5 - Midas, BioSolveIT GmbH, Germany, 2023, http://www.biosolveit.de/SeeSAR)^18^ (parameter: medium clash tolerance and 500 poses per molecule, results were sorted by estimated binding affinities, molecular torsion, inter- and intramolecular clashes).

The docking of cytochalasin D (**1**) perfectly reassembled the cocrystal structure (PDB: 3EKU),^10^ validating our docking procedure. For cytochalasin B (**2**) a docking pose with sufficient binding affinity (low μM) was found in the binding pocket.

### Serial Dilution Assay

Serial dilutions of compounds were prepared as triplicates in sterile U-bottom-shaped 96-well plates (Corning, USA). Mueller-Hinton broth (MHB) medium was used for bacteria, and YMG medium was used for filamentous fungi and yeasts. The selected organisms represent a broad spectrum of pathogens of clinical interest, as well as sensitive indicator strains (Gram-positive bacteria: *Bacillus subtilis* (DSM 10), *Staphylococcus aureus* (DSM 346), *Mycobacterium smegmatis* (ATCC 7000084); Gram-negative bacteria: *Acinetobacter baumannii* (DSM 30008), *Chromobacterium violaceum* (DSM 30191), *Escherichia coli* (DSM 1116), *Pseudomonas aeruginosa* (PA 14); fungi and yeast: *Schizosaccharomyces pombe* (DSM 70572), *Pichia anomala* (DSM 6766), *Mucor hiemalis* (DSM 2656), *Candida albicans* (DSM 1665), *Rhodotorula glutinis* (DSM 10134)).

The compounds were dissolved in MeOH (1 mg/mL), added to the bacterial suspension, and diluted to the final concentrations. The plate was incubated at 37 °C under static conditions. Growth inhibition was assessed at 24 h. MeOH was used as negative control. Kanamycin (1.0 mg/mL; 2 μL [*M*. *smegmatis*]), gentamycin (1.0 mg/mL; 2 μL [*P*. *aeruginosa*]), ciprobay (2.54 mg/mL; 2 μL of [*A*. *baumannii*]), oxytetracycline (1.0 mg/mL; 2 μL of [*C*. *violaceum*, *E*. *coli*, *S*. *aureus*] and 20 μL of [*B*. *subtilis*]), and nystatin (1.0 mg/mL; 20 μL of [*S*. *pombe*, *P*. *anomala*, *M*. *hiemalis*, *C*. *albicans*, *R*. *glutinis*]) were used as positive controls.

### Cytotoxicity Assay

The cytotoxicity assay was implemented in 96-well flat-bottom microtiter plates following a reported procedure.^21,22,33^ The mammalian cell lines L929 (mouse fibroblast) and KB3.1 (human cervix carcinoma) were cultivated at 37 °C and 10% CO_2_ in Dulbecco’s modified minimum essential medium (DMEM, Life Technologies, Carlsbad, CA, USA) supplemented with 10% fetal bovine serum (FBS, Life Technologies). The microtiter plate was filled with 120 μL of suspended cells (50,000/mL), and 60 μL of a serial dilution of the test compound was added. After 5 days of incubation, growth inhibition (IC_50_) was determined by a colorimetric tetrazolium dye MTT assay.^34^ The compounds were dissolved in MeOH (1 mg/mL), MeOH was used as negative control, and epothilone^35^ (1 mg/mL) was used as positive control.

### Cell Culture

The adherent human osteosarcoma cell lines U-2OS [ATCC HTB-96] were cultivated and maintained in DMEM (Life Technologies) containing 10% FBS (Sigma-Aldrich, St. Louis, MO, USA), 1% sodium-pyruvate (Life Technologies), 1% minimum essential medium nonessential amino acids (MEM NEAA, Life Technologies), and 1% l-glutamine (Life Technologies) at 37 °C and 7.5% CO_2_ and split 1:3–1:5 every 2–3 days.

### Actin Disruption Assay

The bioactivity of **2**, **3**, **4**, **5**, and **6** on filamentous actin (F-actin) in U-2OS wild type (wt) cells was investigated in a 1 h end point actin disruption assay, recently described by Kretz et al.^36^ The respective previously determined IC_50_ values in L929 were used to calculate the treatment concentration (1 × IC_50_: low dose; 5 × IC_50_: high dose).^37^ DMSO was used as the vehicle control, corresponding to the highest compound volume used in the assay. Coverslips were coated with 25 μg/mL fibronectin (Roche, Mannheim, Germany) diluted 1:40 in phosphate buffered saline (PBS, pH 7.4, Life Technologies), and cells were seeded at a density of 20,000 cells and allowed to spread overnight. CO_2_-equilibrated culture medium was spiked with the desired compound and DMSO, respectively, in the above-mentioned concentration, and cells were treated with prepared medium for 1 h under cell culture conditions. For long-term experiments, the treatment phase was extended to 24 h. Cells were washed once and fixed afterward using 4% prewarmed paraformaldehyde (PFA) supplied in PBS for 20 min at 37 °C, followed by three additional washing steps with prewarmed PBS buffer. In addition, the reversibility of high dose effects on F-actin network organization was investigated by a washout procedure including three times prewarmed PBS wash steps prior to the addition of fresh medium and a recovery time of 1 h before fixation. In the case of 24 h treatments, the recovery phase was extended to 47 h. Cells were permeabilized with 0.1% Triton X-100 (Bio-Rad Laboratories, Hercules, CA, USA) in PBS buffer for 1 min at room temperature, washed three times with PBS, and stained for F-actin using ATTO-594-coupled phalloidin (ATTO-Tec, Siegen, Germany) diluted in PBS buffer (1:100) for 1 h at room temperature. Coverslips were washed thrice in PBS and mounted in ProLong Diamond antifade mountant (Invitrogen) containing DAPI to probe for nuclear DNA. Cells were recorded using an inverted microscope (Nikon Eclipse Ti2, Tokyo, Japan) equipped with a 60 times Nikon oil immersion objective (Plan Apofluar, 1.4 NA), a pE-4000 (CoolLED, Andover, UK) as light source, and a pco.edge back-illuminated sCMOS camera (Excelitas Technologies, Mississauga, ON, Canada). The microscopy system was operated by and images were recorded with NIS (Nikon) and processed with ImageJ (NIH, Bethesda, MD, USA).

### Cell Proliferation Assay

U-2OS cells were seeded into fibronectin-coated (25 μg/mL) 96-well plate wells at densities of 3700 cells/well and allowed to spread overnight at 37 °C and 7.5% CO_2_. Cells were treated with 5 × IC_50_ concentrations of desired compounds for 24 h, washed twice with prewarmed PBS, and cultured in complete medium for further 47 h. Cells were recorded immediately after compound addition over the course of the experiment using an Incucyte S3 live-cell analysis system (Sartorius, Göttingen, Germany) with a 20 × objective at 37 °C and 5% CO_2_. Three phase contrast images per well and three wells per treatment were captured every hour for 71 h. The quantification of phase object counts was accomplished with the Adherent Cell-by-Cell analysis module of the IncuCyte S3 Live Cell Analysis Software and normalized to the average, respective initial phase contrast count. Graphical presentation of the data and ordinary one-way ANOVA followed by Dunnett’s multiple comparisons test was performed using GraphPad Prism version 8.4.3 for Windows, GraphPad Software (Boston, MA, USA, www.graphpad.com).

### Actin Purification and Labeling for the Pyrene-Actin Assay

Acetone powder and G-actin were prepared as described previously.^24^ Briefly, 1.5 kg of fresh rabbit skeletal muscle was minced with a meat grinder. Subsequently, the meat was stirred for 15 min in 2.5 L of high-salt buffer (0.5 M KCl, 0.1 M K_2_HPO_4_, 2 mM benzamidine, pH 6.4) and then pelleted at 4000*g* for 10 min. The supernatant was discarded, and the pellet was extracted once more with high-salt buffer and then washed twice with ice-cold ddH_2_O adjusting the slurry with 1 M Na_2_CO_3_ to pH 8.3 until a slight swelling of the pellet became visible. The pellet was then resuspended in ice-cold acetone, stirred for 15 min, and then centrifuged for 10 min at 4000*g*. The supernatant was discarded, and the pellet was washed once more with acetone before the pellet was broken up and dried in small pieces on aluminum foil under a fume hood overnight. The dried acetone powder was stored at −80 °C for further use.

For purification of G-actin, 5 g of the acetone powder was extracted five times with 50 mL of G-buffer containing 2 mM Tris-HCl, pH 8.0, 0.2 mM CaCl_2_, 0.5 mM DTT, 0.2 mM Na_2_ATP, and 0.1 mg/mL NaN_3_. After each step, the solution was filtered through a fine gauze, yielding a total volume of 250 mL. Subsequently, the solution was centrifuged for 30 min at 20,000*g* at 4 °C. Actin in the supernatant was then polymerized by addition of 25 × polymerization buffer (250 mM Tris-HCl pH 8.0, 1250 mM KCl, 25 mM Na_2_ATP, 50 mM MgCl_2_ × 6 H_2_O) overnight. Subsequently, KCl was added to a final concentration of 750 mM, followed by centrifugation of the solution at 150,000*g* at 4 °C for 3 h. The F-actin pellet was then homogenized with 25 mL of G-actin buffer using a potter and then dialyzed against G-buffer. After a final centrifugation step at 150,000*g* at 4 °C for 3 h, G-actin was purified by size exclusion chromatography using a HiLoad 26/75 Superdex 200 column. Actin concentrations were determined by measuring the absorbance at 290 nm and using an extinction coefficient of 26,600 M^–1^ cm^–1^.

For pyrene labeling, 10–20 mL of G-actin solution were dialyzed against labeling buffer following the procedure described by Doolittle et al.^38^ A 2-fold molar excess of (*N*-(1-pyrene)iodoacetamide (1 mg) dissolved in approximately 100 μL of anhydrous *N*,*N*-dimethylformamide was added to 10 mL (84.42 μM) of the G-actin solution, and the mixture was then incubated on a rotating wheel for approximately 6 h at 4 °C in the dark. The reaction was stopped by the addition of 1 M DTT, and the solution was then dialyzed against G-buffer overnight. Aliquots of pyrene-labeled actin were snap-frozen in liquid nitrogen and stored at −80 °C for later use.

### Pyrene Assay

Pyrene assays were conducted to assess the inhibitory properties of cytochalasins on actin polymerization under defined conditions *in vitro* using G-actin and actin seeds. The degree of labeling was determined; EC290 nm actin: 26600 1/M × cm; EC342 nm pyrene: 31091 1/M × cm. For generation of actin seeds, 10 μM actin was polymerized in 1 × KMEI (50 mM KCl; 1 mM MgCl_2_; 1 mM EGTA; 10 mM imidazole, pH 7.4) overnight at 4 °C and was then briefly sonicated (Branson Sonifier) prior to experiments. Actin seeds (550 nM) and cytochalasins (2 μM) were prediluted to the desired concentration in 170 μL of polymerization buffer (1.15 × KMEI + 0.05% Antifoam) and transferred into a black 96-well plate (Brand), which was subsequently placed into the Synergy 4 fluorescence microplate reader (BioTek/Agilent, Waldbronn, Germany). To initiate actin assembly, the G-actin mix was added and diluted to a final concentration of 2 μM actin (5% pyrene-labeled) in each well using the automated dispenser of the Synergy 4 plate reader. The fluorescence intensity (extinction, 340/30; emission, 400/30) was measured at 20 s intervals for 30 min. Pyrene-actin fluorescence signals were normalized and plotted over time. Graphical presentation of the data and ordinary one-way ANOVA followed by Dunnett’s multiple comparisons test was performed using GraphPad Prism version 8.4.3 for Windows, GraphPad Software (Boston, MA, USA, www.graphpad.com).

### Microextraction of Compounds

To evaluate possible enzymatic modifications of **3**, **5**, and **6** by, for example, deacetylation events within the cell, microextractions of the cultured medium after 24 h of cell exposure were implemented. Therefore, cells were seeded and treated with 5 × IC_50_ concentrations, as already described. After 24 h, the treatment medium was removed from the cell culture wells and extracted with the doubled volume of ethyl acetate (EtOAc). After evaporation of EtOAc, the dried extracts were diluted in methanol (50 μL) and measured with a high-pressure liquid chromatography system coupled to a diode-array UV/vis detector conjugated with an electrospray ionization mass spectrometer, using an UltiMate 3000 Series uHPLC (Thermo Fisher Scientific) utilizing a C18 Acquity UPLC BEH column (2.1 × 50 mm, 1.7 μm; Waters, Milford, USA) connected to an amaZon speed ESI-Iontrap-MS (Bruker, Billerica, MA, USA). Spectra were analyzed using Bruker Compass DataAnalysis 4.4 SR1.

## Data Availability

The NMR data for **2**, **3**, **4**, **5**, **6**, and **7**–**11** have been deposited at nmrXiv (https://nmrxiv.org) under the DOI: 10.57992/nmrxiv.p61.

## Supporting Information Available

Crystallographic data (PDB)
Experimental protocols for synthesis and biological evaluation, NMR spectra, HRMS spectra, and IR spectra (PDF)

## Supplementary Material

np4c00676_si_001.pdb
