# Supplementary material for: Comprehensive Cell Biological Investigation of Cytochalasin B Derivatives with Distinct Activities on the Actin Network
Source: J Nat Prod. 2024 Oct 11;87(10):2421–31. doi: 10.1021/acs.jnatprod.4c00676 (PMC11519904; doi:10.1021/acs.jnatprod.4c00676)
Supplement: Supplementary file 2 — np4c00676_si_002.pdf [file np4c00676_si_002.pdf]

Comprehensive cell biological investigation of cytochalasin B derivatives with distinct activities on the actin network

Mervic D. Kagho,[‡,†] Katharina Schmidt,[‡,¥] Christopher Lambert,[¥,ǁ,&] Thomas Kaufmann,[§] Lili Jia,[ǁ] Jan Faix,[§] Klemens Rottner,[¥,&] Marc Stadler,[ǁ,£] Theresia Stradal,*[,¥] and Philipp Klahn*[,†]

[†]Department of Chemistry and Molecular Biology, Division of Organic and Medicinal Chemistry, University of Gothenburg, Medicinaregatan 7B, SE-413 90 Göteborg, Sweden. Phone: +46 76 618 3914, Email: philipp.klahn@gu.se.

[¥]Department of Cell Biology, [ǁ]Department of Microbial Drugs, Helmholtz Centre for Infection Research, Inhoffenstrasse 7, D-38124 Braunschweig, Germany. Phone: +49 531 6181 2900, Email: theresia.stradal@helmholtz-hzi.de .

[§]Institute for Biophysical Chemistry, Hannover Medical School, Carl-Neuberg Strasse 1, D-30625 Hannover, Germany.

[&]Division of Molecular Cell Biology, Zoological Institute, Technische Universität Braunschweig, Spielmannstrasse 7, D-38106 Braunschweig, Germany

[£]Institute of Microbiology, Technische Universität Braunschweig, Spielmannstraße 7, D-38106 Braunschweig, Germany

‡These authors contributed equally.

List of contents

Experimental details 2
- General methods 2
- Data availability 6
- Synthesis of the compounds 7
- Biological evaluation of the compounds 16
  - Cell Proliferation Assay 17
  - Microextraction of compounds 20

References 24

Appendix (NMR, IR and Mass Spectra) 25

Experimental details

General methods

Unless otherwise noted, all reagents were purchased from commercial suppliers and used without further purification. (*N,N*-Dimethylformamid (DMF): *Acros Organics*, puriss., extra dry, over molecular sieve (water ≤ 0.005 %), Ethanol (EtOH): *Acros Organics*, puriss., absolut, extra dry (water ≤ 0.005 %), Pyridin (Pyr): *Acros Organics*, puriss., extra dry, over molecular sieve (water ≤ 0.005 %), Dimethylsulfoxid (DMSO): *Acros Organics*, puriss., extra dry, over molecular sieve (water ≤ 0.005 %), Methanol (MeOH): *Acros Organics*, puriss., extra dry (water ≤ 0.005 %)).

Cytochalasin B was purchased in small amounts from *Cfm Oskar Tropitzsch* and additionally isolated from the fermentation of the endophytic fungus *Preussia simillis* G22 (DSM 32328) as described by *Kretz et al.*.[1] G-actin was purified from rabbit skeletal muscle.[2]

Moisture-sensitive reactions were performed under argon atmosphere in dried glassware. Dry dichloromethane, diethyl ether, toluene, and tetrahydrofuran for moisture-sensitive reactions have been taken from a MB-SPS-800 (MBraun) solvent purification system and stored under argon. All solvents used for workup and purification were of HPLC grade. Reactions were monitored by TLC, LCMS, or NMR.

Solutions of compounds in organic solvents were concentrated using rotary evaporators at a water bath temperature of max. 30°C. Solvent residues were removed in a high vacuum at a pressure of approximately 10^{-2} mbar. Unless otherwise noted solvents were degassed either by a continuous argon flow over a minimum of 15 min or using the freeze-pump-thaw (FTP) technique.[3]

Flash chromatography[4] was done using appropriate glass columns filled with silica gel (Merck Millipore, Geduran® Si60, 1.11567.9025, 40–63 µm) or using the Biotage Select® chromatography system with a DAD detector and cartridges packed with silica gel (Merck Millipore, Geduran® Si60, 1.11567.9025, 40-63 µm) using a Cartridger® C-670 from the company Büchi.

Preparative reversed-phase high-pressure liquid chromatography (prep. HPLC RP) was performed on either a Hypersil GOLD C18 RP-column (Part No. 25005-259270), 5 µm, 250 mm × 21.2 mm (10 mL/min) or a Hypersil GOLD C18 RP-column (Part No. 25005-259070A), 5 µm, 250 mm × 10.0 mm (5 mL/min) each equipped with a guard column of the same material using a Thermo Fisher Scientific Dionex Ultimate 3000 HPLC system. Eluents, gradients, and additives are given in parentheses. As eluents, HPLC grade acetonitrile and water (VWR Chemicals, HPLC grade) with or without 0.1 % of TFA (Carl Roth, 6957.1, 99.9 %) or buffer added were used. Appropriate reaction mixtures were filtered through CHROMAFIL®

PET-45/15 MS filters (45 µm) before being injected. The product containing fractions were combined and diluted with dest. H_2O (min. 1:1/solvent:H_2O), frozen and lyophilized using a VaCo2® Freeze dryer from Zirbus (–80°C, 0.05 mbar).

Thin-layer chromatography (TLC) was performed on pre-coated glass plates (Merck TLC Silicagel 60 F_{254}, 1.15341.0001, 2.5 x 7.5 cm) and components were visualized by observation under UV light (λ = 254 nm [UV^{254}] or λ = 366 nm [UV^{366}]) or visible light, treatment of developed plates in an iodine chamber or by treating the plates with TLC staining solutions (for preparation see list below) followed by heating. Eluent or eluent mixtures used are reported in parentheses.

***CAM staining solution* [CAM]:** 1 g Ce(IV)$(SO_4)_2$, 2.5 g $(NH_4)_6Mo_4O_7$ in 100 mL 10% H_2SO_4

***$KMnO_4$ staining solution* [$KMnO_4$]:** 1.5 g $KMnO_4$, 10 g K_2CO_3, and 1.25 mL 10% NaOH in 200 mL H_2O.

Preparative thin-layer chromatography was performed on pre-coated glass plates (Merck TLC Silica gel 60 F_{254}, 1.05715.0001, 20 x2 0 cm, max. 10-15 mg/plate and Analtech Uniplate Silica gel GF Z51305-9, 20 x 20 cm x 2 mm, max. 100-150 mg/plate). Eluent or eluent mixtures used and the number of developments are reported in parentheses. Compounds were visualized by observation under UV light (λ = 254 or 366 nm). Compounds containing silica gel fractions were scratched from the plate with a scalpel, crushed into small pieces, and compounds were eluted with appropriate solvent mixtures.

NMR spectra were recorded on Bruker AV-300, AVIII 400, AVIIIHD 500,an ASCEND 600 FT, Bruker AVNEO 600, and AVIIIHD 800 spectrometers with a cryoprobe system at 293.15 K. ^{1}H NMR spectra were recorded at 300 MHz, 400 MHz, 500 MHz, 600 MHz, and 800 MHz. ^{13}C NMR spectra were recorded at 76 MHz, 100 MHz, 126 MHz, 151 MHz and 201 MHz. Chemical shifts are referenced relative to residual solvent signals. Data are presented as follows: chemical shift, multiplicity (s = singlet, d = doublet, t = triplet, q = quartet, quint = quintet, sext = sextet, sept = septet, m = multiplet etc), and coupling constants in hertz (Hz), followed by the number of hydrogen atoms. For the processing of the raw data, the software MestReNova (Version 14.2.0-26256) from MestreLab Research S.L. was utilized.

Thin-layer chromatography mass spectrometry (TLC-MS) data were recorded on an Advion Expression compact mass spectrometer equipped with an Advion Plate Express automated TLC plate reader.

IR spectra were recorded with a Bruker Tensor 27 IR spectrometer (with the ATR technique).

Low resolution mass spectrometry (LRMS) data were recorded using:

a) a Waters™ LC-MS system consisting of an Acquity Arc HPLC, an ACQ Arc Column Heater/Cooler (0-60°C), 2489 UV/Vis Detector (200-600 nm) and an Acquity QDa Mass Detector (ESI +/-, Quadropole, max.1250 Da, cone voltage: 20.0 V, probe temperature 600°C) equipped with a MS/UV Splitter (10:1) and a column switch with three analytical RP columns (1. Waters™ C18 RP Column XBridge BEH C18 130Å, 2.5 µm, 2.1 x 50 mm with Guard Column XBridge BEH C18 V-Gd Cart 2.5 µ, 2.1 x 5 mm, 2. Waters™ C8 RP Column XBridge PRM BEH 130Å C8 2.5 µm 2.1 x 50 mm with Guard Column XBridge BEH C18 V-Gd Cart 2.5 µm, 2.1 x 5 mm, 3. Waters™ C18 RP Column XBridge BEH C18 130Å, 3.5 µm, 2.1 x 150 mm with Guard Column XBridge BEH C18 V-Gd Cart 2.5 µ, 2.1 x 5 mm); data were analyzed with the Software MassLynx 4.1.

b) a LC-MS system consisting of an Agilent 1100 HPLC system equipped with DAD detector and an Applied Biosystems API 150 EX quadrupole mass detector with electron spray ionization (ESI).

c) a LC-MS system consisting of an Accela HPLC (Thermo Scientific) equipped with an Accela photodiode array (PDA) Detector, Accela autosampler, and Accela 1250 pump which was coupled to an LTQ XL mass spectrometer (Thermo Scientific) for HPLC/HESI-MS analyses. Heated electrospray ionization was used with an enhanced scan range of 120 to 2000 amu. Gradient HPLC solvent programs consisted of LCMS-grade H_2O, CH_3CN, and 2% formic acid in H_2O. An Agilent Zorbax Eclipse Plus C18 (3.5 µm, 2.1 × 150 mm) column was used, which was kept at 30°C. The PDA detector was set to a scanning range from 190 to 600 nm with 1 nm wavelength steps.

High-resolution mass spectrometry (HRMS) data were recorded utilizing:

a) a Finnigan MAT 95 (EI, 70eV) mass spectrometer and a Finnigan MAT 95 XL (ESI) mass spectrometer.

b) a linear iontrap coupled with orbitrap mass analyser LTQ-Orbitrap Velos from Thermo Fisher Scientific (Resolution: 100000 FWHM (at m/z = 400 amu), Scan: 130-2000 amu (resulting in acquisition times of 1.6 sec per cycle)). Electrospraymeasurements were performed in direct infusion mode using a custom made microspray-device mounted on a Proxeon nanospray ion source. The microspray-device allows for the sample infusion through a stainless-steel capillary (90 µm I. D.). Accurate mass measurements in the orbitrap were performed using the lock mass option of the instrument control software using the cation of tetradecyltrimethylammonium bromide (256.29988 amu) as internal mass reference. (Sample concentration: approx. 50 µg/mL. Solvent: MeOH

spiked with 0.1 mg/mL tetradecyltrimethylammonium bromide (unless otherwise stated). Flow: approx. 1 µL/min. Typical spray voltage pos. mode: 2.3 - 2.8 kV. Typical spray voltage neg. mode: 1.7 - 2.5 kV).

c) an Agilent LC-QTOF 6520 with Agilent Infinity 1260 HPLC and Agilent 6520 quadrupole time-of-flight mass analyzer equipped with an ESI or APCI ionization source.

.

Data availability

Nuclear magnetic resonance raw data of all synthesized compounds have been deposited to the NMRXiv (https://nmrxiv.org) with the DOI: 10.57992/nmrxiv.p61.

A PDB of the docking of Cytochalasin B onto non-polymerizable monomeric actin is available as supporting information with this article at the journal's website.

Synthesis of the compounds

Synthesis of 20-*O*-TBS cytochalasin B (7)

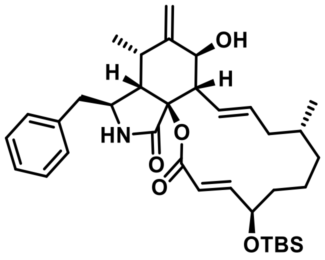

Chemical Formula: $C_{35}H_{51}NO_5Si$
Exact Mass: 593,3537
Molecular Weight: 593,8800

Imidazole (1.1 mg, 17.2 µmol, 1.5 eq) and DMAP (0.3 mg, 2.29 µmol, 0.2 eq) were subsequently added to a solution of cytochalasin B (**2**) (5.5 mg, 11.47 µmol, 1.0 eq) in anhydrous DMF (0.4 mL, 26.2 mM) at 22°C and under argon atmosphere. The resulting mixture was stirred for 15 min before TBSCl (2.4 mg, 17.20 µmol, 1.5 eq) was added and the reaction mixture was heated to 40°C for 24 h. The reaction was monitored by TLC-MS. The solvent was co-evaporated with toluene (2×0.6 mL). The resulting residue was dissolved with CH_2Cl_2 (2 mL) and washed with a saturated solution of NH_4Cl (1 mL). The organic layer was separated, and the aqueous layer was extracted with CH_2Cl_2 (5×1 mL). The combined organic layers were washed with brine (2×1 mL), dried over anhydrous Na_2SO_4, filtrated, and the solvent was removed under reduced pressure. The resulting residue was dissolved with a small amount of CH_2Cl_2 and purified by preparative thin-layer chromatography (EtOAc:hexanes/0:1→5:5) yielding 20-*O*-TBS cytochalasin B (**7**) (5.0 mg, 8.42 µmol, 80 %) as a white amorph solid.

TLC (hexanes:EtOAc/1:1), R_f: 0.46 [UV^{254}, CAM]. **IR** (ATR) [cm^{-1}]: 3242, 2930, 2859, 2323, 1710, 1458, 1259, 1098, 1039, 966, 904, 834, 777, 698, 690, 670, 585. **TLC-MS** (ESI) [m/z]: 616.3 for $[C_{35}H_{51}NNaO_5Si]^+$. **HRMS** (ESI) [m/z]: calculated for $[C_{35}H_{51}NNaO_5Si]^+$, 616.34287; found 616.34297. **^{1}H-NMR** (500 MHz, $CDCl_3$) δ [ppm]: 7.32 (t, *J* = 7.4 Hz, 2H), 7.26–7.22 (m, 1H), 7.16 (d, *J* = 6.9 Hz, 2H), 7.01 (dd, *J* = 15.4, 4.2 Hz, 1H), 5.95 (ddd, *J* = 15.0, 9.8, 1.8 Hz, 1H), 5.87 (dd, *J* = 15.5, 1.8 Hz, 1H), 5.57 (d, *J* = 5.8 Hz, 1H), 5.44 (ddd, *J* = 14.8, 11.0, 3.7 Hz, 1H), 5.39 (s, 1H), 5.17 (s, 1H), 4.49–4.46 (m, 1H), 3.87 (dd, *J* = 11.1, 1.4 Hz, 1H), 3.40 (dd, *J* = 12.8, 8.2 Hz, 2H), 3.30 (dt, *J* = 9.7, 3.7 Hz, 1H), 2.85 (dt, *J* = 6.8, 5.0 Hz, 2H), 2.76 (dd, *J* = 13.5, 9.6 Hz, 1H), 2.13–2.08 (m, 1H), 1.86 (ddd, *J* = 13.9, 8.1, 6.1 Hz, 1H), 1.77–1.69 (m, 3H), 1.69–1.62 (m, 1H), 1.44 (dddd, *J* = 35.6, 13.2, 8.8, 4.1 Hz, 1H), 1.26 (s, 2H), 1.11 (d, *J* = 6.8 Hz, 3H), 0.92 (s, 9H), 0.88 (d, *J* = 6.6 Hz, 3H), 0.07 (s, 3H), 0.06 (s, 3H). **^{13}C-NMR** (126 MHz, $CDCl_3$) δ [ppm]: 171.28, 165.00, 153.47, 148.42, 138.23, 137.33, 129.16, 128.94,

127.06, 126.19, 118.69, 114.61, 83.01, 77.20, 71.03, 69.24, 53.47, 49.31, 48.71, 44.48, 41.87, 35.62, 35.44, 33.19, 31.89, 25.82, 20.40, 19.93, 18.22, 14.14, –4.88, –4.89.

Synthesis of 7-*O*-acetyl-20-*O*-TBS cytochalasin B (8)

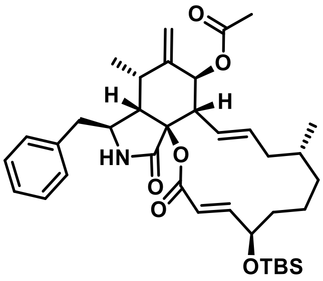

Chemical Formula: $C_{37}H_{53}NO_6Si$
Exact Mass: 635,3642
Molecular Weight: 635,9170

20-*O*-TBS cytochalasin B (**7**) (5.0 mg, 8.42 µmol, 1.0 eq) was dissolved in anhydrous CH_2Cl_2 (0.3 mL, 0.03 M) under argon atmosphere at 23°C. DMAP (1.0 mg, 8.42 µmol, 1.0 eq) and NEt_3 (0.9 mg, 1.2 µL, 8.42 µmol, 1.0 eq) were added and the reaction mixture was stirred at 23°C for 20 min before acetic acid anhydride (2.6 mg, 2.4 µL, 25.26 µmol, 1.0 eq) was added dropwise and the mixture was stirred for additional 1 h at 23°C. The reaction was monitored by TLC-MS. The solvent was removed under reduced pressure. The resulting residue was purified by preparative thin-layer chromatography (EtOAc:hexanes/1:1) yielding 7-*O*-acetyl-20-*O*-TBS cytochalasin B (**8**) (4.9 mg, 7.71 µmol, 92 %) as a colorless oil.

TLC (hexanes:EtOAc/1:1), R_f: 0.68 [UV^{254}, CAM]. **IR** (ATR) [cm^{-1}]: 2953, 2927, 2857, 1740, 1710, 1456, 1372, 1232, 1100, 1043, 1024, 973, 836, 775, 701. **TLC-MS** (ESI) [m/z]: 658.4 for $[C_{37}H_{53}NNaO_6Si]^+$. **HRMS** (ESI) [m/z]: calculated for $[C_{37}H_{54}NO_6Si]^+$, 636.37149; found 636.36827. **^{1}H-NMR** (600 MHz, $CDCl_3$) δ [ppm]: 7.30 (t, *J* = 7.4 Hz, 2H), 7.25 – 7.21 (m, 1H), 7.15 (d, *J* = 6.8 Hz, 2H), 6.98 (dd, *J* = 15.4, 4.1 Hz, 1H), 5.91 (s, 1H), 5.85 (dd, *J* = 15.4, 1.9 Hz, 1H), 5.81 (ddd, *J* = 15.0, 9.8, 1.9 Hz, 1H), 5.31 – 5.22 (m, 3H), 5.11 (s, 1H), 4.45 (h, *J* = 2.4 Hz, 1H), 3.61 (dd, *J* = 11.3, 9.9 Hz, 1H), 3.46 – 3.40 (m, 1H), 3.32 (dt, *J* = 7.9, 4.0 Hz, 1H), 2.87 (dd, *J* = 4.9, 3.0 Hz, 1H), 2.83 (dd, *J* = 13.5, 4.6 Hz, 1H), 2.76 (dd, *J* = 13.4, 9.0 Hz, 1H), 2.01 – 1.97 (m, 1H), 1.96 (s, 3H), 1.87 – 1.81 (m, 1H), 1.70 – 1.64 (m, 1H), 1.63 – 1.58 (m, 1H), 1.44 (ddd, *J* = 13.7, 10.2, 3.0 Hz, 1H), 1.31 – 1.28 (m, 1H), 1.26 – 1.25 (m, 1H), 1.06 (d, *J* = 6.7 Hz, 3H), 0.92 (s, 9H), 0.88 (t, *J* = 7.2 Hz, 2H), 0.84 (d, *J* = 6.6 Hz, 3H), 0.06 (d, *J* = 1.3 Hz, 6H). **^{13}C-NMR** (151 MHz, $CDCl_3$) δ [ppm]: 171.09, 170.09, 165.02, 153.61, 146.05, 137.09, 136.05, 129.23, 128.89, 127.03, 125.69, 118.52, 115.53, 83.01, 70.92, 70.90, 53.43, 48.85, 45.71, 44.34, 41.69, 35.50, 35.38, 33.23, 32.15, 29.68, 25.80, 21.09, 20.35, 19.92, 18.20, 13.96.

Synthesis of 7,20-*O,O'*-diacetyl cytochalasin B (9)

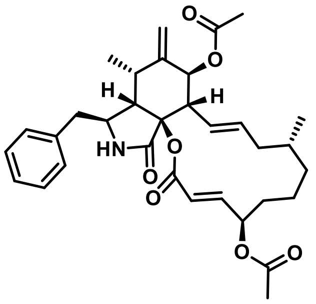

Chemical Formula: $C_{33}H_{41}NO_7$
Exact Mass: 563,2883
Molecular Weight: 563,6910

Cytochalasin B (**2**) (4.0 mg, 8.34 µmol, 1.0 eq) was dissolved in anhydrous CH_2Cl_2 (0.3 mL, 0.028 M). DMAP (5.1 mg, 41.70 µmol, 5.0 eq) and NEt_3 (4.2 mg, 5.8 µL, 41.5 µmol, 5.0 eq) were added subsequently to the resulting solution at 23°C. The reaction mixture was stirred for 20 min, before acetyl chloride (3.2 mg, 3.0 µL, 40.70 µmol, 5.0 eq) was added and the mixture was stirred for additional 4 h at 23°C. The reaction was monitored by TLC-MS. The solvent and excess of acetyl chloride were removed under reduced pressure. The resulting crude product was purified by preparative thin-layer chromatography (EtOAc:hexanes/1:1) yielding 7,20-*O,O'*-diacetyl cytochalasin B (**9**) (4.7 mg, 8.34 µmol, 98 %) as a white amorphous solid.

TLC (hexanes:EtOAc/1:1), R_f: 0.67 [UV^{254}, CAM]. **TLC-MS** (ESI) [m/z]: 586.3 for $[C_{33}H_{41}NNaO_7]^+$. **^{1}H-NMR** (600 MHz, $CDCl_3$) δ [ppm]: 7.29 (t, *J* = 7.4 Hz, 2H), 7.24 – 7.21 (m, 1H), 7.14 (d, *J* = 6.7 Hz, 2H), 6.92 (dd, *J* = 15.8, 5.1 Hz, 1H), 5.98 (s, 1H), 5.83 (ddd, *J* = 15.0, 9.8, 1.9 Hz, 1H), 5.78 (dd, *J* = 15.8, 1.7 Hz, 1H), 5.47 (tdd, *J* = 5.2, 3.2, 1.7 Hz, 1H), 5.31 – 5.22 (m, 3H), 5.10 (s, 1H), 3.53 (dd, *J* = 11.5, 9.8 Hz, 1H), 3.38 – 3.29 (m, 2H), 2.83 – 2.79 (m, 3H), 2.10 (s, 3H), 2.03 – 1.97 (m, 1H), 1.95 (s, 3H), 1.75 – 1.63 (m, 2H), 1.58 – 1.46 (m, 2H), 1.30 – 1.19 (m, 3H), 1.01 (d, *J* = 6.7 Hz, 3H), 0.85 (d, *J* = 6.5 Hz, 3H), 0.65 (dtd, *J* = 13.4, 10.9, 5.1 Hz, 1H). **^{13}C-NMR** (151 MHz, $CDCl_3$) δ [ppm]: 170.77, 169.99, 169.77, 164.05, 147.79, 145.82, 137.00, 135.80, 129.23, 128.84, 127.04, 125.94, 119.94, 115.53, 83.03, 72.41, 70.76, 53.35, 48.62, 46.06, 43.98, 41.44, 34.88, 33.17, 32.09, 31.90, 29.63, 21.04, 20.92, 20.23, 13.92.

Synthesis of 7-*O*-acetyl cytochalasin B (3)

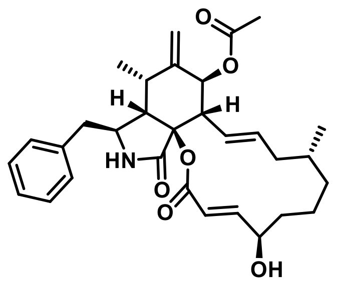

Chemical Formula: $C_{31}H_{39}NO_6$
Exact Mass: 521,2777
Molecular Weight: 521,6540

Method A: 7,20-*O,O'*-diacetyl cytochalasin B (**9**) (4.0 mg, 7.10 µmol, 1.0 eq) was dissolved in anhydrous MeOH (0.3 mL, 0.028 M) under argon atmosphere and at 23°C. K_2CO_3 (1.2 mg, 8.52 µmol, 1.2 eq) was added and the reaction mixture was stirred for 40 min. The reaction was monitored by TLC-MS. A saturated solution of NH_4Cl (0.6 mL) was added and the resulting mixture was extracted with CH_2Cl_2 (7×1 mL). The combined organic layers were washed with brine (2×1 mL), dried over anhydrous Na_2SO_4, filtrated, and the solvent was evaporated under reduced pressure. The resulting residue was purified by preparative thin-layer chromatography (EtOAc:hexanes/0:1→1:1) yielding 7-*O*-acetylcytochalasin B (**3**) (3.6 mg, 6.90 µmol, 97 %) as a white amorphous solid.

Method B: 7-*O*-acetyl-20-*O*-TBS cytochalasin B (**8**) (4.0 mg, 6.29 µmol, 1.0 eq) was dissolved in anhydrous THF (0.3 mL, 0.021 M) at 0°C. Aqueous HF (2.6 µL, 75.48 µmol, 48 wt% in H_2O, 12.0 eq) and TBAF (12.6 µL, 12.58 µmol, 1.0 M in THF, 2.0 eq) were added subsequently at 0°C. The reaction mixture was warmed to 23°C and stirred for 18 h. The reaction was monitored by TLC-MS. The mixture was diluted with EtOAc (1 mL), washed with water (3×0.7 mL) and brine (0.7 mL), dried over Na_2SO_4, filtrated, and the solvent was evaporated under reduced pressure. The resulting residue was purified by preparative thin-layer chromatography (EtOAc:hexanes/1:1) yielding 7-*O*-acetyl cytochalasin B (**3**) (2.3 mg, 4.41 µmol, 70%) as a white amorphous solid.

TLC (hexanes:EtOAc/1:1), R_f: 0.50 [UV^{254}, CAM]. **IR** (ATR) [cm^{-1}]: 3325, 2922, 2857, 1710, 1454, 1372, 1236, 1163, 1092, 1025, 978, 911, 805, 760, 703, 557, 547. **TLC-MS** (ESI) [m/z]: 544.3 for $[C_{31}H_{39}NNaO_6]^+$. **HRMS** (ESI) [m/z]: calculated for $[C_{31}H_{39}NNaO_6]^+$, 544.26696; found 544.26715. **^{1}H-NMR** (600 MHz, $CDCl_3$) δ [ppm]: 7.31 (t, *J* = 7.5 Hz, 2H), 7.23 (td, *J* = 6.7, 3.3 Hz, 1H), 7.15 (d, *J* = 7.9 Hz, 2H), 7.06 – 7.00 (m, 1H), 5.88 (dd, *J* = 15.7, 2.7 Hz, 1H), 5.80 (dd, *J* = 15.1, 9.7 Hz, 1H), 5.30 – 5.26 (m, 2H), 5.26 (s, 1H), 5.11 (s, 1H), 4.51 – 4.46 (m, 1H), 3.53 (dd, *J* = 11.5, 9.7 Hz, 1H), 3.44 – 3.39 (m, 1H), 3.36 (t, *J* = 5.8 Hz, 1H), 3.31 (td, *J* = 7.0, 3.2 Hz, 1H), 2.85 – 2.83 (m, 1H), 2.81 (d, *J* = 6.9 Hz, 2H), 2.01 (dt, *J* = 13.4, 2.9 Hz, 1H), 1.95 (s, 3H), 1.94 – 1.87 (m, 1H), 1.74 – 1.63 (m, 2H), 1.62 – 1.57 (m, 1H), 1.46 (dh, *J* = 12.2, 5.4

Hz, 1H), 1.34 – 1.20 (m, 2H), 1.03 (d, *J* = 4.3 Hz, 3H), 0.85 (d, *J* = 6.8 Hz, 3H), 0.71 – 0.58 (m, 1H). **^{13}C-NMR** (151 MHz, $CDCl_3$) δ [ppm]: 170.91, 170.09, 164.56, 152.31, 145.94, 137.16, 135.97, 129.19, 128.89, 127.02, 125.81, 119.02, 115.47, 82.91, 70.69, 53.42, 48.84, 46.22, 44.16, 41.51, 35.00, 34.81, 33.04, 32.13, 29.65, 26.46, 21.07, 20.31, 14.03.

Synthesis of *N*-methyl cytochalasin B (4)

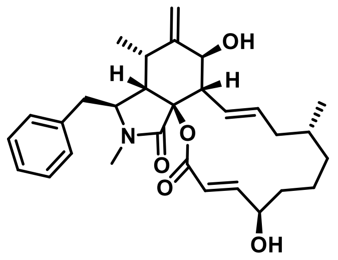

Chemical Formula: $C_{30}H_{39}NO_5$
Exact Mass: 493,2828
Molecular Weight: 493,6440

Cytochalasin B (**2**) (5.0 mg, 10.43 µmol, 1.0 eq) was dissolved in anhydrous THF (0.4 mL, 0.026 M) under argon atmosphere at 0°C and NaH (2.0 mg, 52.13 mmol, 5.0 eq, 60 % dispersion in paraffin oil) was added. The resulting mixture was stirred at 0°C for 30 min before methyliodide (7.4 mg, 3.2 µL, 52.13 µmol, 5.0 eq) was added dropwise. The reaction mixture was warmed up to 22°C and stirred for 2 h. The reaction was monitored by TLC-MS. A saturated solution of NH_4Cl (0.6 mL) was added and the resulting mixture was extracted with CH_2Cl_2 (7×1 mL). The combined organic layers were washed with brine (3×1 mL), dried over Na_2SO_4, filtrated, and the solvent was evaporated under reduced pressure. The resulting residue was purified by preparative TLC using (EtOAc:hexanes/1:1) yielding *N*-methyl cytochalasin B (**4**) (4.1 mg, 8.31 µmol, 80 %) as a white amorphous solid.

TLC (hexanes:EtOAc/1:1), R_f: 0.26 [UV^{254}, CAM]. **IR** (ATR) [cm^{-1}]: 3424, 2921, 2860, 1693, 1453, 1408, 1372, 1258, 1086, 1027, 974, 907, 754, 698, 691, 571. **TLC-MS** (ESI) [m/z]: 516.3 $[C_{30}H_{39}NNaO_5]^+$. **HRMS** (ESI) [m/z]: calculated for $[C_{30}H_{39}NNaO_5]^+$, 516.27204; found 516.27202. **^{1}H-NMR** (500 MHz, $CDCl_3$) δ [ppm]: 7.28–7.25 (m, 2H), 7.20–7.16 (m, 1H), 7.09–7.01 (m, 2H), 6.91 (dd, *J* = 15.7, 3.8 Hz, 1H), 5.38 (ddd, *J* = 15.0, 11.0, 3.8 Hz, 1H), 5.23 (d, *J* = 1.3 Hz, 1H), 5.04–5.01 (m, 1H), 4.53 (dq, *J* = 5.4, 3.2, 2.7 Hz, 1H), 3.80 (d, *J* = 10.2 Hz, 1H), 3.45 (t, *J* = 10.0 Hz, 1H), 3.24–3.18 (m, 2H), 2.96–2.88 (m, 1H), 2.82 (s, 3H), 2.74 (dd, *J* = 13.6, 7.5 Hz, 1H), 2.08 (ddt, *J* = 13.2, 4.1, 2.2 Hz, 1H), 1.93 (ddd, *J* = 14.0, 8.1, 5.5 Hz, 1H), 1.71 (dt, *J* = 13.4, 11.1 Hz, 4H), 1.64 (dd, *J* = 5.0, 2.0 Hz, 1H), 1.62 (d, *J* = 3.3 Hz, 1H), 1.60–1.58 (m, 2H), 1.52–1.45 (m, 4H), 0.87 (d, *J* = 6.6 Hz, 3H), 0.71 (d, *J* = 6.7 Hz, 3H). **^{13}C-NMR** (126 MHz, $CDCl_3$) δ [ppm]: 169.42, 164.48, 152.09, 148.47, 137.25, 136.41, 129.78, 128.65, 127.17, 126.90, 118.67, 114.06, 83.56, 70.56, 70.27, 59.59, 48.43, 45.20, 41.93, 40.09, 35.20, 34.75, 33.07, 31.29, 28.52, 20.35, 20.04, 13.14.

Synthesis of 7,20- *O,O'*-di-TBS cytochalasin B (10)

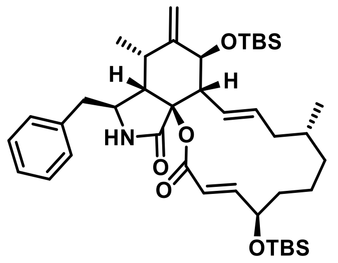

Chemical Formula: $C_{41}H_{65}NO_5Si_2$
Exact Mass: 707,4401
Molecular Weight: 708,1430

Cytochalasin B (**2**) (8.0 mg, 16.68 µmol, 1.0 eq) was dissolved in anhydrous DMF (0.5 mL, 0.031 M) under argon atmosphere at 24°C. Imidazole (10.4 mg, 166.81 µmol, 10.0 eq) and DMAP (1.9 mg, 0.0153 mmol, 1.0 eq) were subsequently added and the resulting mixture was stirred for 15 min at 0°C before TBSCl (23.1 mg, 166.81 µmol, 10.0 eq) was added in two portions over a period of 30 min. The reaction mixture was heated to 40°C for 2 h. The reaction was monitored by TLC-MS. The solvent was co-evaporated with toluene (2×0.6 mL). The resulting residue was dissolved with CH_2Cl_2 (3 mL) and washed with a saturated solution of NH_4Cl (1 mL). The organic layer was separated, and the aqueous layer was extracted with CH_2Cl_2 (5×1 mL). The combined organic layers were washed with brine (2×1 mL), dried over Na_2SO_4, filtrated, and the solvent was evaporated under reduced pressure. The resulting residue was purified by preparative thin-layer chromatography (EtOAc:hexanes/0:1→1:9→2:8) yielding 7,20-*O,O'*-di-TBS cytochalasin B **10** (9.2 mg, 12.99 µmol, 85 %) as a colorless oil.

TLC (hexanes:EtOAc/4:1), R_f: 0.40 [UV^{254}, CAM]. **IR** (ATR) [cm^{-1}]: 3225, 2935, 2859, 1712, 1462, 1364, 1255, 1094, 967, 835, 777, 690. **TLC-MS** (ESI) [m/z]: 730.4 for $[C_{41}H_{65}NNaO_5Si_2]^+$. **HRMS** (ESI) [m/z]: calculated for $[C_{41}H_{65}NNaO_5Si_2]^+$, 730.42935; found 730.42948. **^{1}H-NMR** (500 MHz, $CDCl_3$) δ [ppm]: 7.31 (t, *J* = 7.4 Hz, 2H), 7.25–7.21 (m, 1H), 7.15 (d, *J* = 6.8 Hz, 2H), 5.88 (ddd, *J* = 15.0, 9.8, 1.6 Hz, 1H), 5.87 (dd, *J* = 15.5, 1.9 Hz, 1H), 5.56 (s, 1H), 5.24 (ddd, *J* = 14.9, 11.0, 3.7 Hz, 1H), 5.10 (s, 1H), 4.99 (s, 1H), 4.51–4.42 (m, 1H), 3.94 (d, *J* = 10.0 Hz, 1H), 3.52–3.44 (m, 1H), 3.37 (t, *J* = 6.3 Hz, 1H), 3.31 (dq, *J* = 9.6, 3.8, 3.0 Hz, 1H), 2.92 (dd, *J* = 5.3, 2.6 Hz, 1H), 2.84–2.64 (m, 2H), 2.04–1.97 (m, 1H), 1.92–1.81 (m, 1H), 1.77–1.58 (m, 4H), 1.04 (d, *J* = 6.7 Hz, 3H), 1.00–0.95 (m, 1H), 0.92 (s, 9H), 0.91–0.84 (m, 4H), 0.81 (s, 9H), 0.64–0.50 (m, 1H), 0.06 (d, *J* = 1.2 Hz, 6H), –0.02 (d, *J* = 14.2 Hz, 6H). **^{13}C-NMR** (126 MHz, $CDCl_3$) δ [ppm]: 171.85, 165.01, 153.18, 149.95, 137.47, 135.67, 129.20, 128.87, 127.70, 126.95, 118.83, 112.94, 83.61, 72.05, 70.88, 53.44, 53.40, 49.13, 48.71, 44.38, 42.13, 35.56, 35.42, 33.23, 31.58, 25.83, 25.64, 22.64, 20.40, 19.96, 18.21, 18.06, 14.19, 13.89, –3.76, –3.80, –4.88, –4.91.

Synthesis of *N*-acetyl-7,20-*O,O'*-di-TBS cytochalasin B (11)

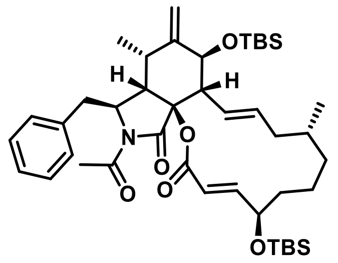

Chemical Formula: $C_{43}H_{67}NO_6Si_2$
Exact Mass: 749,4507
Molecular Weight: 750,1800

7,20-*O,O'*-di-TBS cytochalasin B (**10**) (5.7 mg, 8.05 µmol, 1.0 eq) was dissolved in anhydrous THF (0.5 mL, 0.016 M) under argon atmosphere at 0°C. NaH (1.0 mg, 25.00 µmol, 3.0 eq, 60% dispersion in paraffin oil) was added and the resulting mixture was stirred at 0°C for 30 min before acetyl chloride (3.1 mg, 2.9 µL, 38.99 µmol, 5.0 eq) was added dropwise and the reaction mixture was stirred for additional 1.5 h at 0°C. The reaction was monitored by TLC-MS. The solvent and excess of acetyl chloride were removed under reduced pressure. The resulting residue was purified by preparative thin-layer chromatography (EtOAc:hexanes/1:4) yielding *N*-acetyl-7,20-*O,O'*-di-TBS cytochalasin B (**11**) (5.7 mg, 7.6 µmol, 95 %) as a colorless oil.

TLC (hexanes:EtOAc/4:1), R_f: 0.45 [UV^{254}, CAM]. **IR** (ATR) [cm^{-1}]: 2924, 2857, 1714, 1460, 1372, 1251, 1093, 967, 837, 777. **TLC-MS** (ESI) [m/z]: 772.4 $[C_{43}H_{67}NNaO_6Si_2]^+$. **HRMS** (ESI) [m/z]: calculated for $[C_{43}H_{67}NNaO_6Si_2]^+$, 772.44046; found 772.44008. **^{1}H-NMR** (500 MHz, $CDCl_3$) δ [ppm]: 7.28 (t, *J* = 7.5 Hz, 2H), 7.21–7.17 (m, 1H), 7.15–7.09 (m, 2H), 6.90 (dd, *J* = 15.4, 3.2 Hz, 1H), 5.92 (dd, *J* = 15.4, 2.0 Hz, 1H), 5.83 (ddd, *J* = 15.1, 10.0, 1.8 Hz, 1H), 5.32–5.22 (m, 1H), 4.90 (s, 1H), 4.84 (s, 1H), 4.52 (dq, *J* = 5.2, 2.6 Hz, 1H), 4.19 (dd, *J* = 10.0, 3.4 Hz, 1H), 3.88 (d, *J* = 8.5 Hz, 1H), 3.55 (dd, *J* = 9.9, 8.5 Hz, 1H), 3.18 (dd, *J* = 12.8, 3.4 Hz, 1H), 3.09 (d, *J* = 2.7 Hz, 2H), 2.60 (dd, *J* = 12.8, 10.1 Hz, 1H), 2.49 (s, 3H), 2.01 (ddd, *J* = 11.2, 4.1, 2.1 Hz, 1H), 1.87 (ddd, *J* = 13.8, 7.9, 5.4 Hz, 1H), 1.62 (qd, *J* = 14.3, 13.6, 10.6 Hz, 5H), 1.49–1.40 (m, 2H), 0.94 (s, 9H), 0.87 (dd, *J* = 6.8, 5.7 Hz, 4H), 0.77 (s, 9H), 0.66–0.53 (m, 2H), 0.08 (d, *J* = 4.6 Hz, 6H), –0.05 (d, *J* = 7.8 Hz, 6H). **^{13}C-NMR** (126 MHz, $CDCl_3$) δ [ppm]: 171.70, 170.71, 164.42, 153.66, 149.60, 136.85, 136.36, 129.75, 128.62, 127.33, 126.71, 118.53, 112.48, 85.96, 74.08, 70.48, 56.15, 49.30, 43.16, 42.27, 40.28, 35.76, 35.24, 33.22, 31.92, 30.79, 29.69, 29.36, 25.84, 25.67, 25.60, 22.69, 20.43, 19.76, 18.24, 17.98, 14.11, 12.75, –4.02, –4.13, –4.91.

Synthesis of *N*-acetyl cytochalasin B (5)

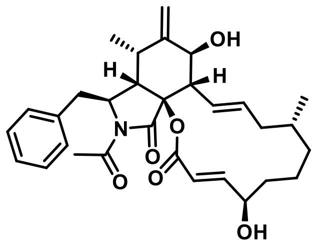

Chemical Formula: $C_{31}H_{39}NO_6$
Exact Mass: 521,2777
Molecular Weight: 521,6540

TBAF (15.4 µL, 15.33 µmol, 2.3 eq, 1.0 M in THF) was added to a solution of compound **11** (5.0 mg, 6.67 µmol, 1.0 eq) in anhydrous THF (0.3 mL, 0.022 M) at 0°C and under argon atmosphere. The reaction mixture was warmed to 23°C and stirred for 16 h. The reaction was monitored by TLC-MS. The solvent was removed under reduced pressure and the resulting residue was purified by preparative thin-layer chromatography (EtOAc:hexanes/1:1) yielding *N*-acetyl cytochalasin B (**5**) (1.7 mg, 3.26 µmol, 50 %) as a white amorphous solid along with cytochalasin B (**2**) (0.2 mg, 0.42 µmol, 6%).

TLC (hexanes:EtOAc/1:1), R_f: 0.36 [UV^{254}, CAM]. **IR** (ATR) [cm^{-1}]: 3419, 2923, 2857, 1709, 1455, 1374, 1256, 1086, 1028, 983, 915, 738, 698, 690, 611. **TLC-MS** (ESI) [m/z]: 544.3 $[C_{31}H_{39}NNaO_6]^+$. **HRMS** (ESI) [m/z]: calculated for $[C_{31}H_{39}NNaO_6]^+$, 544.26696; found 544.26707, err [ppm] 0.20. **^{1}H-NMR** (600 MHz, $CDCl_3$) δ [ppm]: 7.28 (t, *J* = 7.6 Hz, 2H), 7.20 (t, *J* = 7.4 Hz, 1H), 7.12 (d, *J* = 6.9 Hz, 2H), 6.95 (dd, *J* = 15.8, 4.2 Hz, 1H), 5.96 (dd, *J* = 15.7, 1.9 Hz, 1H), 5.87 (ddd, *J* = 15.1, 9.8, 1.9 Hz, 1H), 5.45 (ddd, *J* = 14.9, 10.9, 3.8 Hz, 1H), 5.19 (s, 1H), 5.01 (s, 1H), 4.62 – 4.54 (m, 1H), 4.11 (ddd, *J* = 9.8, 3.4, 1.7 Hz, 1H), 3.81 (d, *J* = 10.3 Hz, 1H), 3.50 (t, *J* = 9.9 Hz, 1H), 3.18 (dd, *J* = 12.9, 3.4 Hz, 1H), 3.09 (t, *J* = 6.3 Hz, 1H), 2.94 (dd, *J* = 5.8, 1.7 Hz, 1H), 2.69 (dd, *J* = 12.9, 9.7 Hz, 1H), 2.50 (s, 3H), 2.13 (ddt, *J* = 13.4, 4.2, 2.3 Hz, 1H), 1.96 (ddd, *J* = 14.2, 8.2, 5.5 Hz, 1H), 1.79 –1.70 (m, 1H), 1.66 –1.58 (m, 2H), 1.26 (dd, *J* = 5.3, 2.5 Hz, 4H), 0.89 (d, *J* = 8.3 Hz, 3H), 0.57 (d, *J* = 6.7 Hz, 3H). **^{13}C-NMR** (151 MHz, $CDCl_3$) δ [ppm]: 171.14, 170.45, 164.06, 152.65, 147.83, 138.14, 136.53, 129.94, 128.64, 126.84, 126.16, 118.54, 114.98, 84.87, 71.04, 70.33, 56.07, 48.83, 43.41, 41.96, 40.24, 35.24, 34.74, 33.00, 31.10, 25.57, 20.37, 20.02, 12.77.

Synthesis of 20-*O*-acetyl cytochalasin B (6)

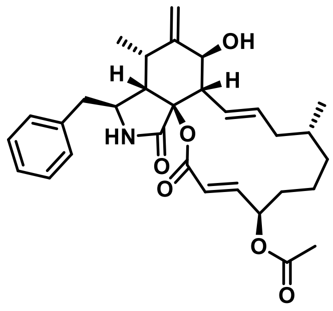

Chemical Formula: $C_{31}H_{39}NO_6$
Exact Mass: 521,2777
Molecular Weight: 521,6540

Cytochalasin B (**2**) (5.0 mg, 10.43 µmol, 1.0 eq) was dissolved with anhydrous CH_2Cl_2 (0.4 mL, 0.026 M) under argon atmosphere at 23°C, NEt_3 (1.3 mg, 1.7 µL, 12.51 µmol, 1.2 eq) was added and the reaction mixture was stirred at 23°C for 20 min before acetic anhydride (1.3 mg, 1.2 µL, 12.51 µmol, 1.2 eq) was added dropwise and the mixture was stirred for additional 24 h at 23°C. The reaction was monitored by TLC-MS. The solvent and excess of acetyl chloride were removed under reduced pressure. The resulting residue was purified by preparative thin-layer chromatography (EtOAc:hexanes/1:1) yielding 20-*O*-acetyl cytochalasin B (**6**) (3.7 mg, 7.09 µmol, 70 %) as a white amorphous solid.

TLC (hexanes:EtOAc/1:1), R_f: 0.47 [UV^{254}, CAM]. **IR** (ATR) [cm^{-1}]: 3255, 2921, 2861, 1711, 1446, 1372, 1238, 1089, 1024, 978, 911, 757, 697, 690, 673. **TLC-MS** (ESI) [m/z]: 544.3 $[C_{31}H_{39}NNaO_6]^+$. **HRMS** (ESI) [m/z]: calculated for $[C_{31}H_{39}NNaO_6]^+$, 544.26696; found 544.26721, err [ppm] 0.46. **^{1}H-NMR** (800 MHz, $CDCl_3$) δ [ppm]: 7.31 (t, *J* = 7.6 Hz, 2H), 7.25 (t, *J* = 6.9 Hz, 1H), 7.16 (d, *J* = 6.5 Hz, 2H), 6.96 (dd, *J* = 16.6, 5.4 Hz, 1H), 5.96 (dd, *J* = 15.2, 9.8 Hz, 1H), 5.81 (dd, *J* = 15.8, 1.6 Hz, 1H), 5.64 (s, 1H), 5.52 – 5.49 (m, 1H), 5.45 (ddd, *J* = 14.8, 11.0, 3.6 Hz, 1H), 5.39 (s, 1H), 5.17 (s, 1H), 3.88 (d, *J* = 11.1 Hz, 1H), 3.42 – 3.24 (m, 3H), 2.84 – 2.81 (m, 2H), 2.79 (dd, *J* = 4.9, 3.2 Hz, 1H), 2.13 (dq, *J* = 13.2, 2.6 Hz, 1H), 2.11 (s, 3H), 2.02 (ddd, *J* = 14.2, 8.4, 5.7 Hz, 1H), 1.76 (dt, *J* = 13.7, 11.2 Hz, 1H), 1.74 – 1.69 (m, 1H), 1.59 (ddd, *J* = 14.8, 10.3, 3.4 Hz, 1H), 1.56 – 1.51 (m, 1H), 1.32 – 1.26 (m, 2H), 1.25 (s, 1H), 1.09 (d, *J* = 6.7 Hz, 3H), 0.89 (d, *J* = 6.6 Hz, 3H), 0.70 (dtd, *J* = 13.4, 10.9, 5.1 Hz, 1H). **^{13}C-NMR** (201 MHz, $CDCl_3$) δ [ppm]: 171.06, 169.90, 164.07, 148.26, 147.70, 138.05, 137.28, 129.17, 128.95, 127.10, 126.39, 120.27, 114.71, 83.02, 72.58, 69.02, 53.50, 49.18, 49.16, 44.16, 41.63, 34.98, 33.17, 32.07, 31.87, 21.10, 20.97, 20.32, 14.18.

Biological evaluation of the compounds

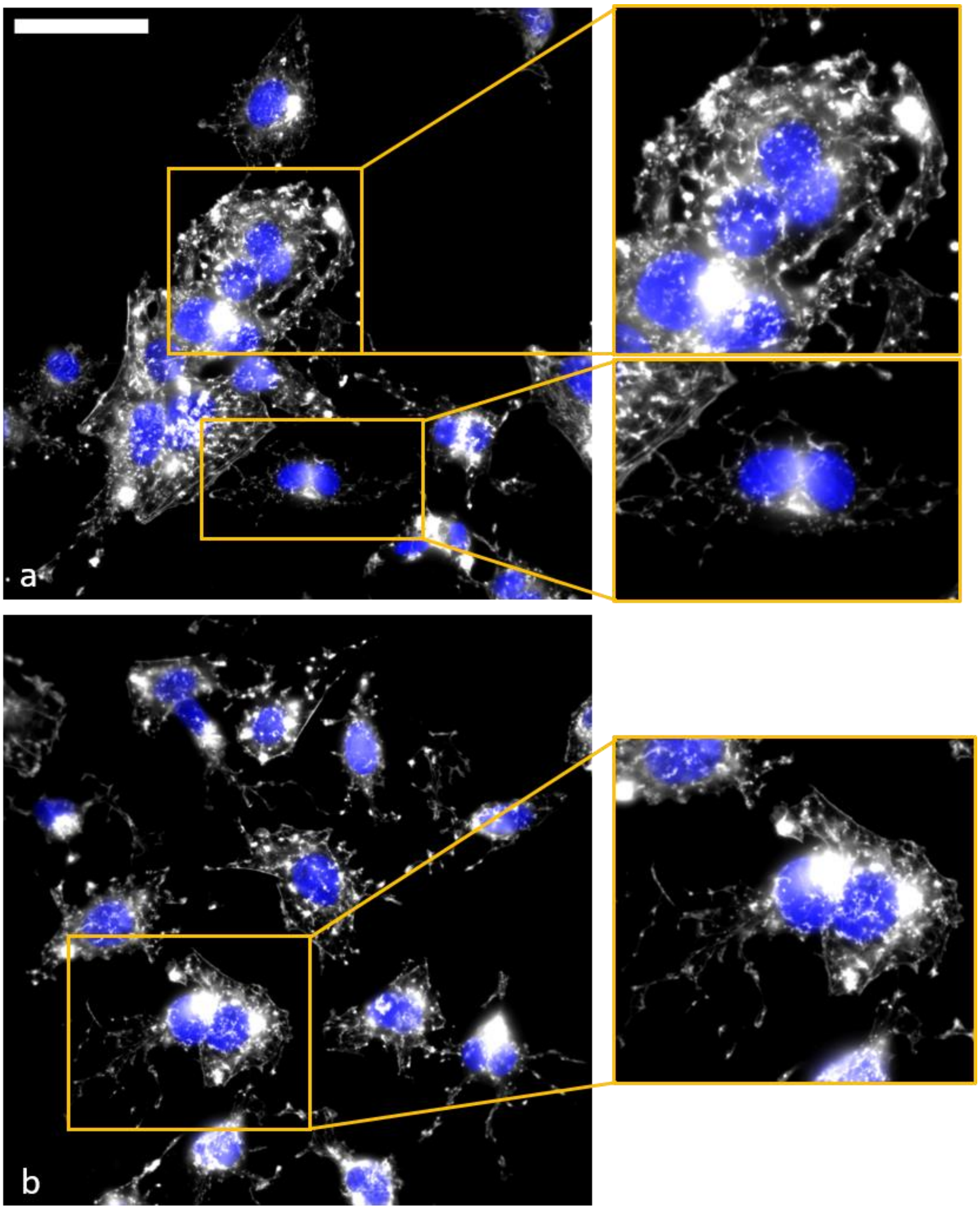

Figure S1: Representative U-2OS cells upon 24 h treatment of **5** (a, b). Examples of multinucleated cells were highlighted and enlarged (see yellow frames). Scale bar in (a) corresponds to 50 µm).

Cell Proliferation Assay

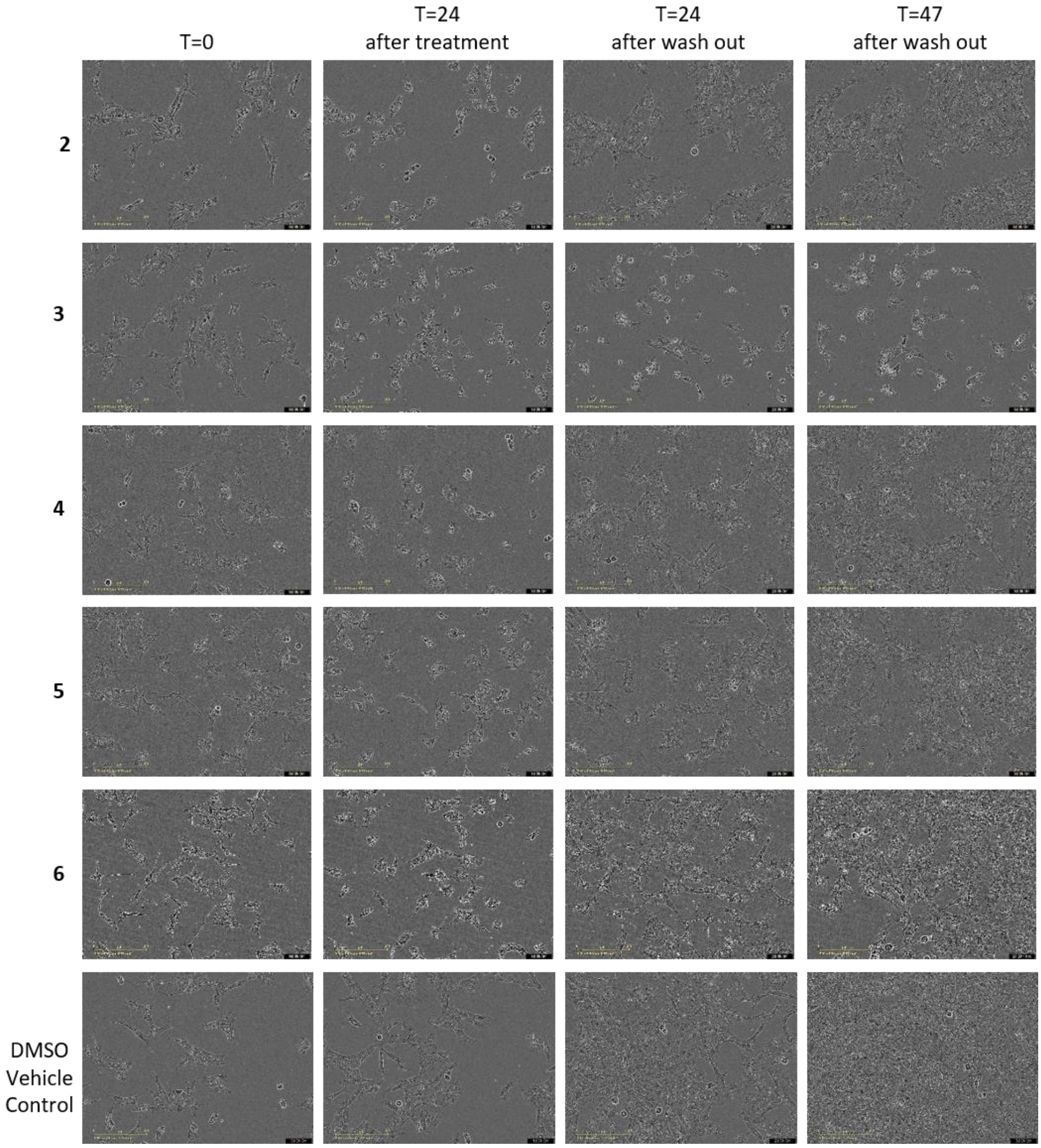

Figure S2: Phase contrast images of U-2OS cells before (T=0) and after 24 h (T=24) treatment with **CB** and **3-6** compared to DMSO, and following wash out and recovery phase (24 h and 47 h wash out).

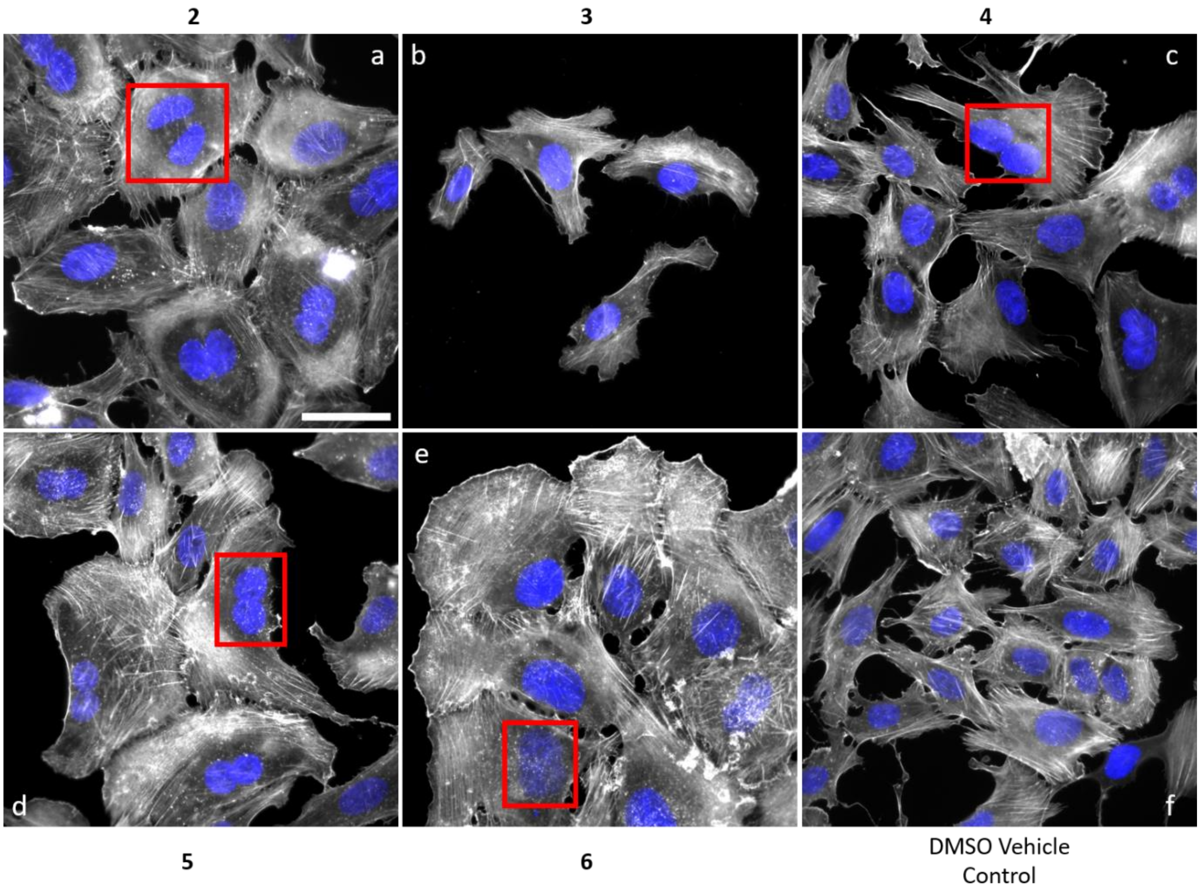

Figure S3: Representative overlay images of long-term high dose **2** and **3-6** treated U-2OS wt cells compared to DMSO as vehicle control (f) followed by 47 h wash-out. Cells were fixed with 4% *para*formaldehyde and stained for their F-actin network using fluorescently labeled phalloidin (greyscale) and their nuclear DNA using DAPI (*pseudo*coloured in blue). The scale bar in image (a) corresponds to 50 µm. A full recovery of the F-actin network was observed for all compounds (a-e), accompanied by some multinucleated cells in case of **2** (a), **3-6** (c-e), highlighted by red frames.

Pyrene assay

Table S1: Polymerization rate [%] of actin describing the relative inhibition of compounds **2**, **3**, **4**, **5**, and **6** based on fluorescence intensities depicted in Figure 4B of the manuscript. The fluorescence intensities at time point 30 min were correlated to the positive control, which was taken as 100 % polymerization.

	Conditions	Polymerization rate [%]
**Control**	2 µM actin (including 5% pyrene-actin) + 550 nM actin seeds	100
**2**	2 µM actin (including 5% pyrene-actin) + 550 nM actin seeds + 2 µM of **2**	45
**3**	2 µM actin (including 5% pyrene-actin) + 550 nM actin seeds + 2 µM **3**	107
**4**	2 µM actin (including 5% pyrene-actin) + 550 nM actin seeds + 2 µM **4**	83
**5**	2 µM actin (including 5% pyrene-actin) + 550 nM actin seeds + 2 µM **5**	86
**6**	2 µM actin (including 5% pyrene-actin) + 550 nM actin seeds + 2 µM **6**	58

Microextraction of compounds

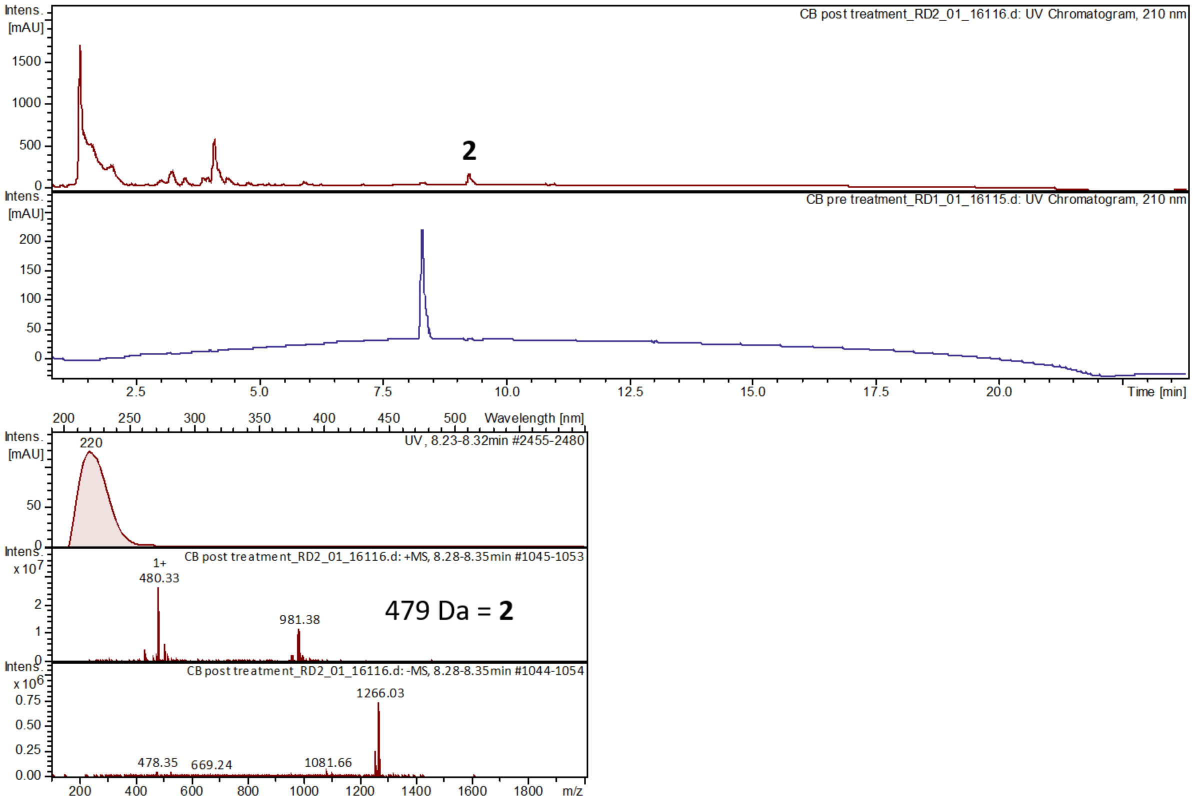

Figure S4: HPLC-MS data (UV chromatogram at 210 nm (top) and ESI-MS spectra of indicated retention times (bottom)) of extracted **2** after 24 h cell exposure (1 mL medium spiked with 3 µg **2**) compared to **2** standard (0.1 mg/mL). **2** was extracted from cell culture medium using EtOAc (2 mL) and measured by ESI-MS. Spectrum showed no conversion of **2** after treatment compared to the standard.

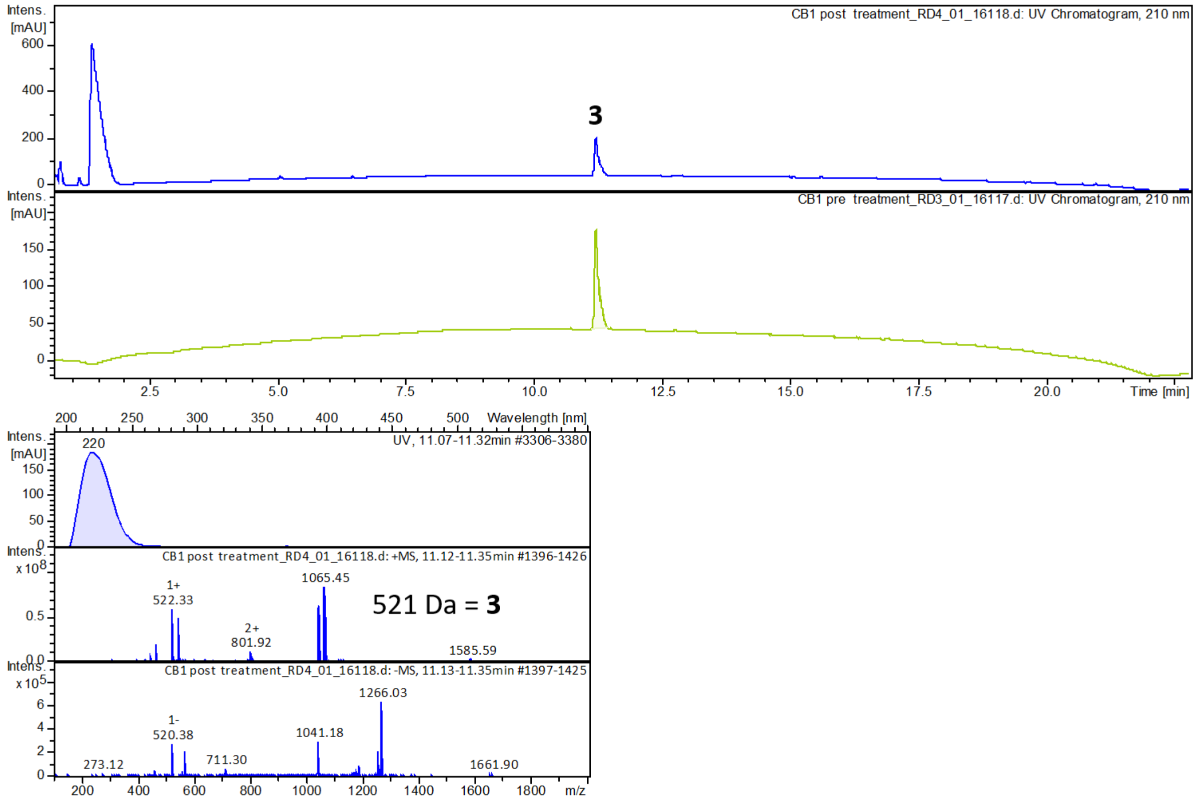

Figure S5: HPLC-MS data (UV chromatogram at 210 nm (top) and ESI-MS spectra of indicated retention times (bottom)) of extracted **3** after 24 h cell exposure (1 mL medium spiked with 41.5 µg **3**) compared to **3** standard (0.1 mg/mL). **3** was extracted from cell culture medium using EtOAc (2 mL) and measured by ESI-MS. Spectrum showed no conversion of **3** after treatment compared to the standard.

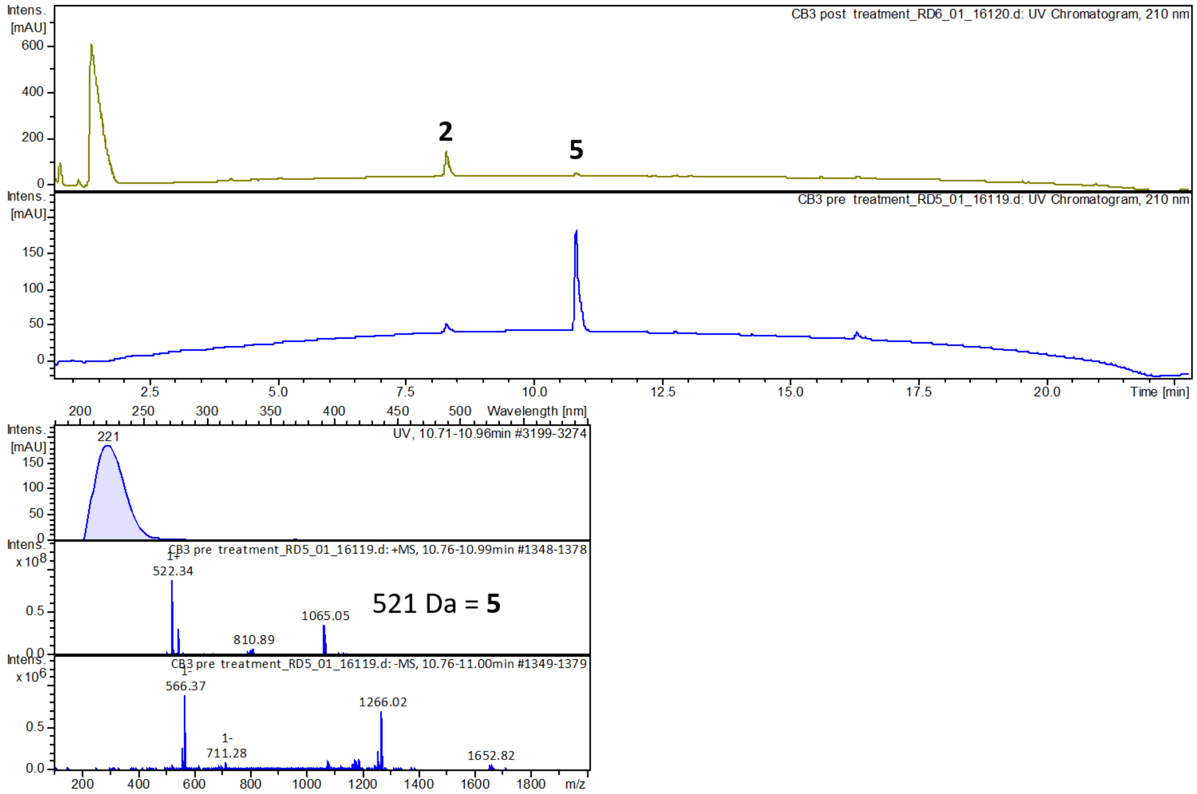

Figure S6: HPLC-MS data (UV chromatogram at 210 nm (top) and ESI-MS spectra of indicated retention times (bottom)) of extracted **5** after 24 h cell exposure (1 mL medium spiked with 24.5 µg **5**) compared to **5** standard (0.1 mg/mL). **5** was extracted from cell culture medium using EtOAc (2 mL) and measured by ESI-MS. Data indicates a significant conversion of **5** to **2** after treatment.

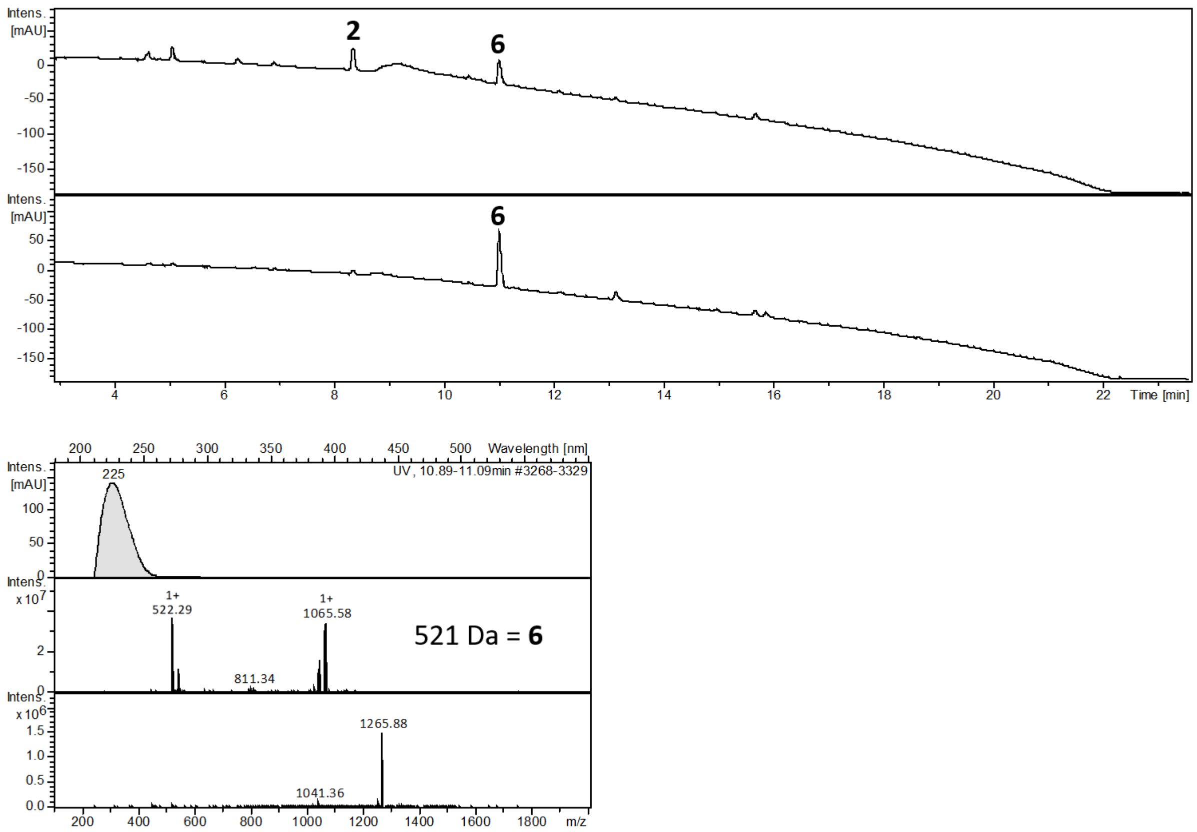

Figure S7: HPLC-MS data (UV chromatogram at 210 nm (top) and ESI-MS spectra of indicated retention times (bottom)) of extracted **6** after 24 h cell exposure (1 mL medium spiked with 12.5 µg **6**) compared to **6** standard (0.1 mg/mL). **6** was extracted from cell culture medium using EtOAc (2 mL) and measured by ESI-MS. Data indicate a partially conversion of **6** to **2** after treatment.

References

[1] R. Kretz, L. Wendt, S. Wongkanoun, J. Luangsa-ard, F. Surup, S. Helaly, S. Noumeur, M. Stadler, T. Stradal, *Biomolecules* **2019**, *9*, 73.

[2] D. Breitsprecher, A. K. Kiesewetter, J. Linkner, J. Faix, *Methods Mol. Biol.* **2009**, *571*, 401–15.

[3] N. Branda, *JoVE Sci. Educ. Database. Org. Chem.* **2019**.

[4] W. C. Still, M. Kahn, A. Mitra, *J. Org. Chem.* **1978**, *43*, 2923–2925.

Appendix (NMR, IR and Mass Spectra)

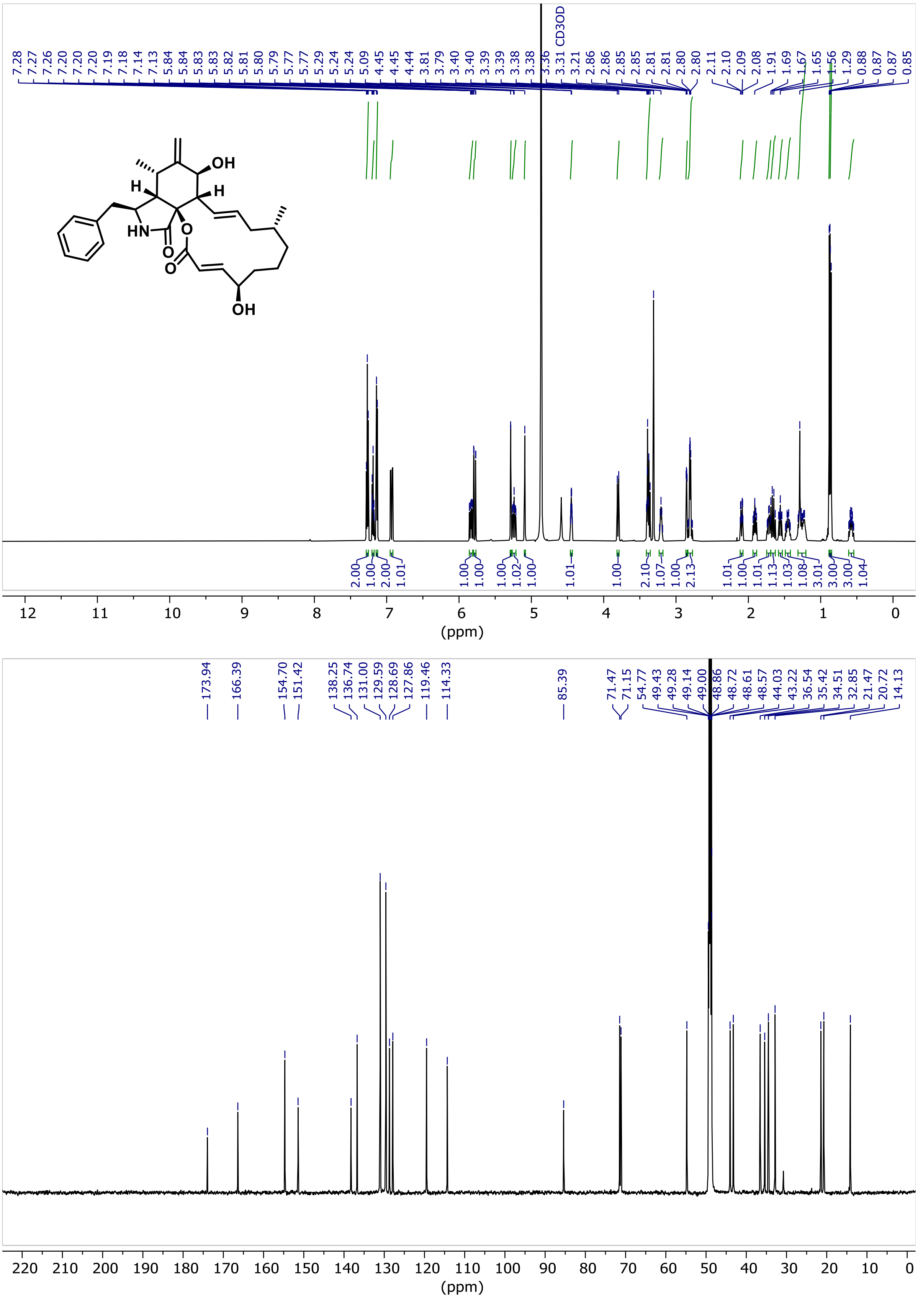

Figure S8: Cytochalasin B (**2**) (**^{1}H-NMR** (600 MHz, CD_3OD) and **^{13}C-NMR** (151 MHz, CD_3OD))

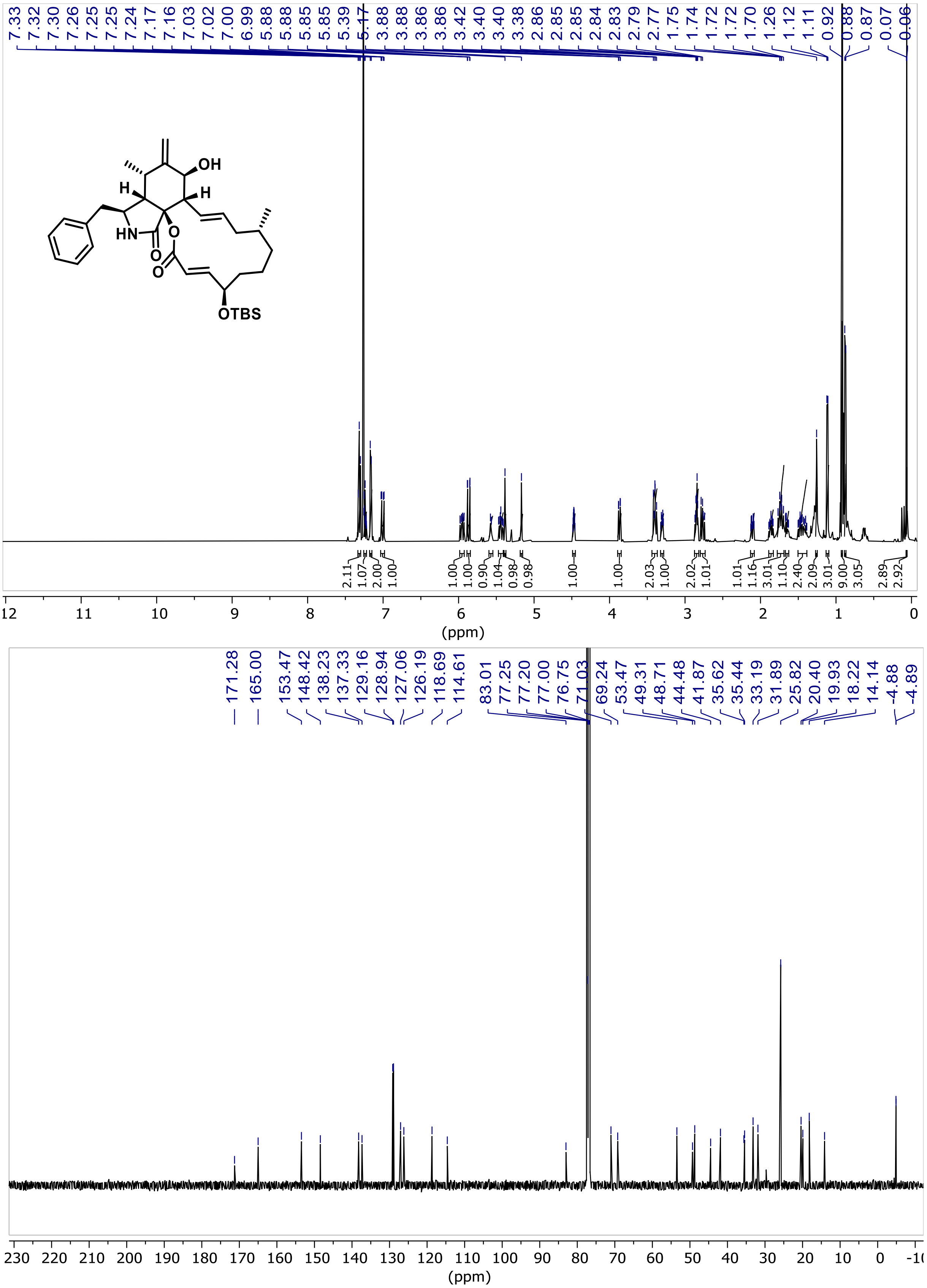

Figure S9: Compound **7** ([1]**H-NMR** (500 MHz, $CDCl_3$) and [13]**C-NMR** (126 MHz, $CDCl_3$))

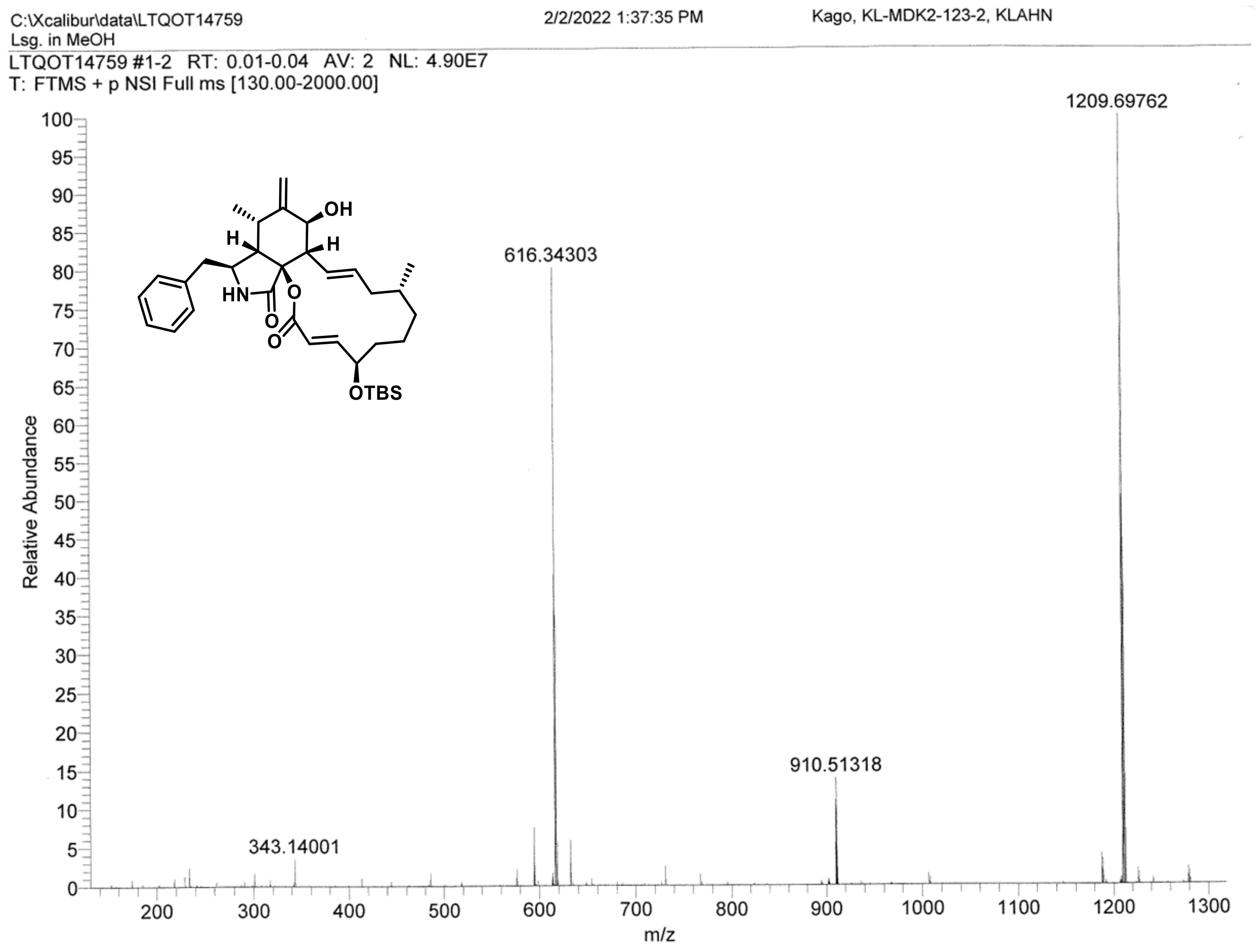

Figure S10: Compound **7** (**HRMS** (ESI))

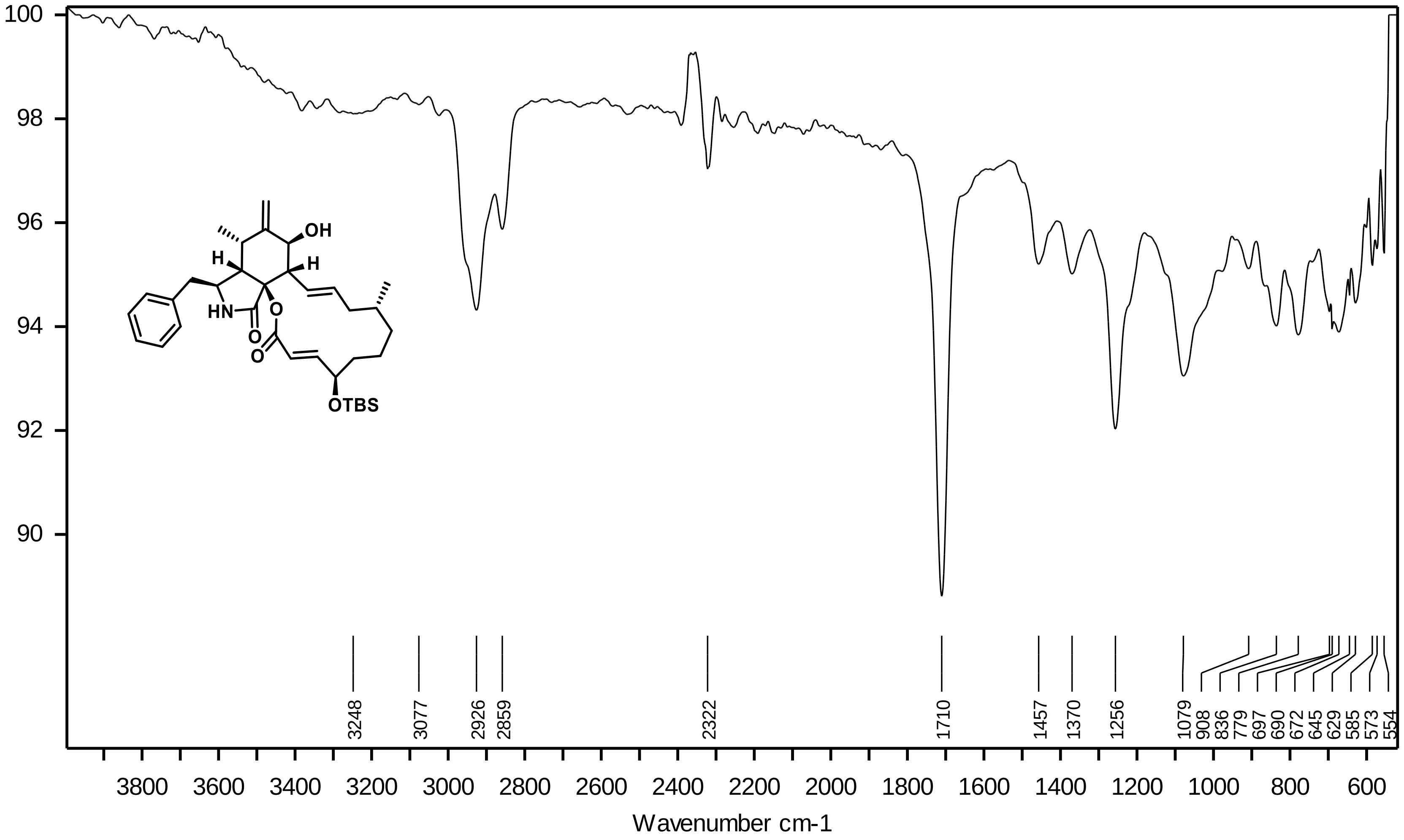

Figure S11: Compound **7** (**IR** (ATR))

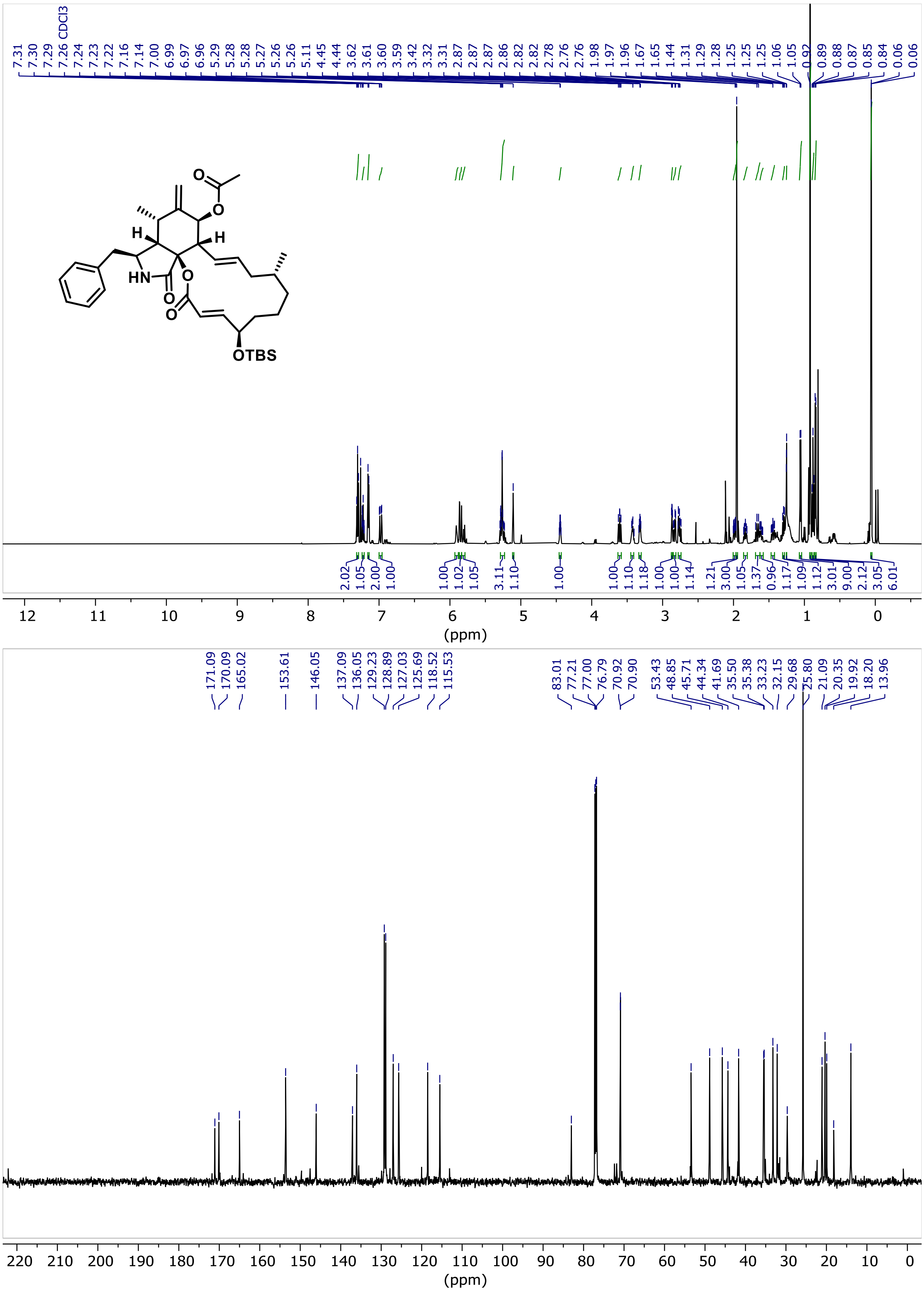

Figure S12: Compound **8** (**^{1}H-NMR** (600 MHz, $CDCl_3$) and **^{13}C-NMR** (151 MHz, $CDCl_3$))

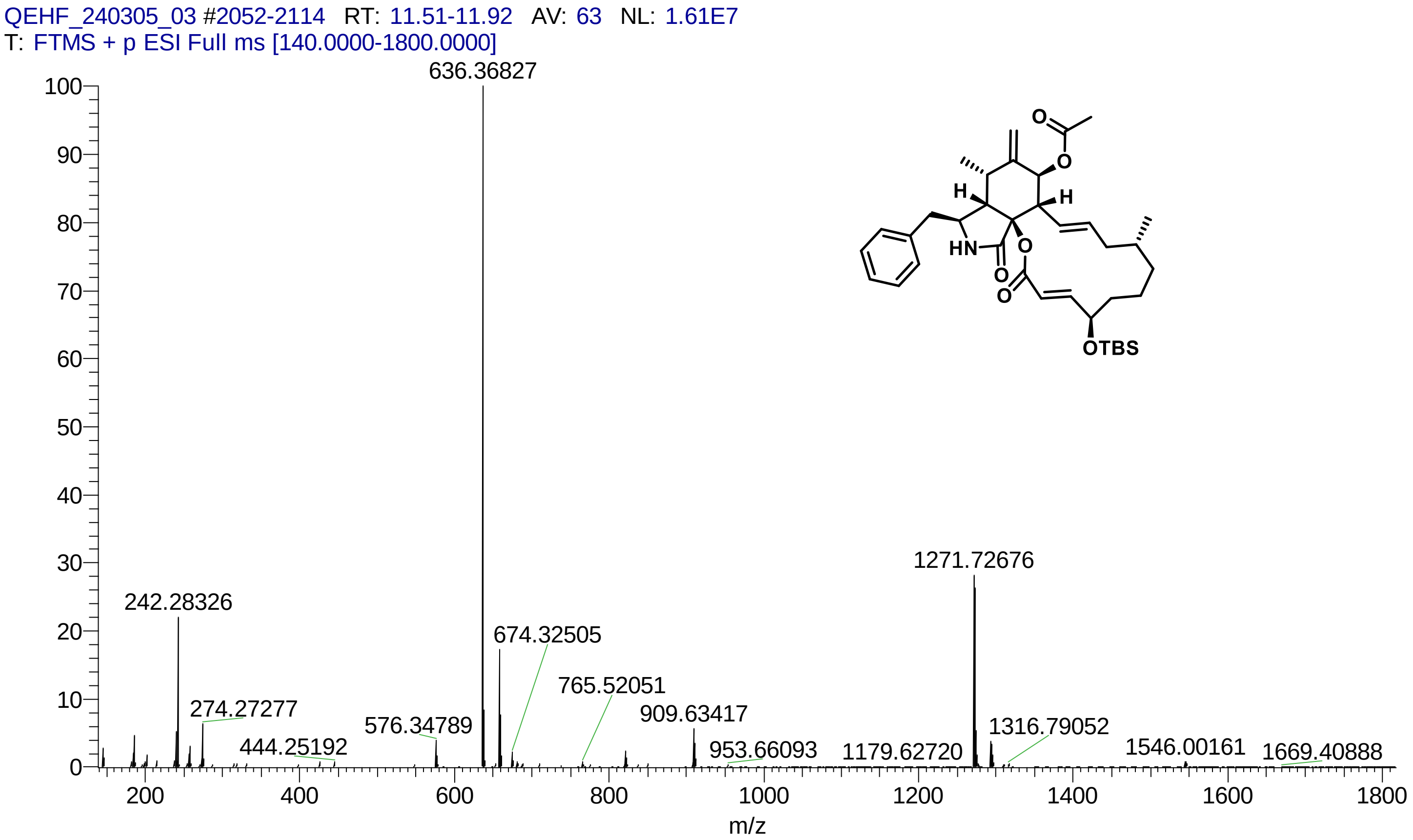

Figure S13: Compound **8** (**HRMS** (ESI))

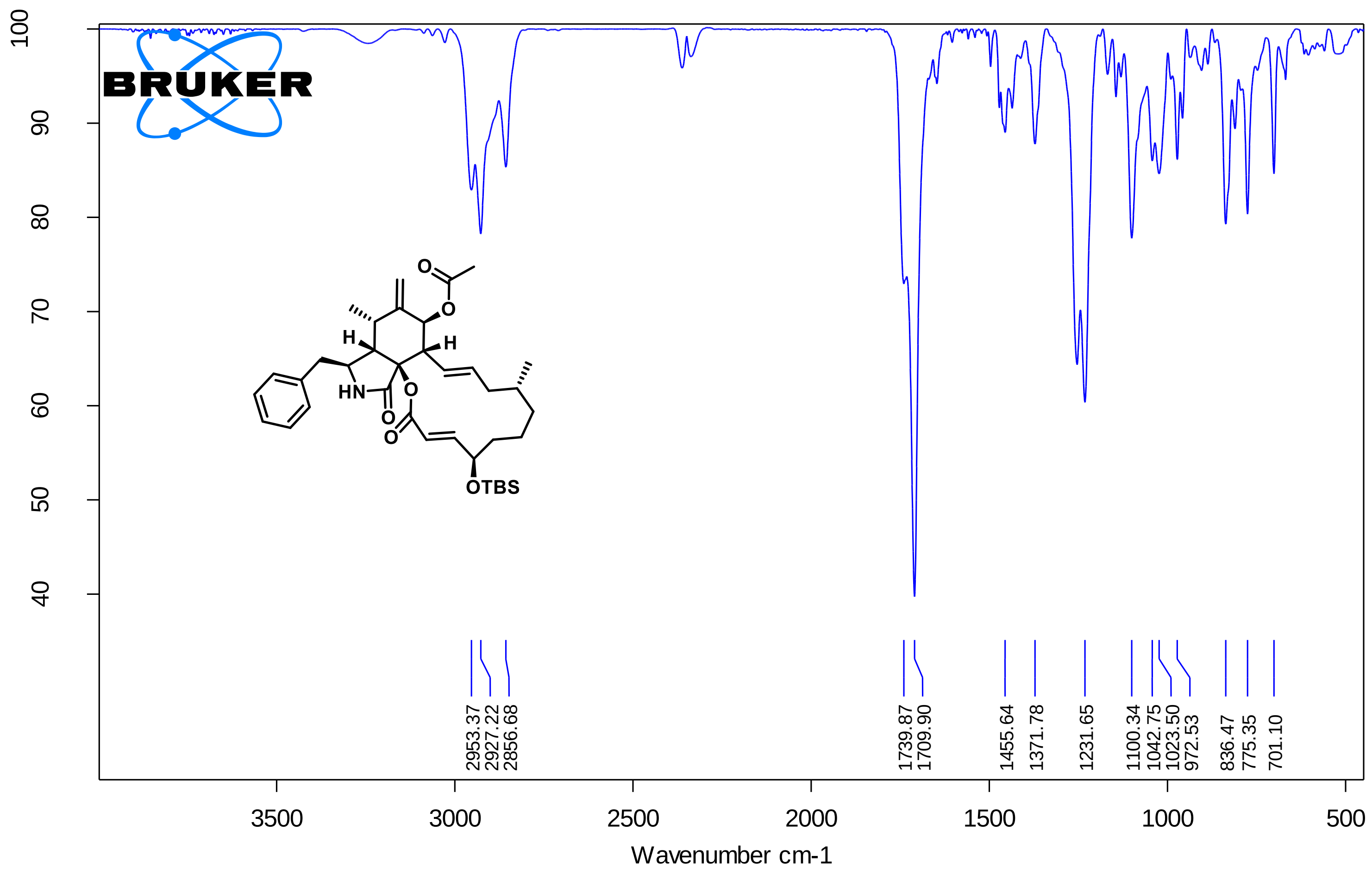

C:\Users\FTIR\Desktop\Data\Mervic\KL-MDK4-036.0 KL-MDK4-036 Instrument type and / or accessory	2024-02-29

Page 1/1

Figure S14: Compound **8** (**IR** (ATR))

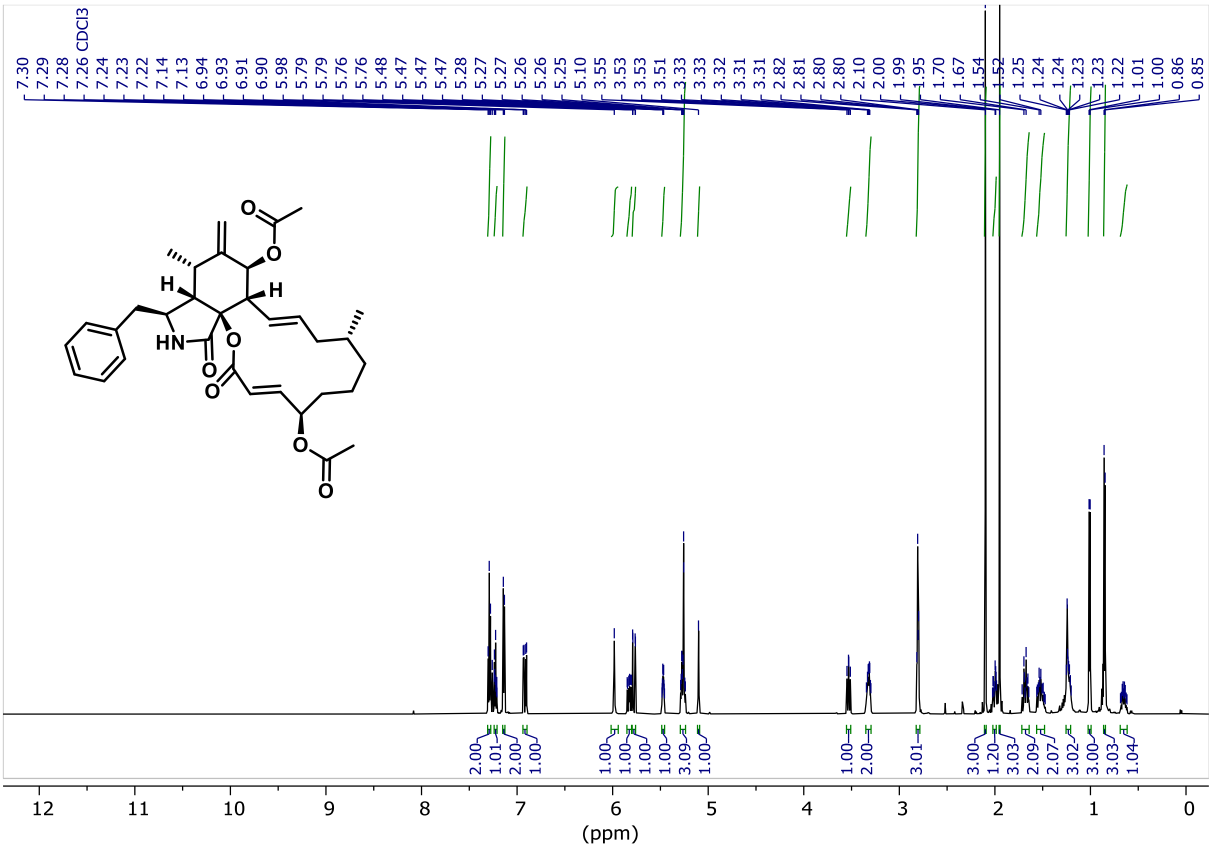

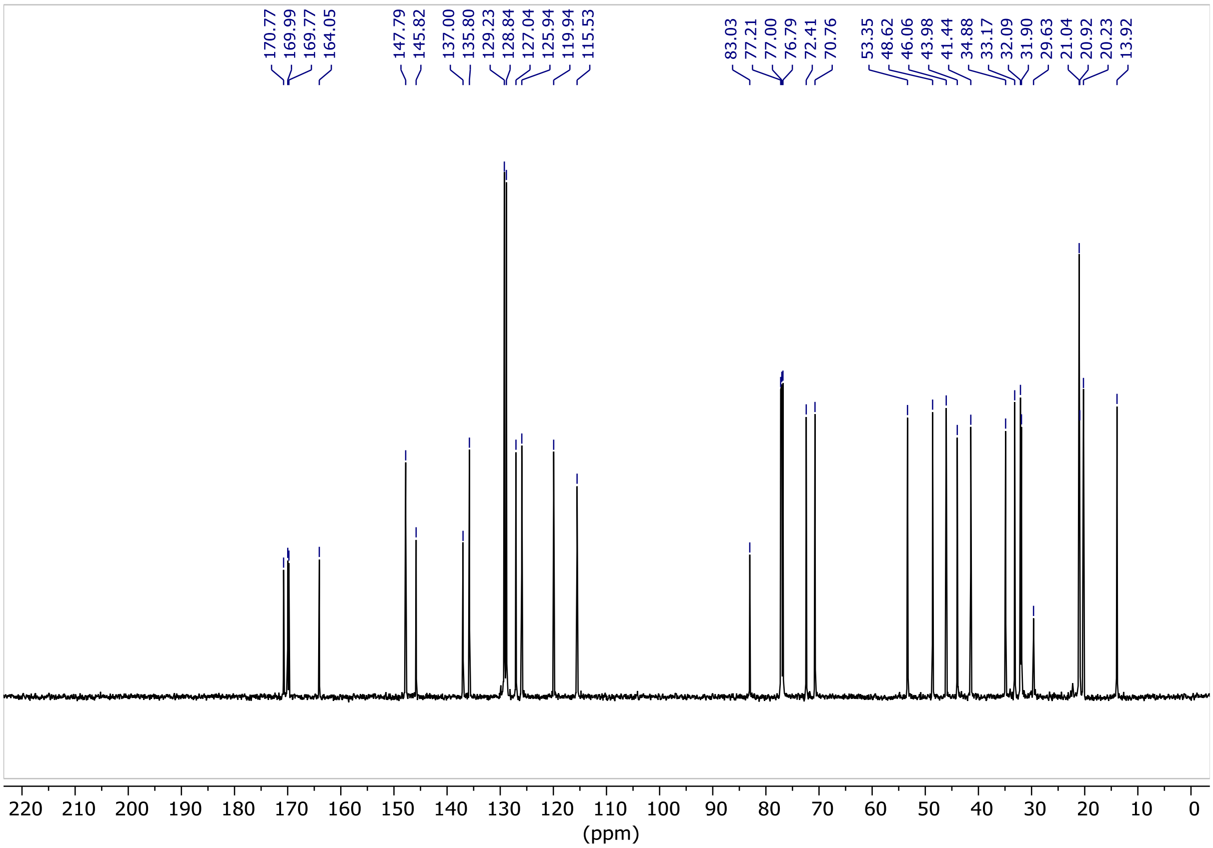

Figure S15: Compound **9** (**^{1}H-NMR** (600 MHz, $CDCl_3$) and **^{13}C-NMR** (151 MHz, $CDCl_3$))

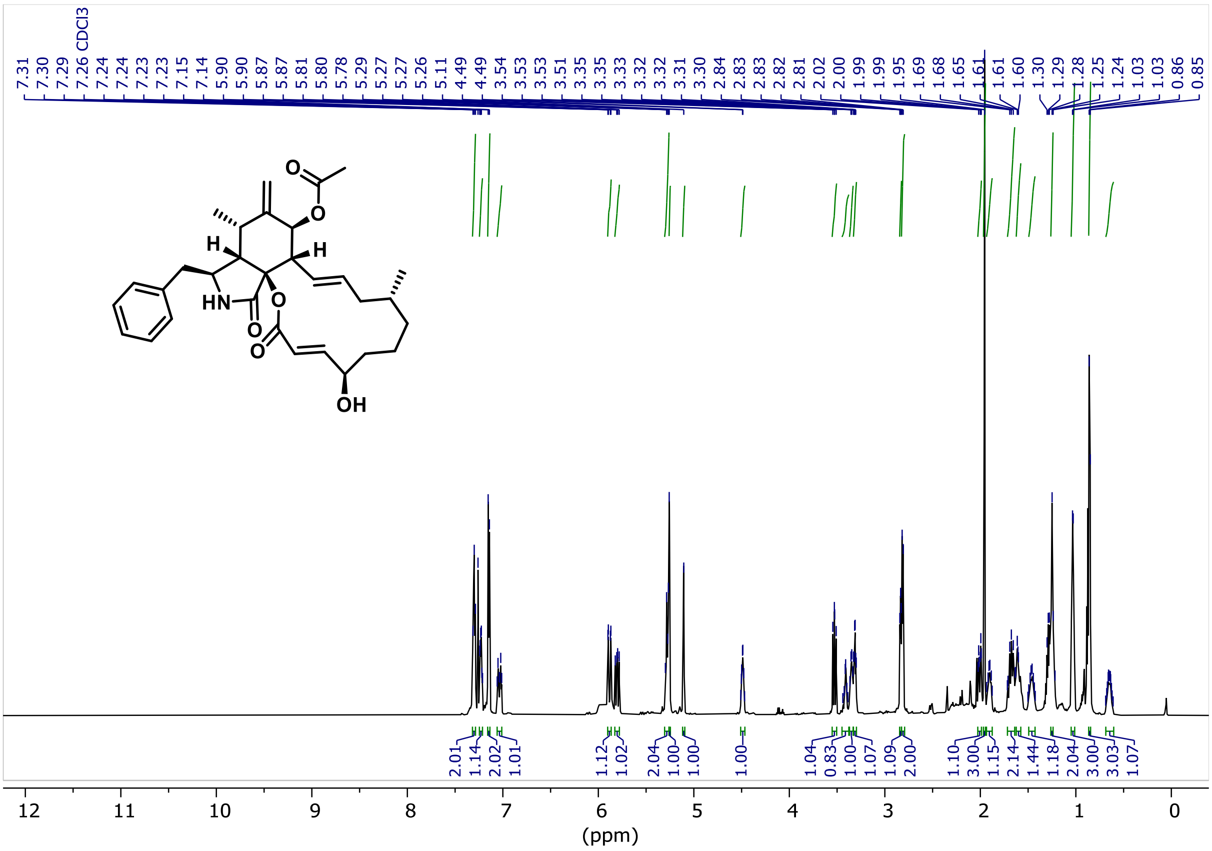

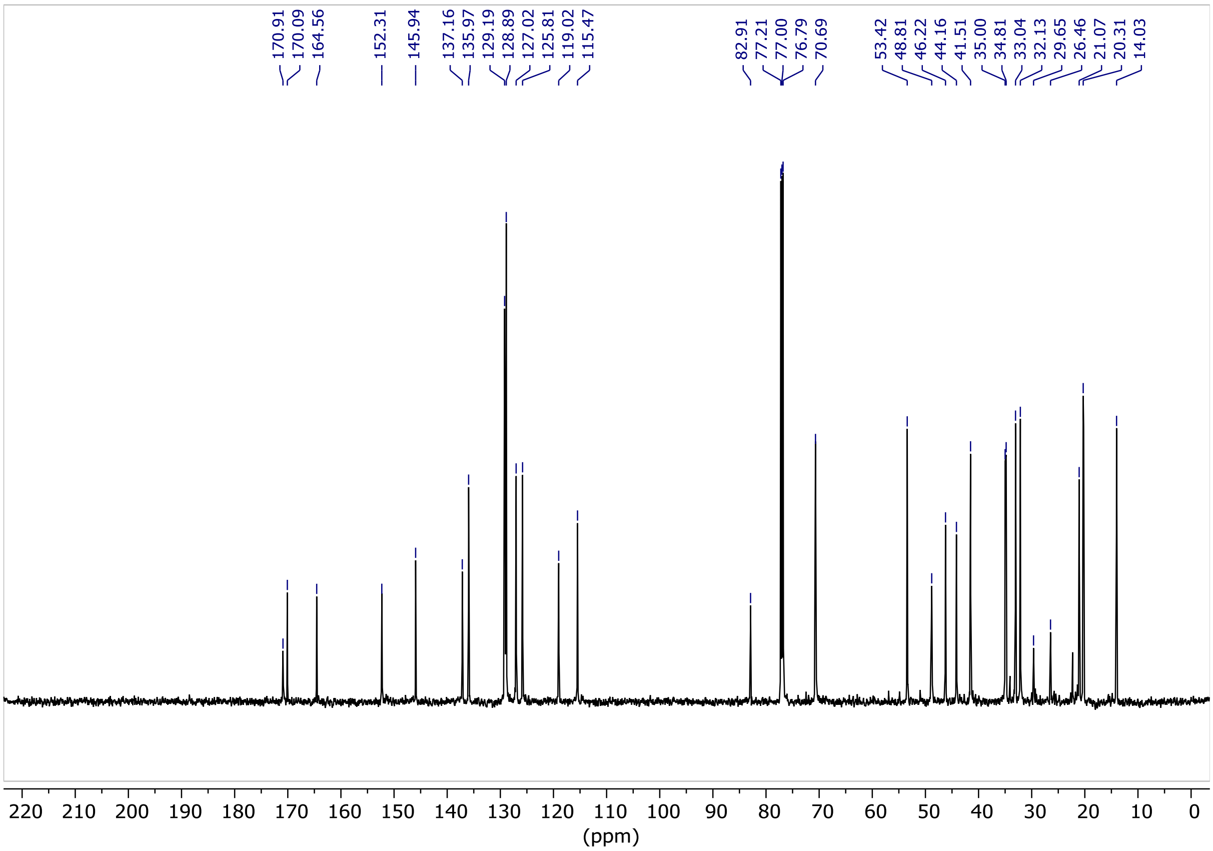

Figure S16: Compound **3** (**^{1}H-NMR** (600 MHz, $CDCl_3$) and **^{13}C-NMR** (151 MHz, $CDCl_3$))

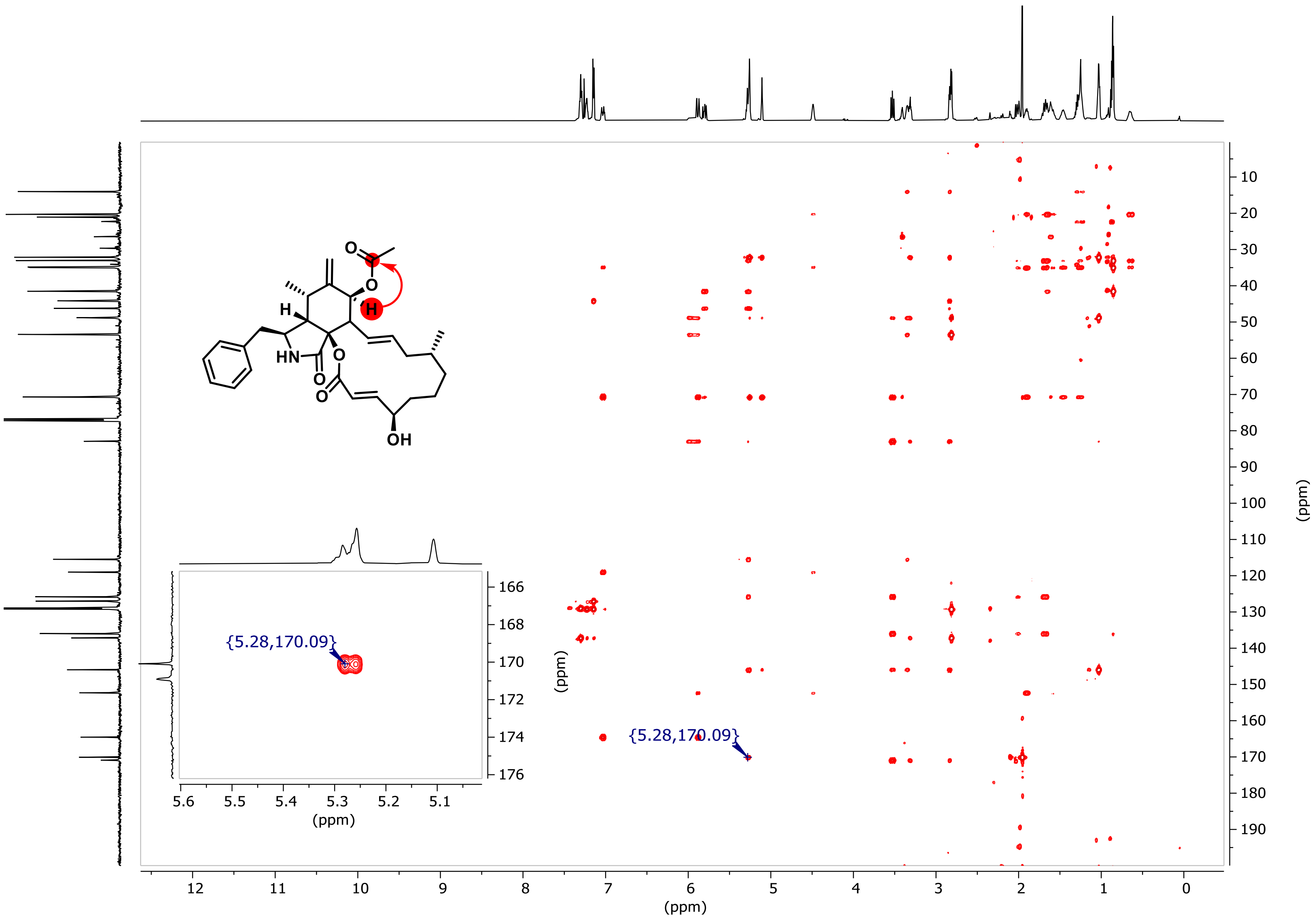

Figure S17: Compound **3** (**HMBC** (600 MHz, $CDCl_3$))

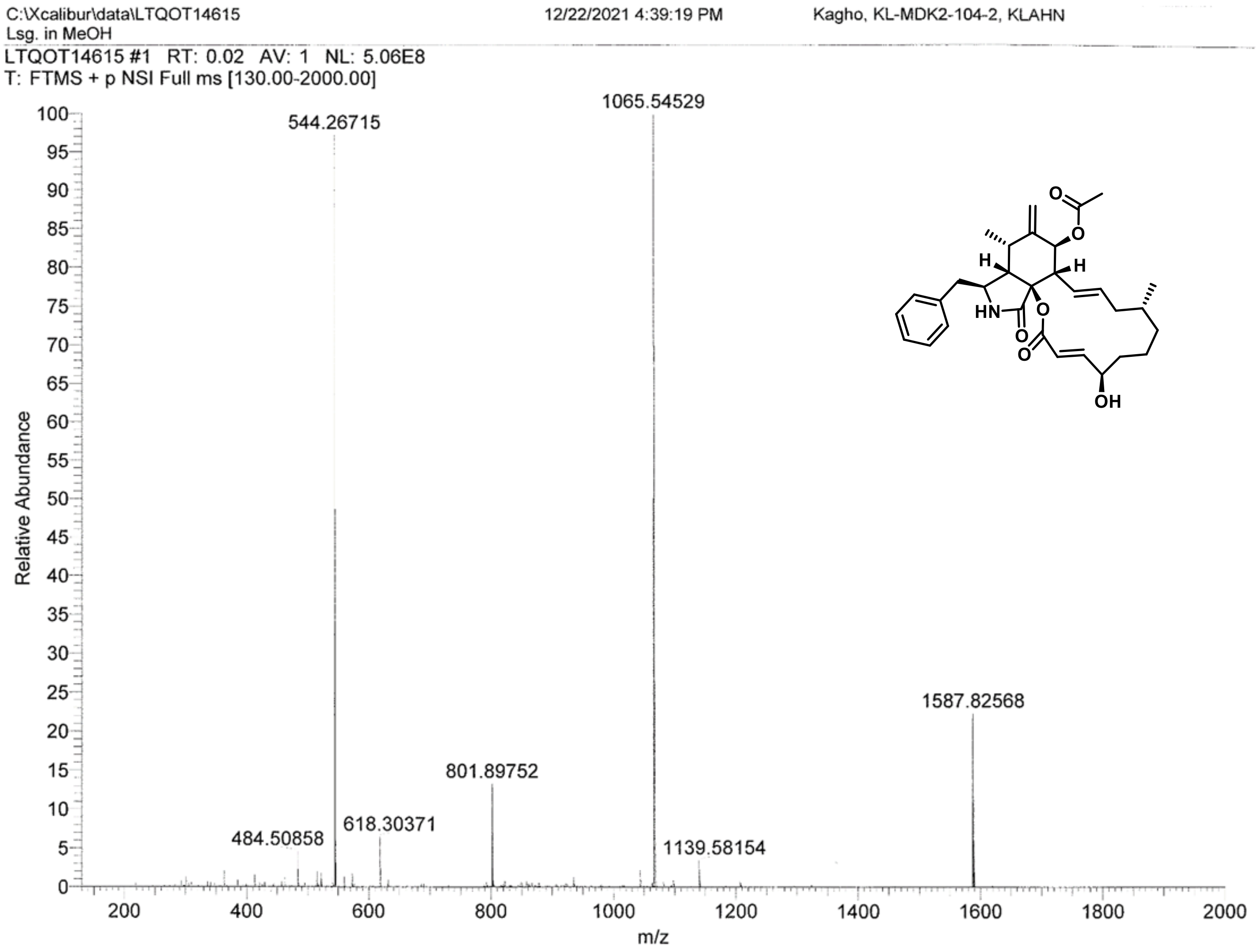

Figure S18: Compound **3** (**HRMS** (ESI))

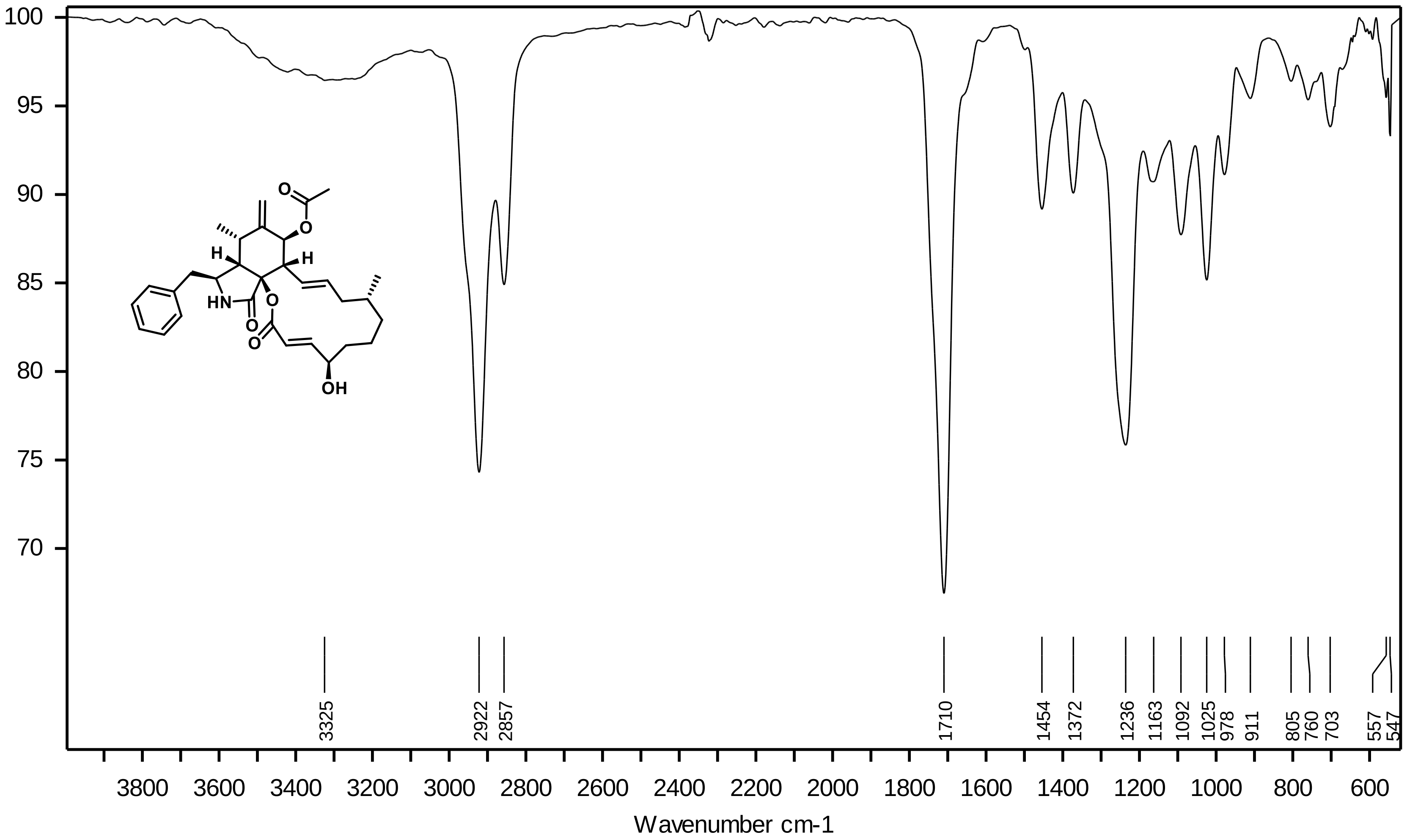

Figure S19: Compound **3** (**IR** (ATR))

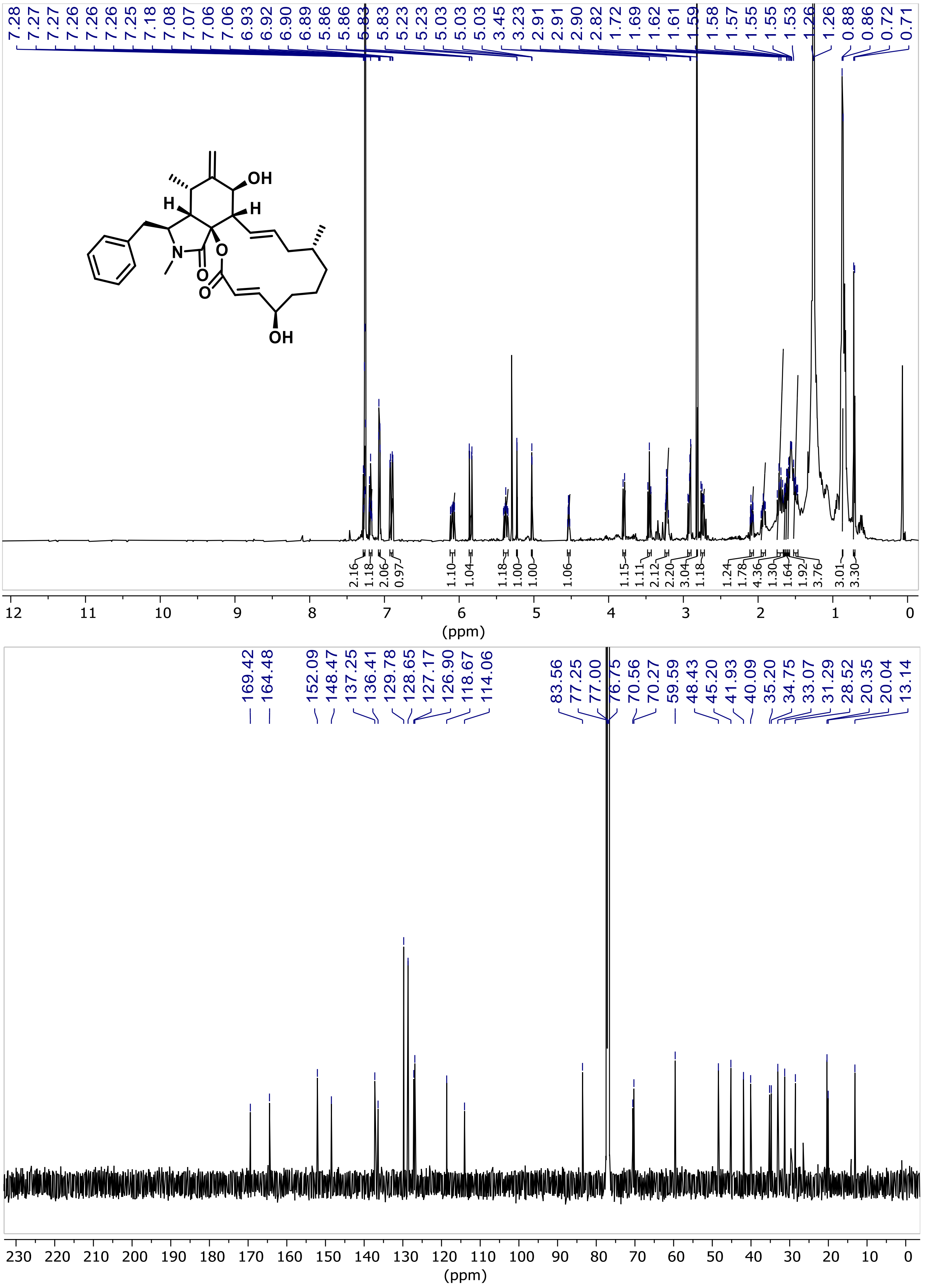

Figure S20: Compound **4** (**^{1}H-NMR** (500 MHz, $CDCl_3$) and **^{13}C-NMR** (126 MHz, $CDCl_3$))

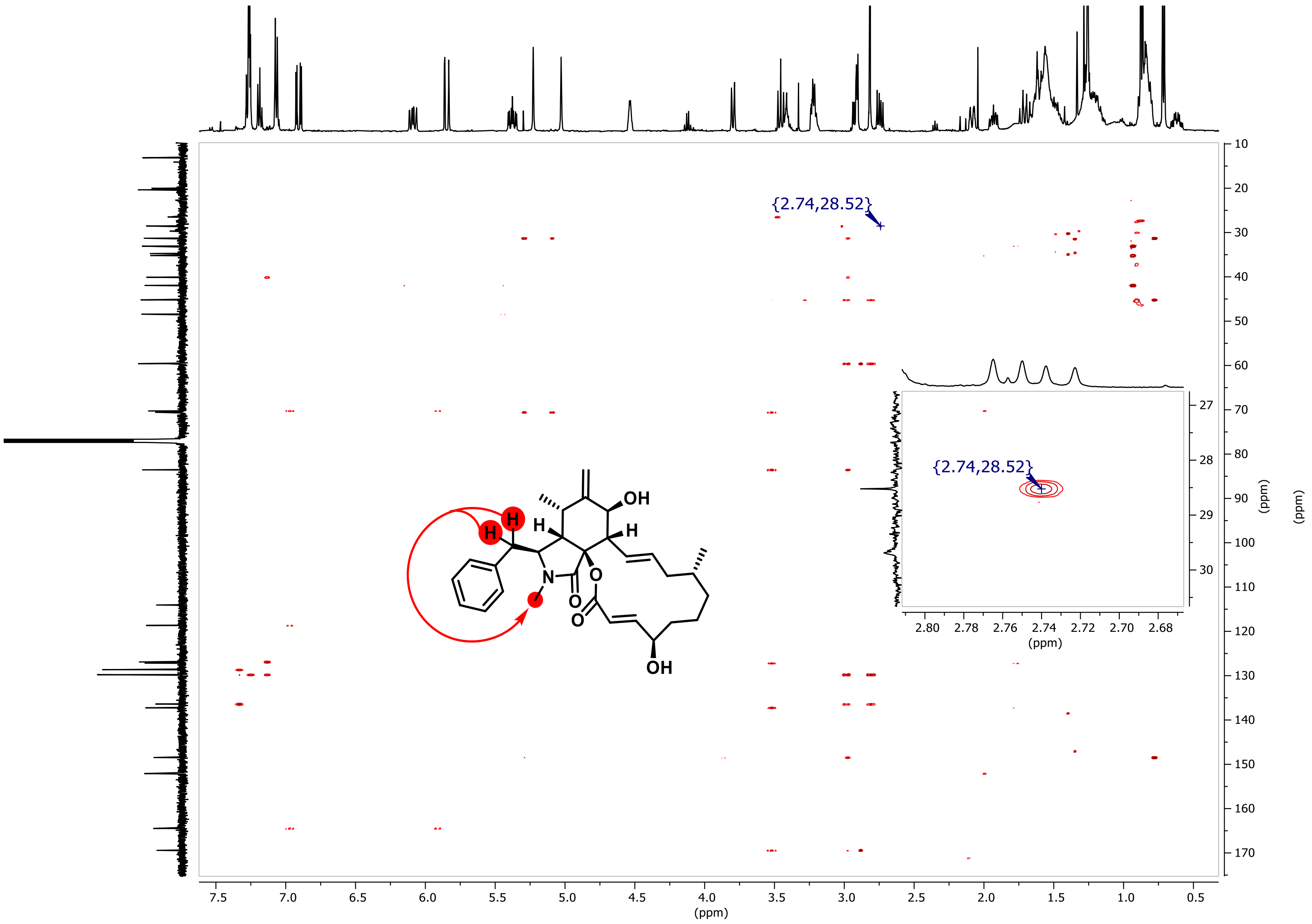

Figure S21: Compound **4** (**HMBC** (500 MHz, $CDCl_3$))

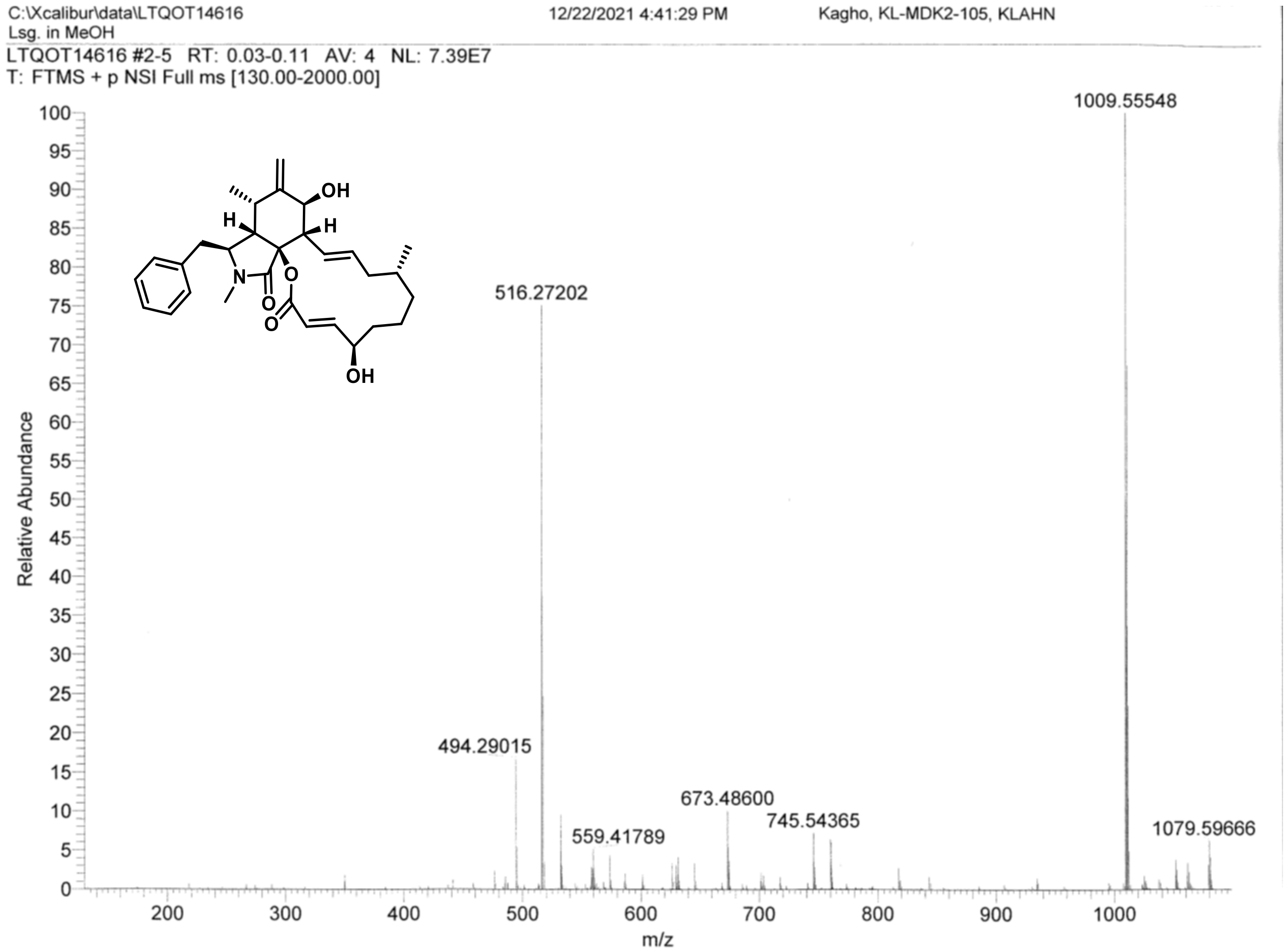

Figure S22: Compound **4** (**HRMS** (ESI))

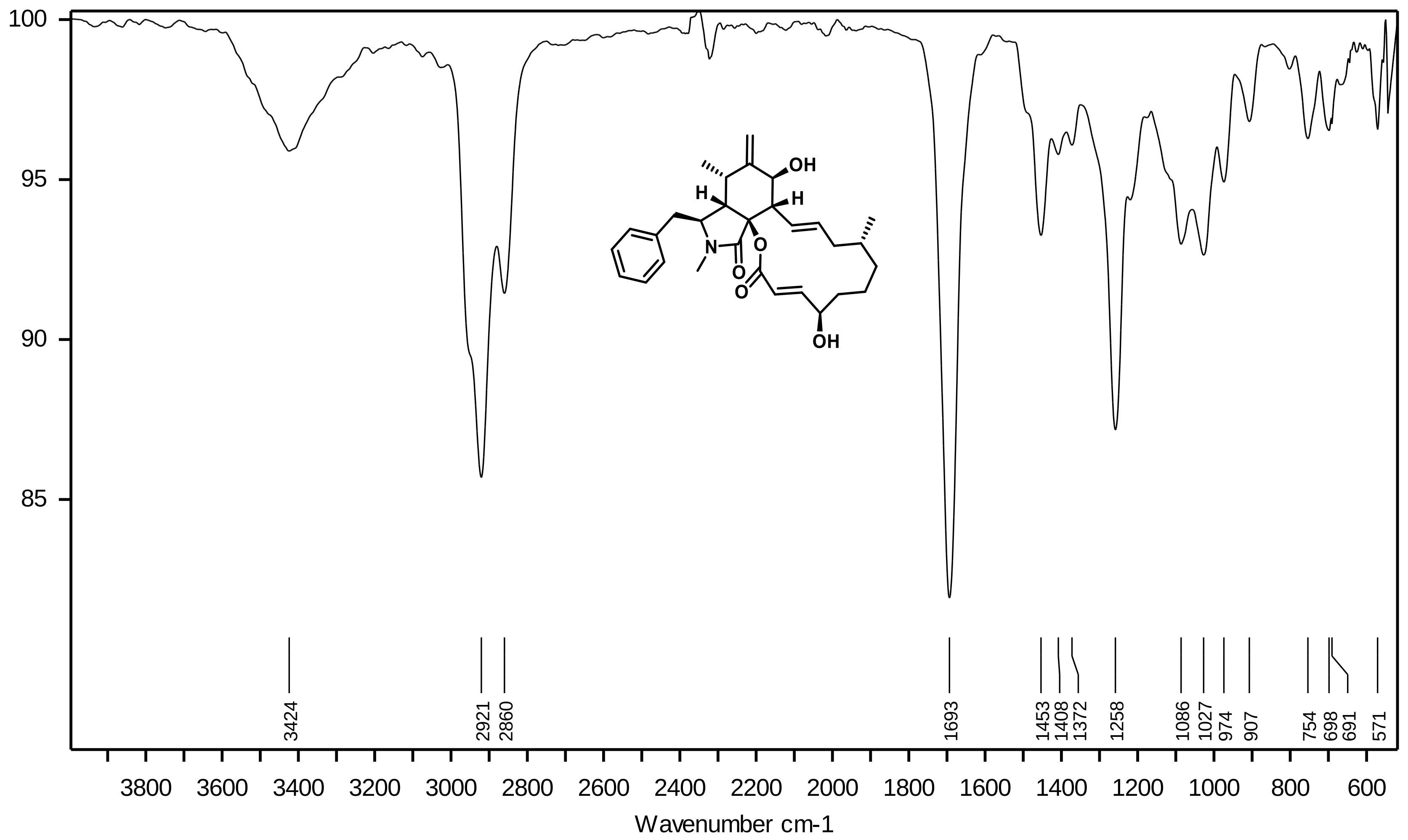

Figure S23: Compound **4** (**IR** (ATR))

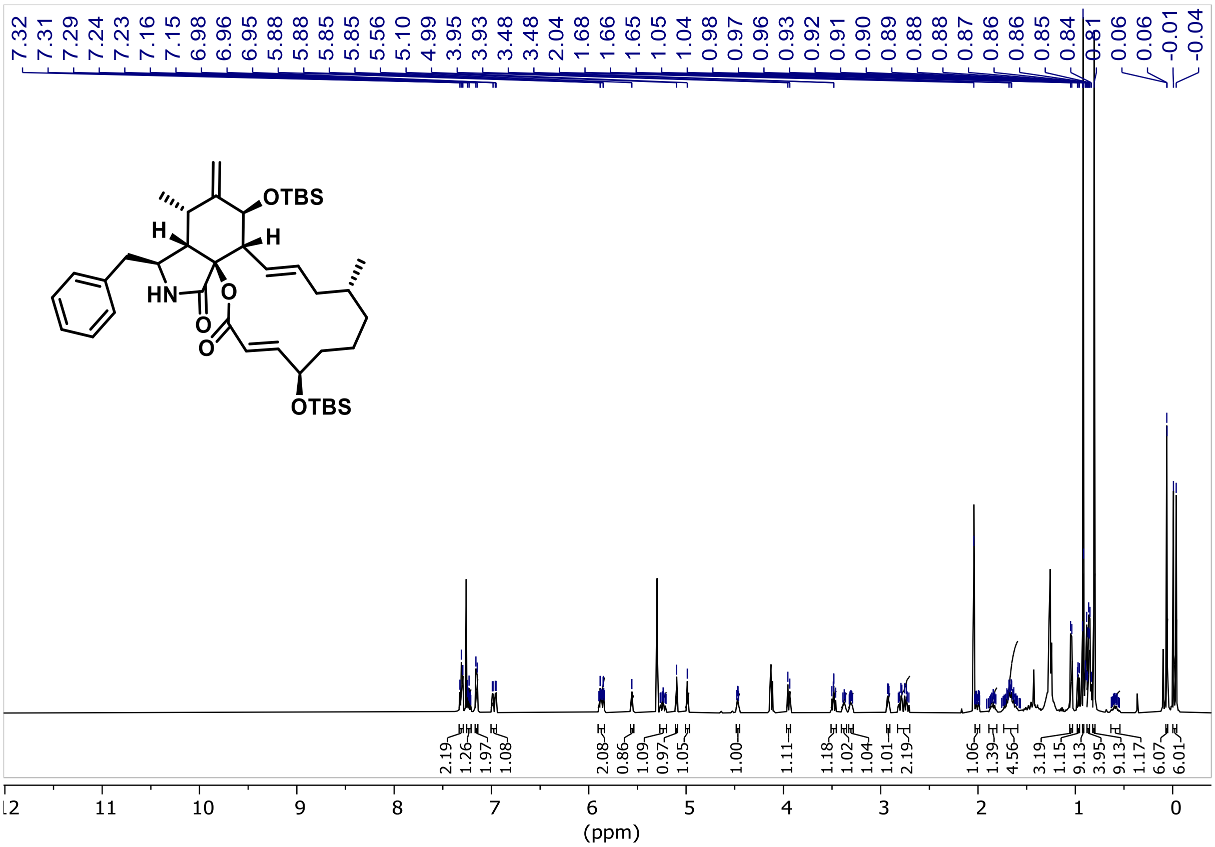

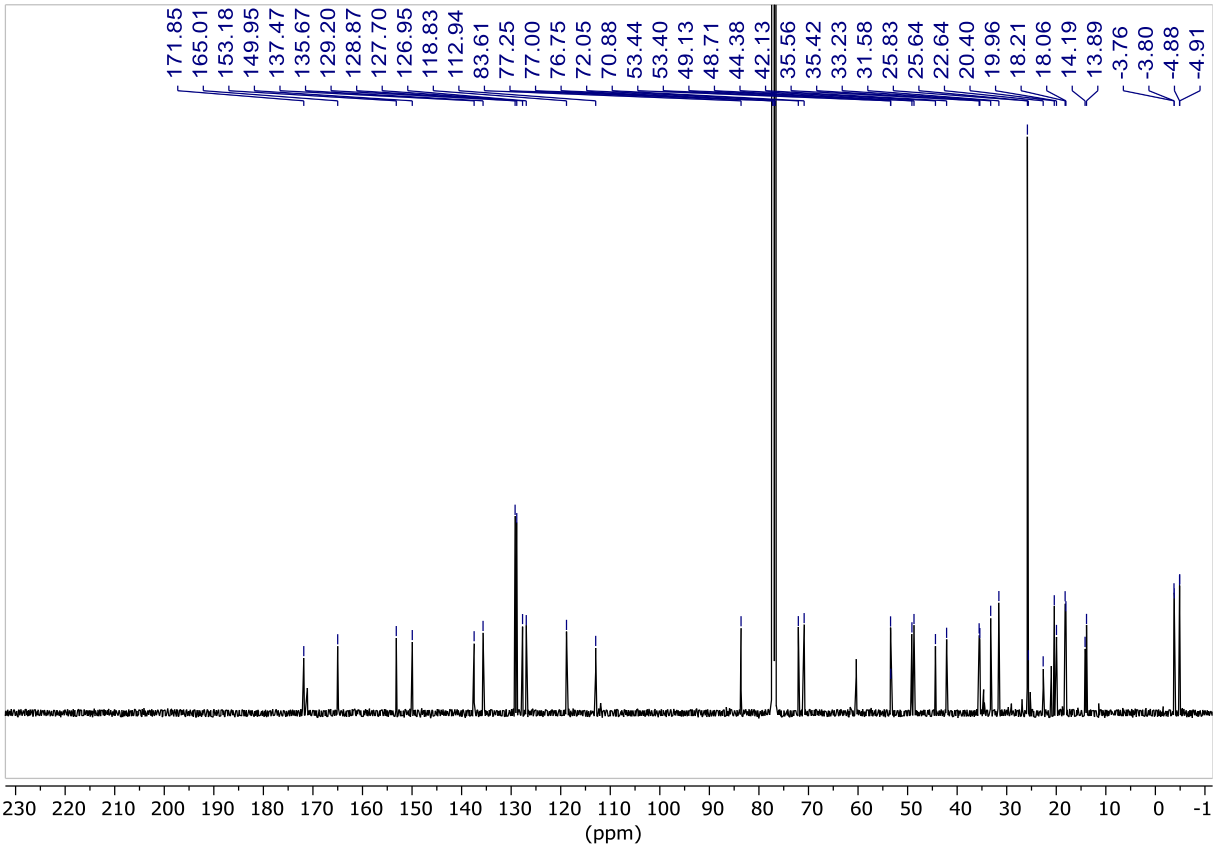

Figure S24: Compound **10** (**^{1}H-NMR** (500 MHz, $CDCl_3$) and **^{13}C-NMR** (126 MHz, $CDCl_3$))

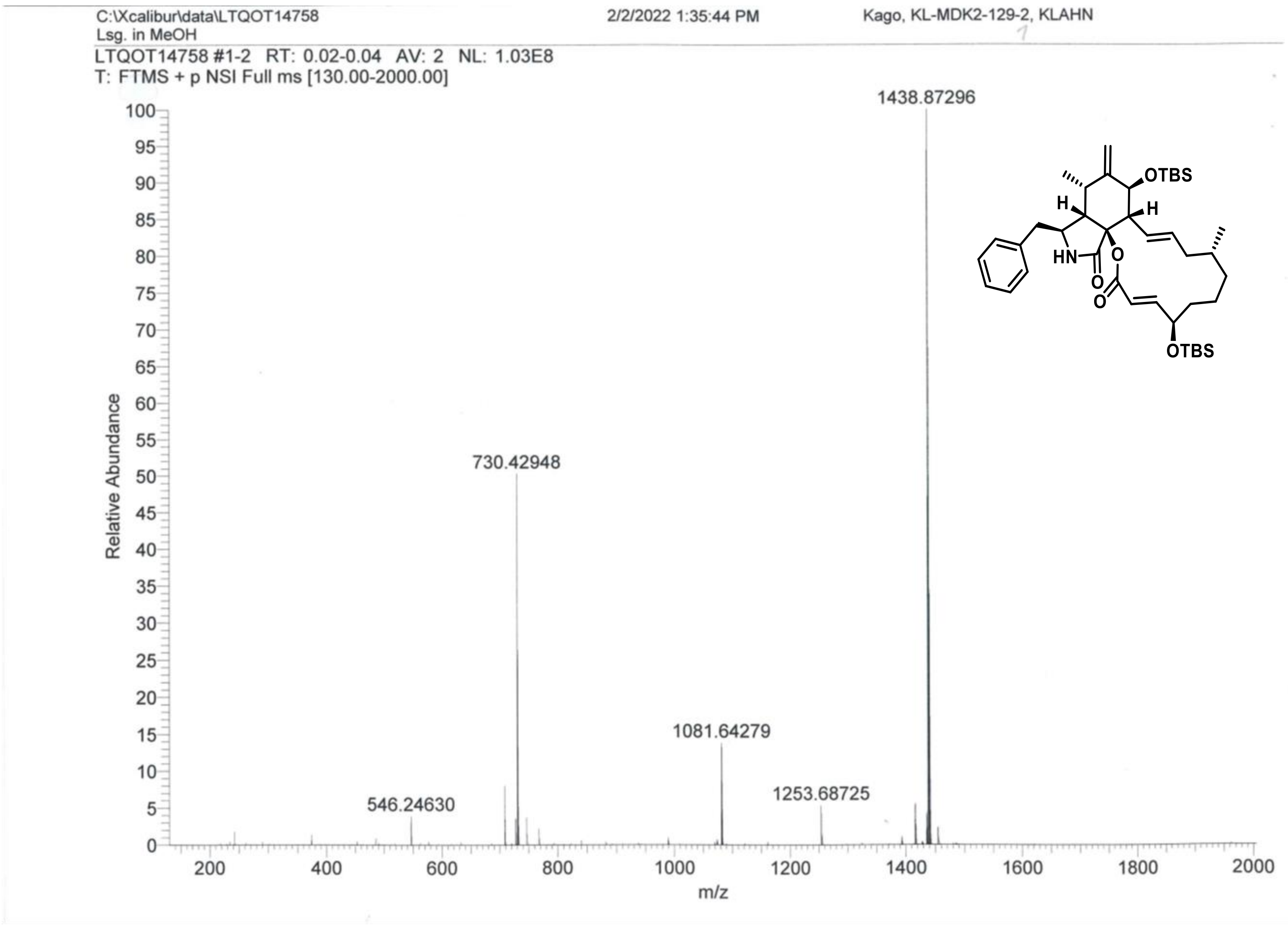

Figure S25: Compound **10** (**HRMS** (ESI))

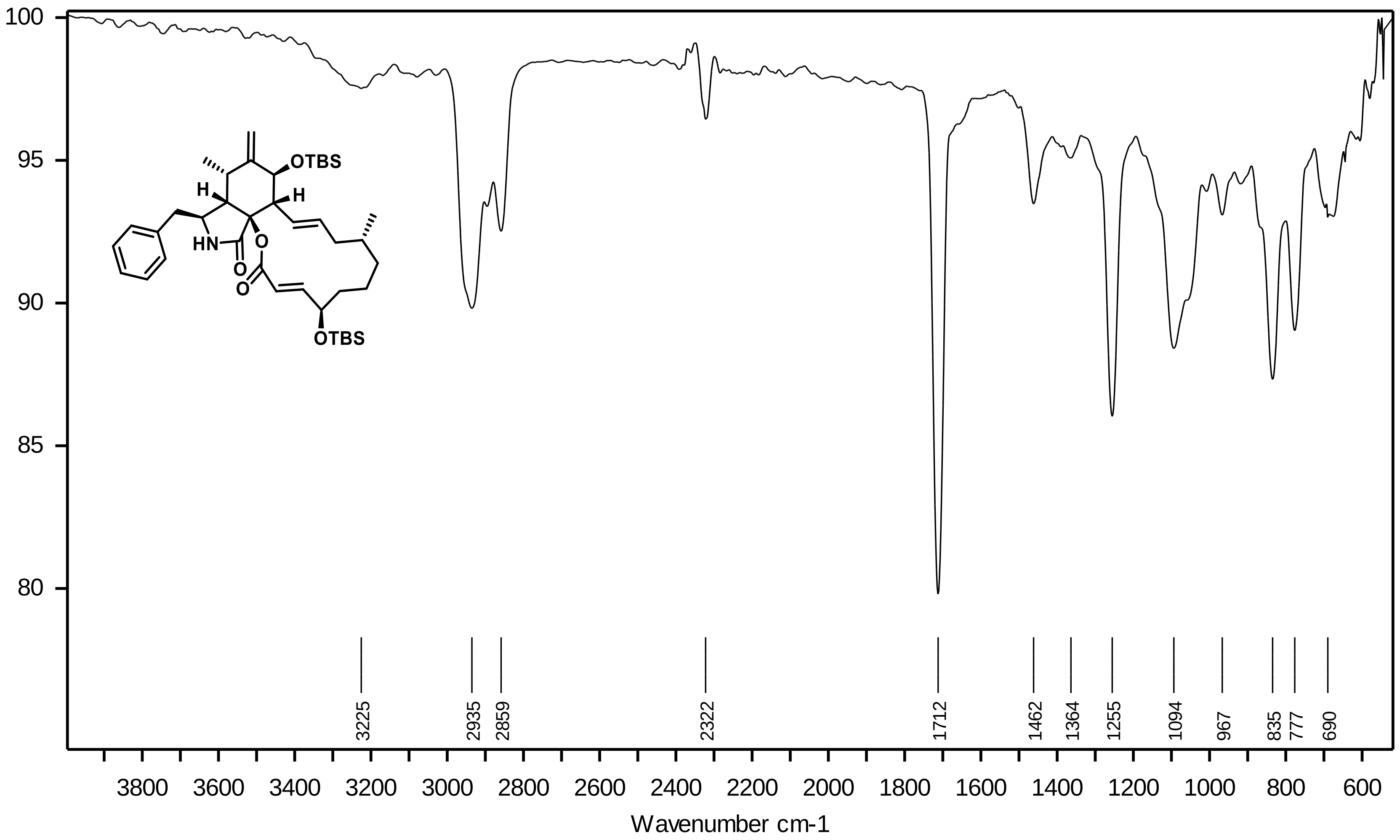

Figure S26: Compound **10** (**IR** (ATR))

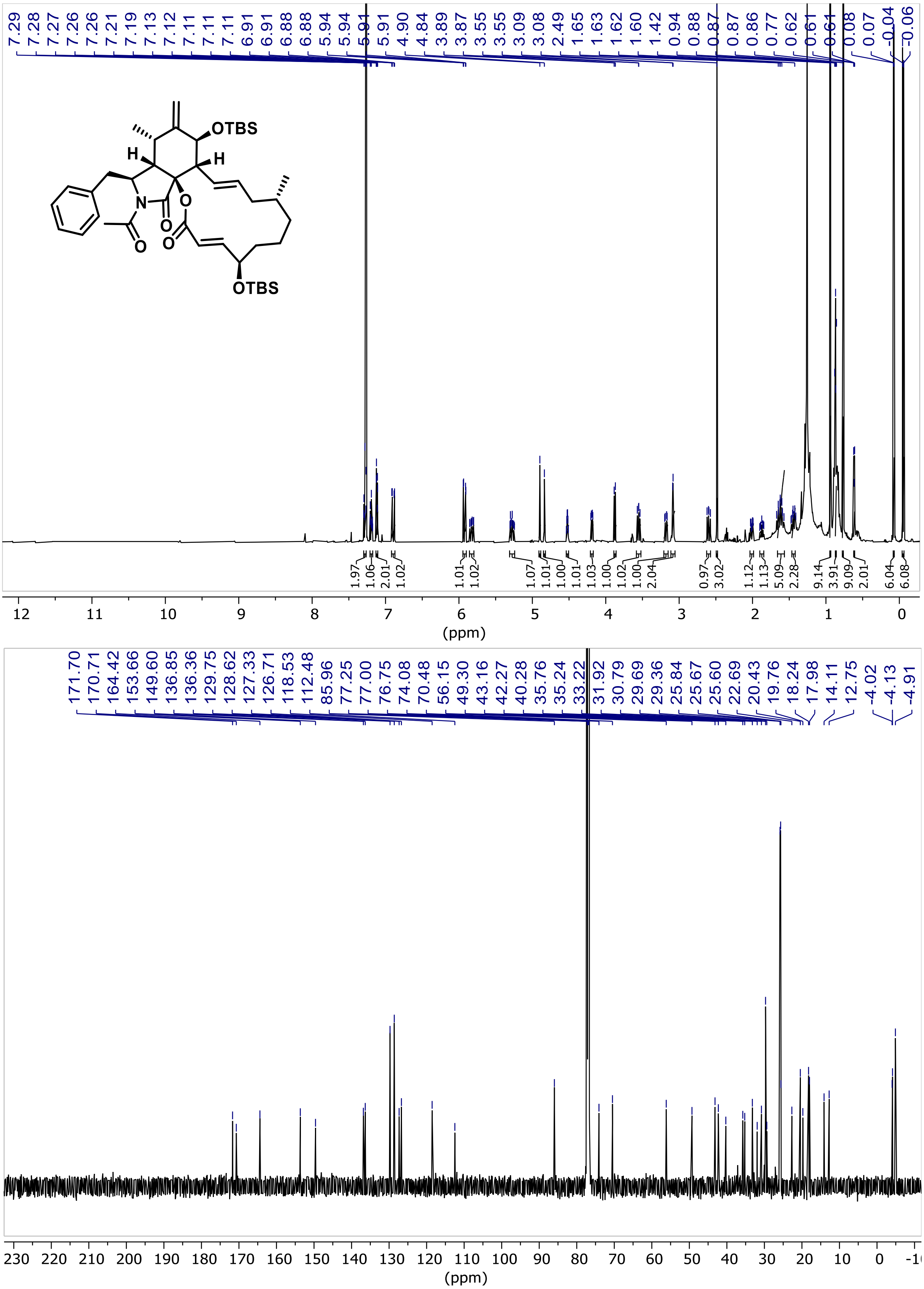

Figure S27: Compound **11** (**^{1}H-NMR** (500 MHz, $CDCl_3$) and **^{13}C-NMR** (126 MHz, $CDCl_3$))

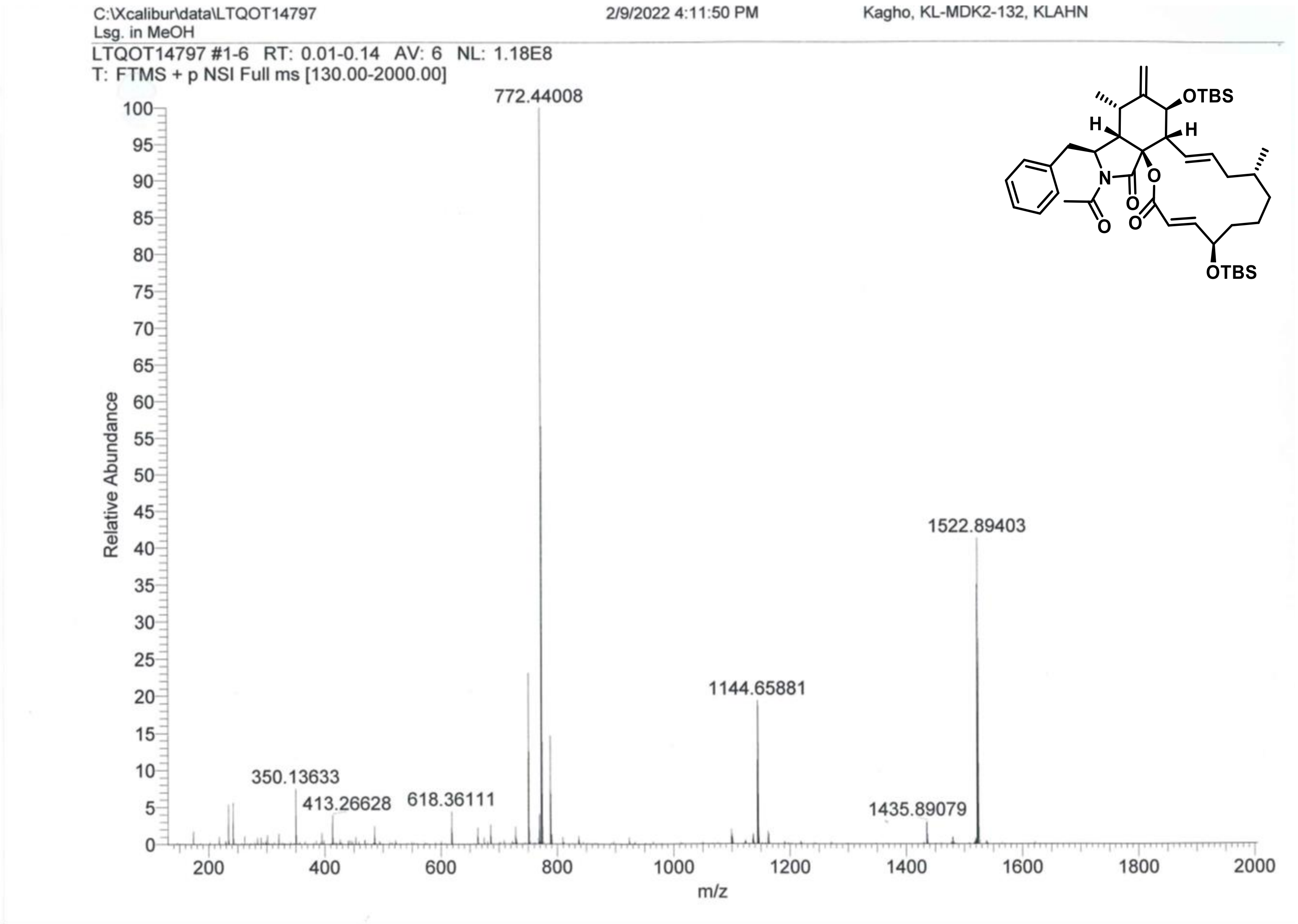

Figure S28: Compound **11** (**HRMS** (ESI))

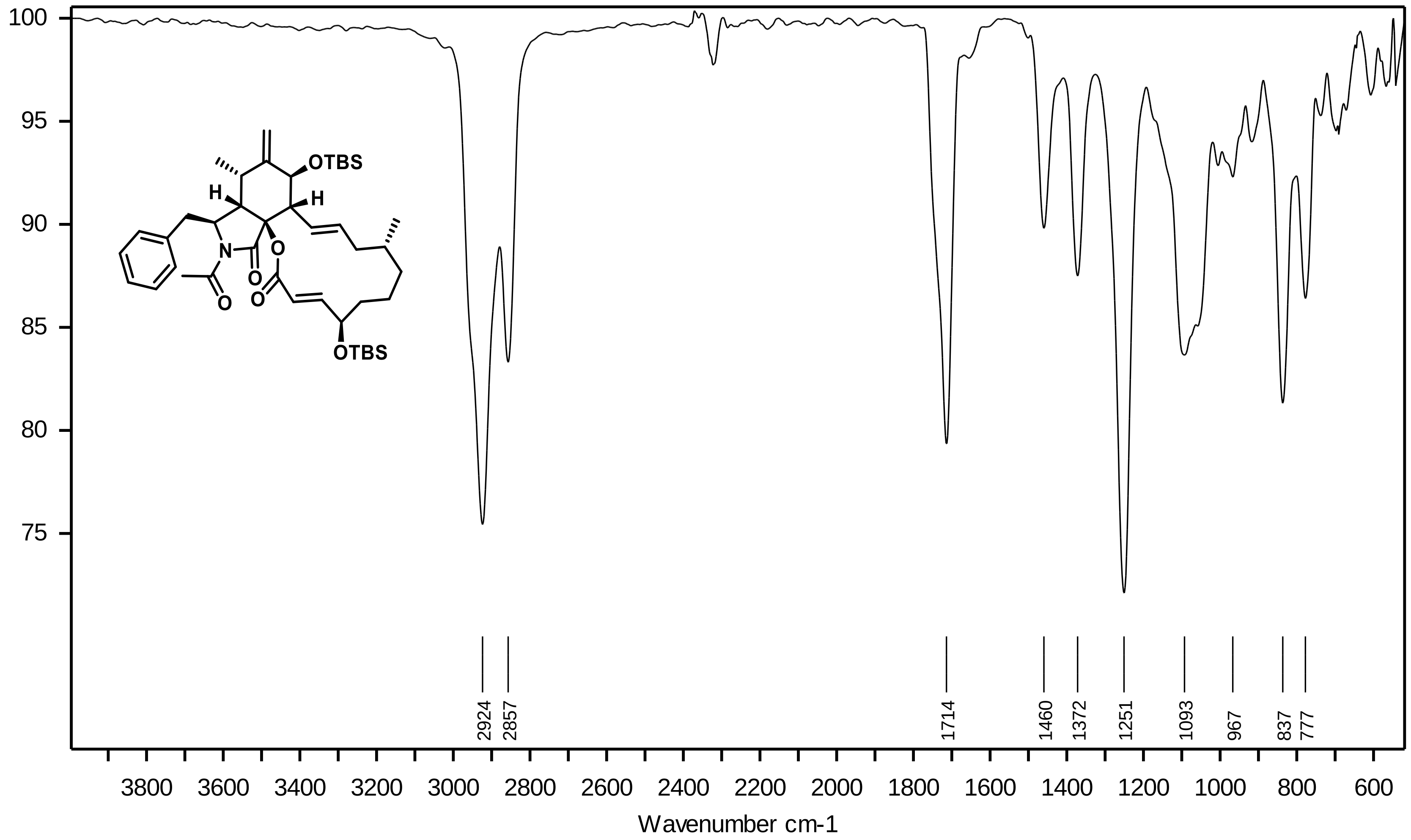

Figure S29: Compound **11** (**IR** (ATR))

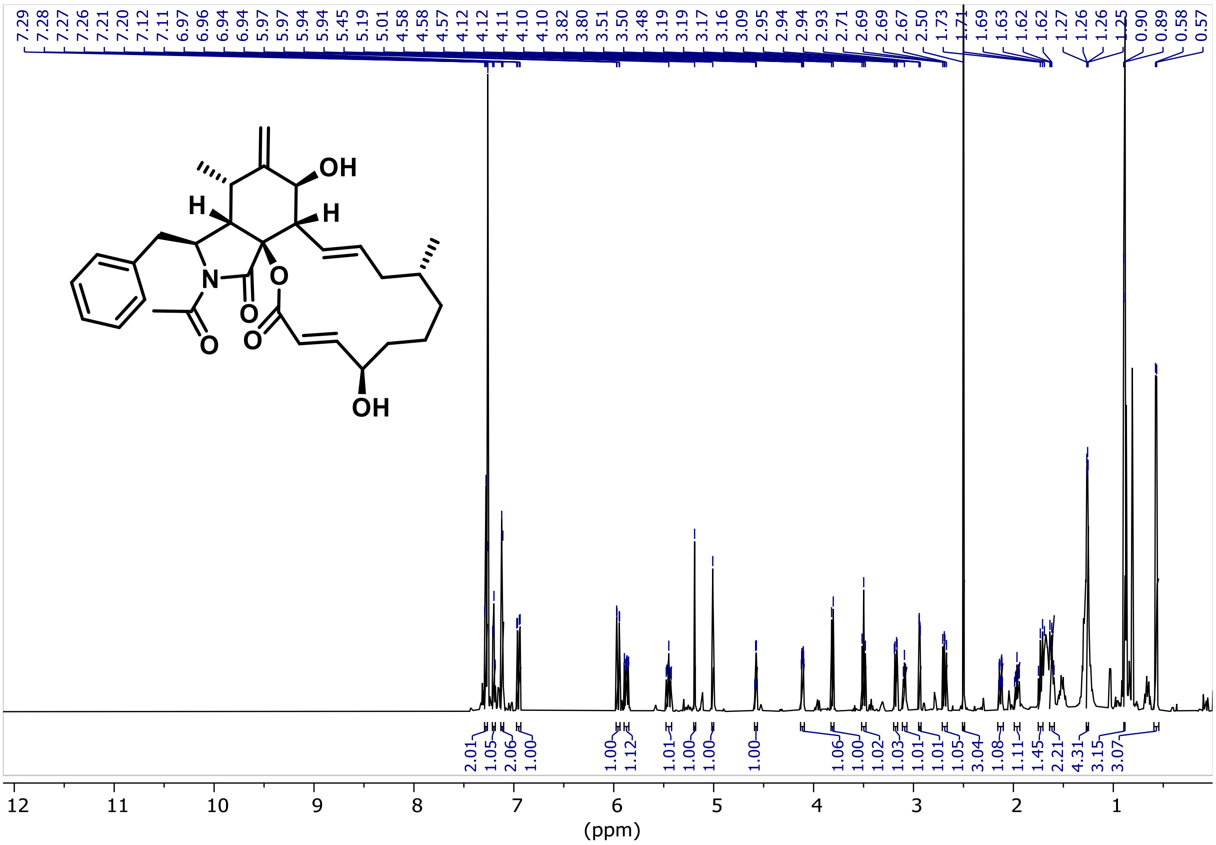

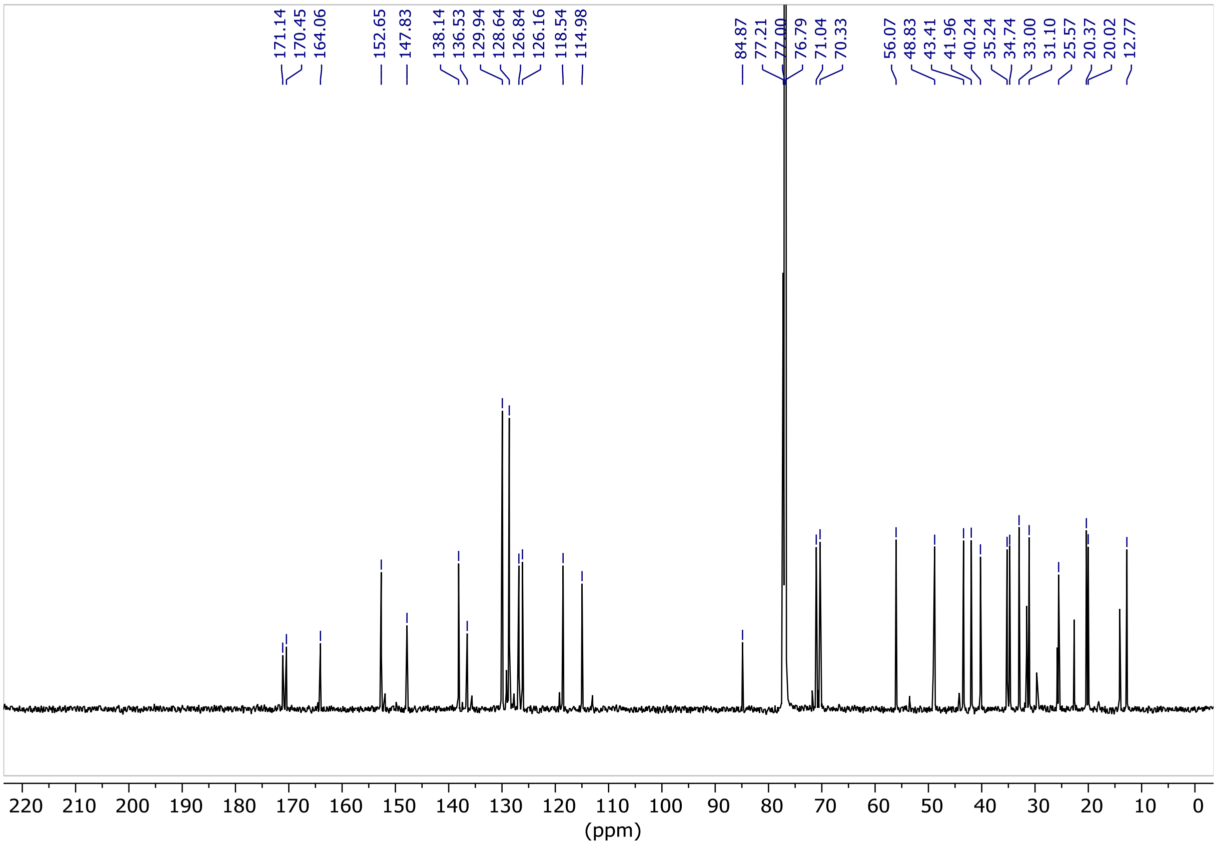

Figure S30: Compound **5** (^{1}**H-NMR** (600 MHz, $CDCl_3$) and ^{13}**C-NMR** (151 MHz, $CDCl_3$))

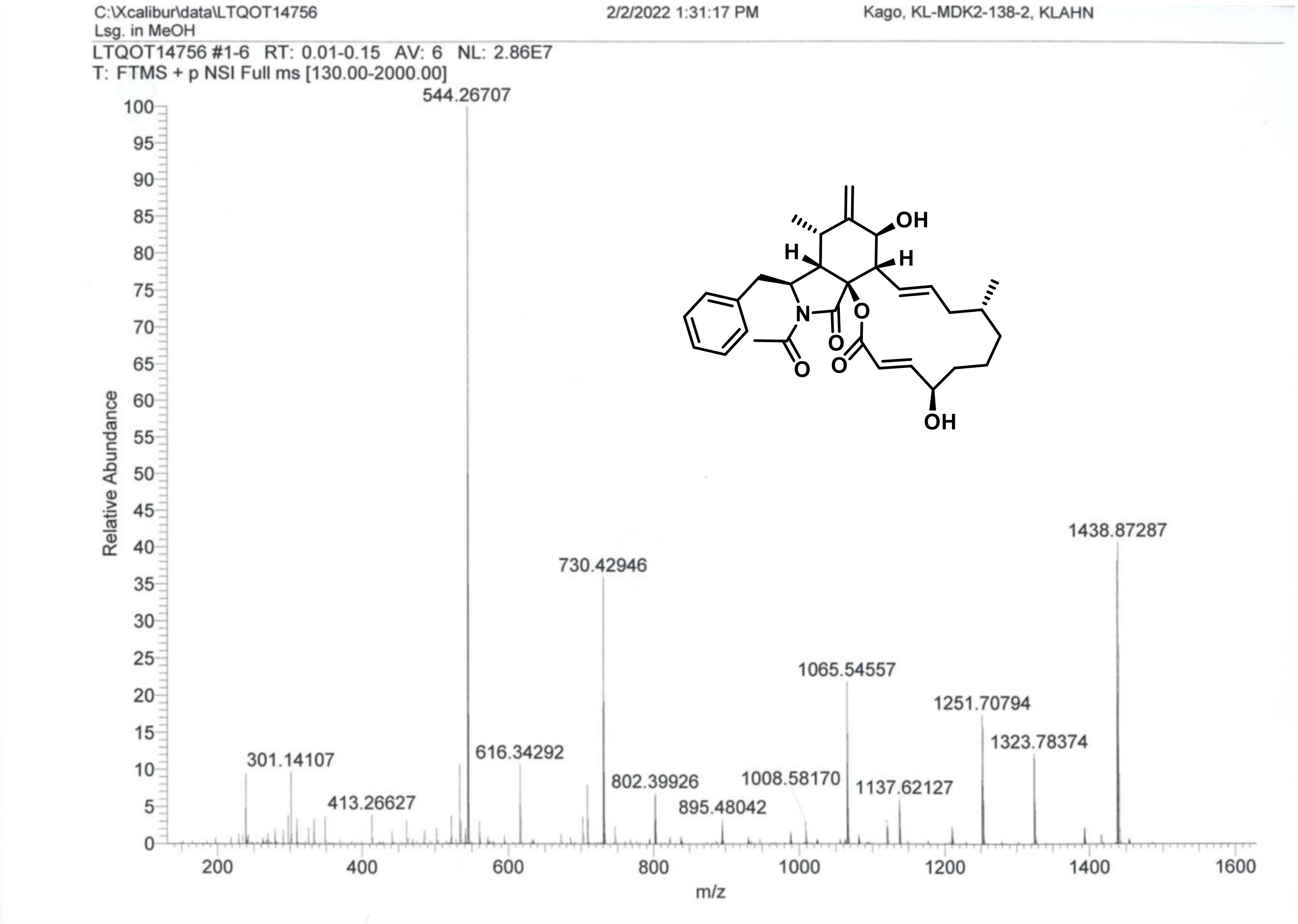

Figure S31: Compound **5** (**HRMS** (ESI))

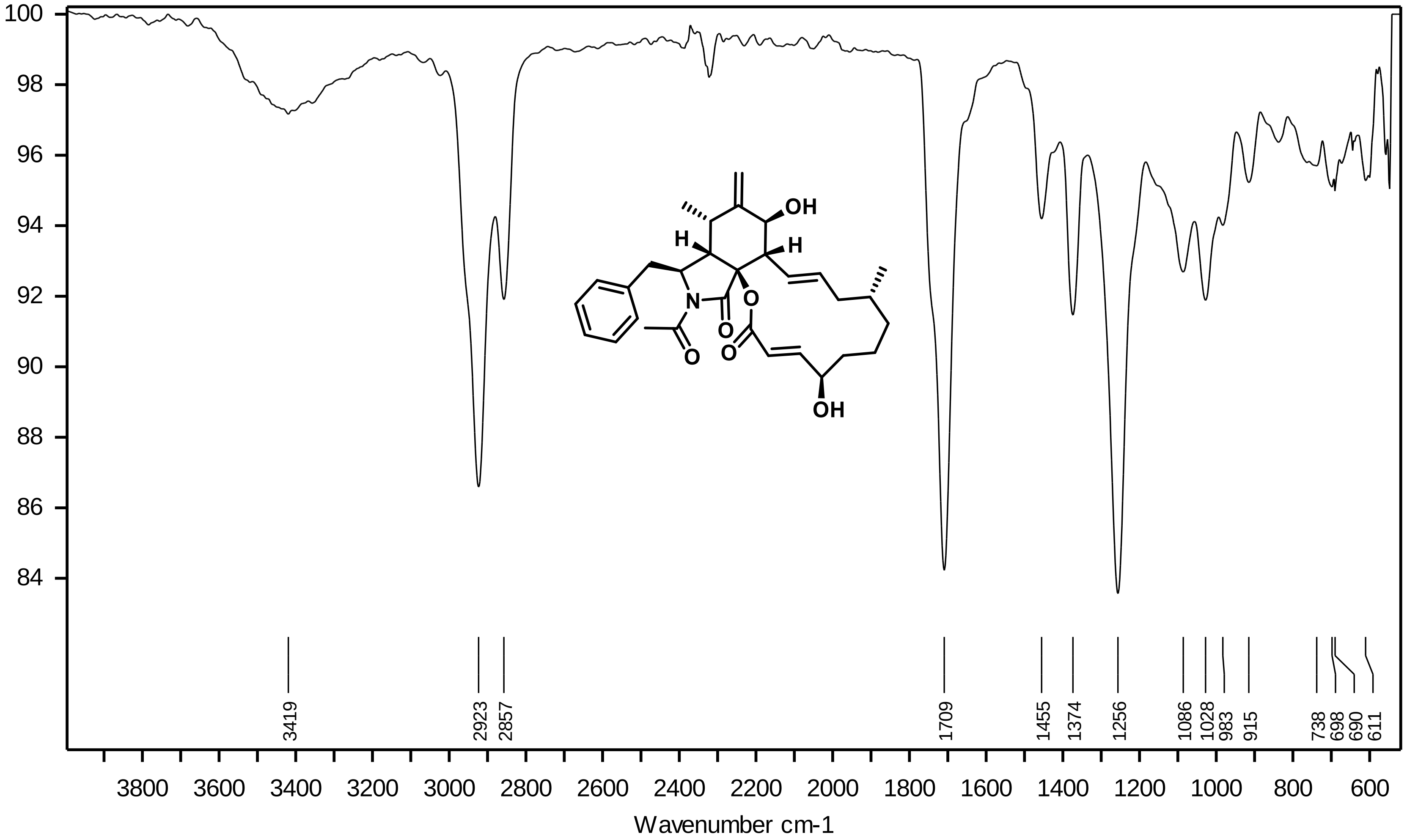

Figure S32: Compound **5** (**IR** (ATR))

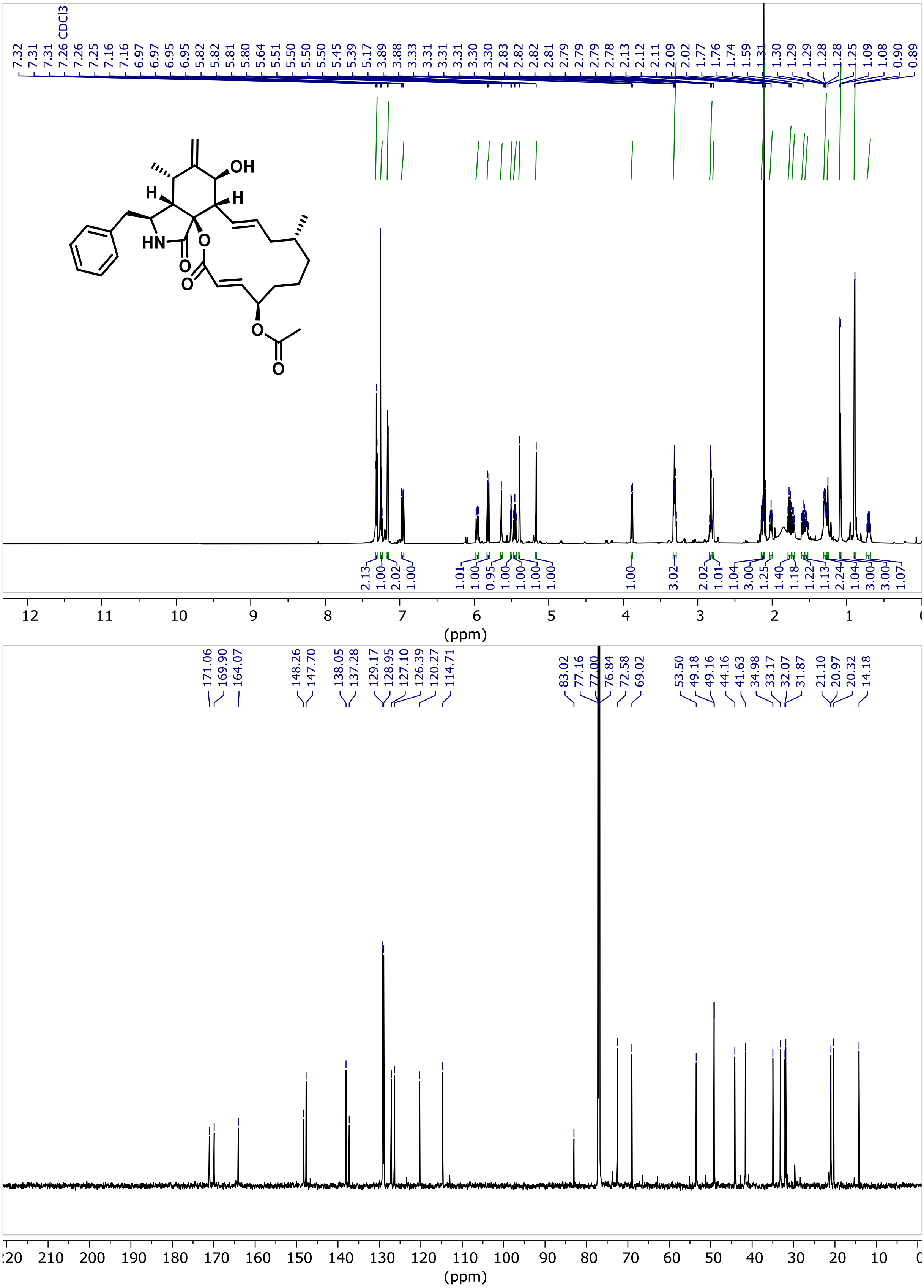

Figure S33: Compound **6** ([1]**H-NMR** (800 MHz, $CDCl_3$) and [13]**C-NMR** (201 MHz, $CDCl_3$))

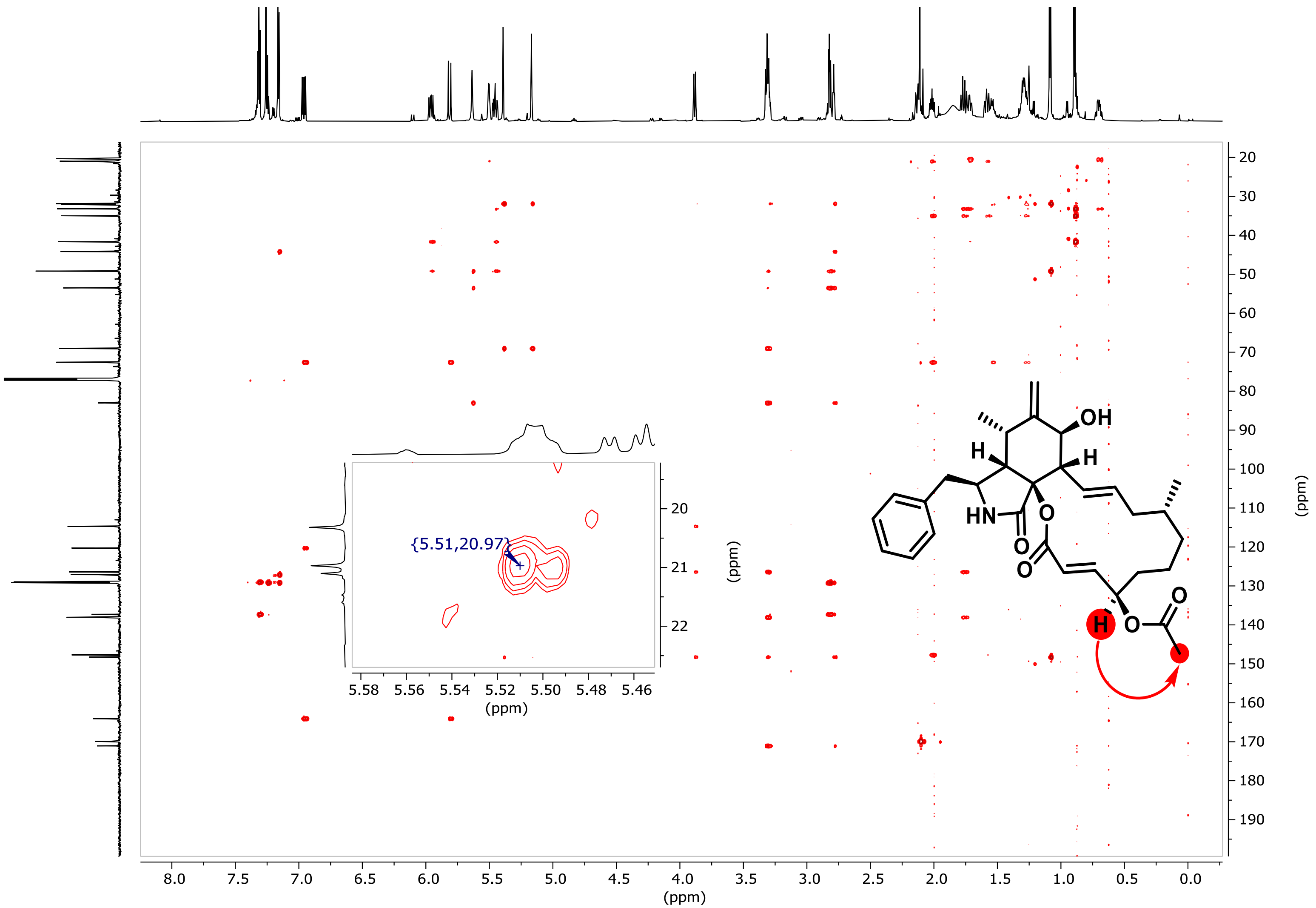

Figure S34: Compound **6** (**HMBC** (800 MHz, $CDCl_3$))

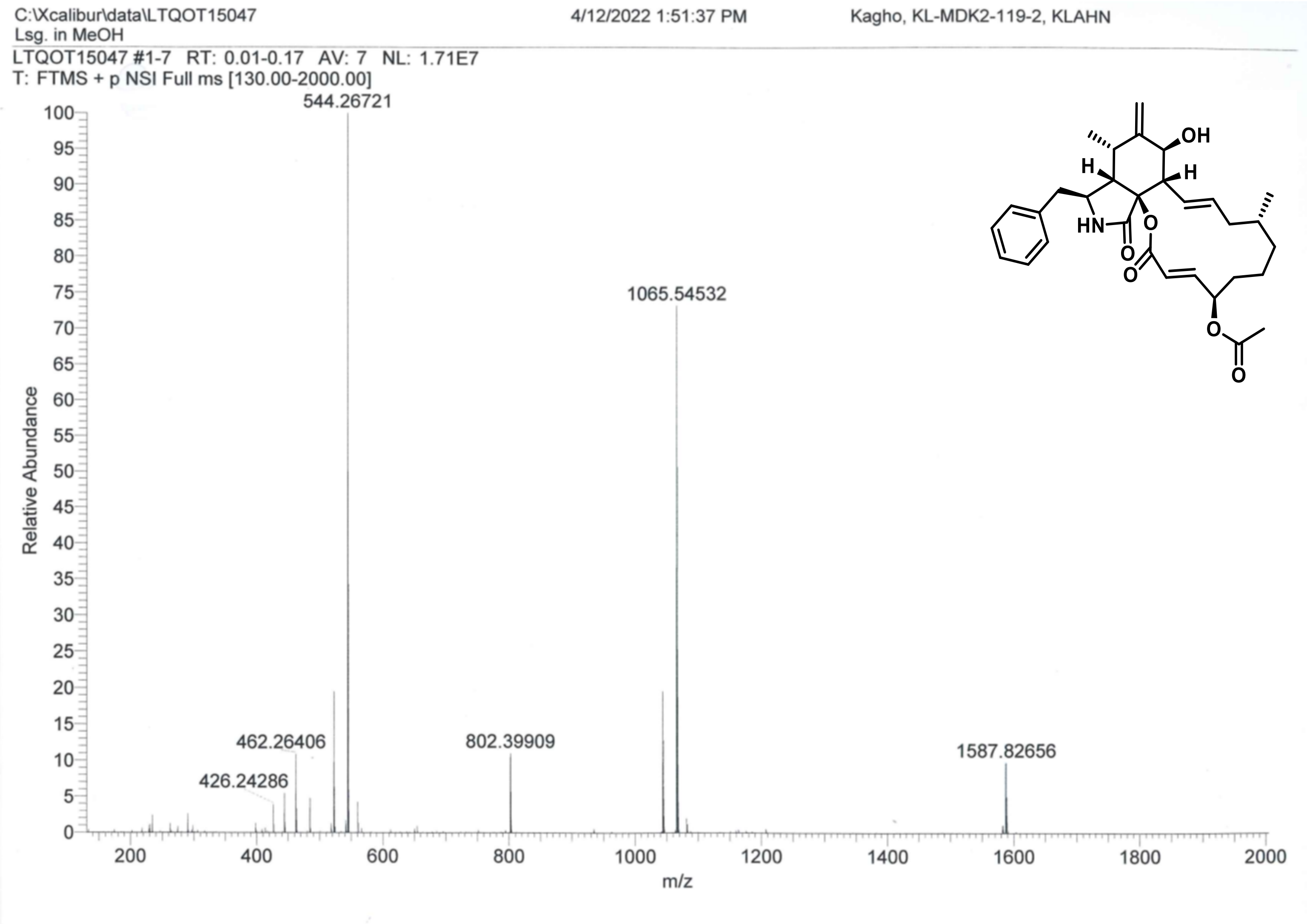

Figure S35: Compound **6** (**HRMS** (ESI))

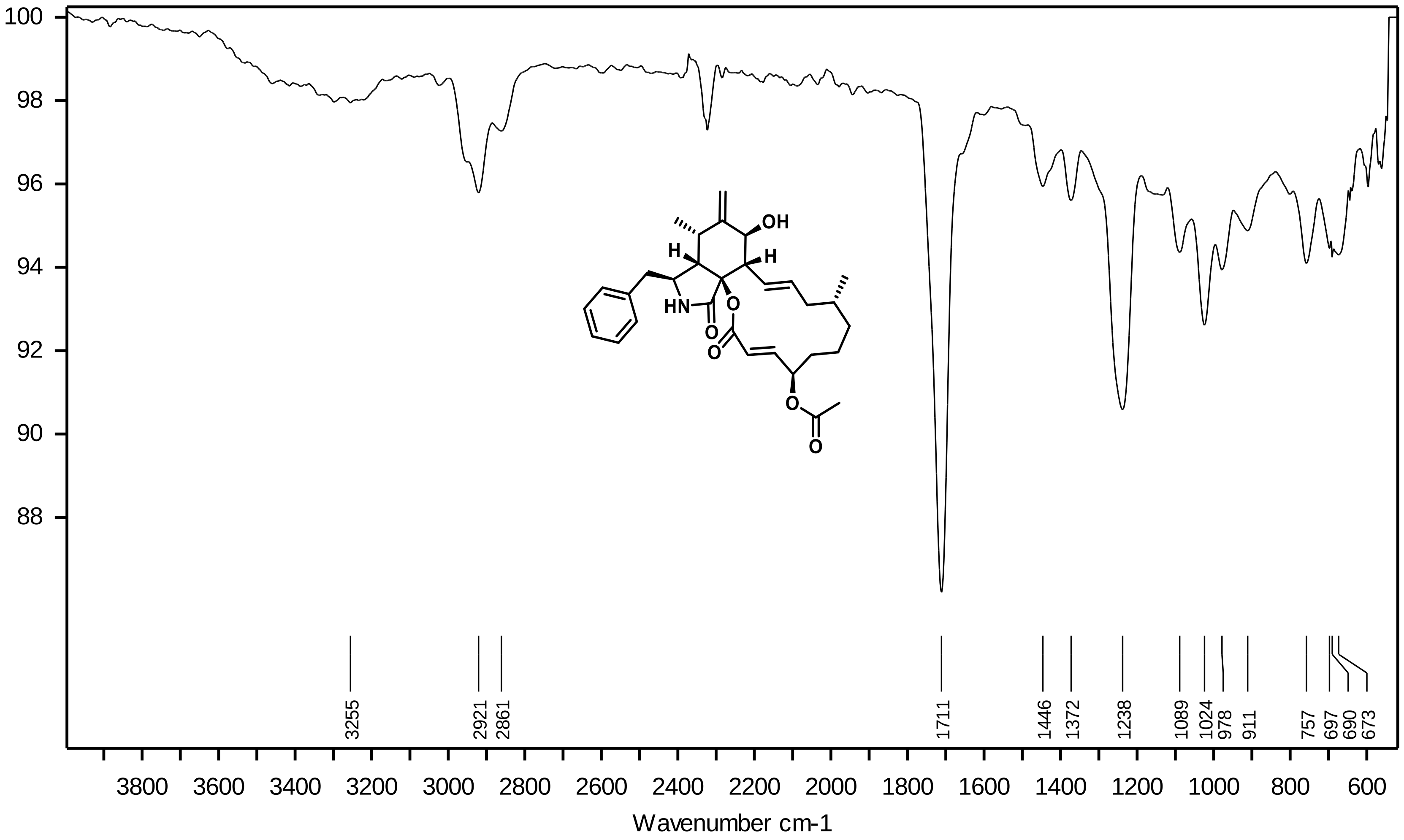

Figure S36: Compound **6** (**IR** (ATR))
